# RSPSSL: A novel high-fidelity Raman spectral preprocessing scheme to enhance biomedical applications and chemical resolution visualization

**DOI:** 10.1038/s41377-024-01394-5

**Published:** 2024-02-20

**Authors:** Jiaqi Hu, Gina Jinna Chen, Chenlong Xue, Pei Liang, Yanqun Xiang, Chuanlun Zhang, Xiaokeng Chi, Guoying Liu, Yanfang Ye, Dongyu Cui, De Zhang, Xiaojun yu, Hong Dang, Wen Zhang, Junfan Chen, Quan Tang, Penglai Guo, Ho-Pui Ho, Yuchao Li, Longqing Cong, Perry Ping Shum

**Affiliations:** 1 State Key Laboratory of Optical Fiber and Cable Manufacture Technology, Guangdong Key Laboratory of Integrated Optoelectronics Intellisense, Department of EEE, Southern University of Science and Technology, Shenzhen, 518055 China; 2 College of Optical and Electronic Technology, China Jiliang University, Hangzhou, 310018 China; 3 Department of Nasopharyngeal Carcinoma, Sun Yat-sen University Cancer Center, State Key Laboratory of Oncology in South China, Collaborative Innovation Center for Cancer Medicine, Guangdong Key Laboratory of Nasopharyngeal Carcinoma Diagnosis and Therapy, Guangzhou, 510060 China; 4 Department of Ocean Science and Engineering, Southern University of Science and Technology, Shenzhen, 518055 China; 5 Department of Nephrology, Chaozhou People’s Hospital, Chaozhou, 521011 China; 6 Clinical Research Design Division, Sun Yat-sen Memorial Hospital, Guangzhou, Guangdong, 510120 China; 7 School of Automation, Northwestern Polytechnical University, Xi’an, Shaanxi 710072 China; 8 Department of Biomedical Engineering, The Chinese University of Hong Kong, Hong Kong, China; 9 Guangdong Provincial Key Laboratory of Nanophotonic Manipulation, Institute of Nanophotonics, Jinan University, Guangzhou, 511443 China

**Keywords:** Biophotonics, Nonlinear optics

## Abstract

Raman spectroscopy has tremendous potential for material analysis with its molecular fingerprinting capability in many branches of science and technology. It is also an emerging omics technique for metabolic profiling to shape precision medicine. However, precisely attributing vibration peaks coupled with specific environmental, instrumental, and specimen noise is problematic. Intelligent Raman spectral preprocessing to remove statistical bias noise and sample-related errors should provide a powerful tool for valuable information extraction. Here, we propose a novel Raman spectral preprocessing scheme based on self-supervised learning (RSPSSL) with high capacity and spectral fidelity. It can preprocess arbitrary Raman spectra without further training at a speed of ~1 900 spectra per second without human interference. The experimental data preprocessing trial demonstrated its excellent capacity and signal fidelity with an 88% reduction in root mean square error and a 60% reduction in infinite norm (${L}_{{\infty }}$) compared to established techniques. With this advantage, it remarkably enhanced various biomedical applications with a 400% accuracy elevation (ΔAUC) in cancer diagnosis, an average 38% (few-shot) and 242% accuracy improvement in paraquat concentration prediction, and unsealed the chemical resolution of biomedical hyperspectral images, especially in the spectral fingerprint region. It precisely preprocessed various Raman spectra from different spectroscopy devices, laboratories, and diverse applications. This scheme will enable biomedical mechanism screening with the label-free volumetric molecular imaging tool on organism and disease metabolomics profiling with a scenario of high throughput, cross-device, various analyte complexity, and diverse applications.

## Abstract (web summary)

A novel generic high-fidelity Raman spectral denoising and baseline correction strategy to enhance diverse cross-device/specimen biomedical applications and hyperspectral image chemical resolution visualization to reveal spatial features of cancer tissue.

## Graphical abstract

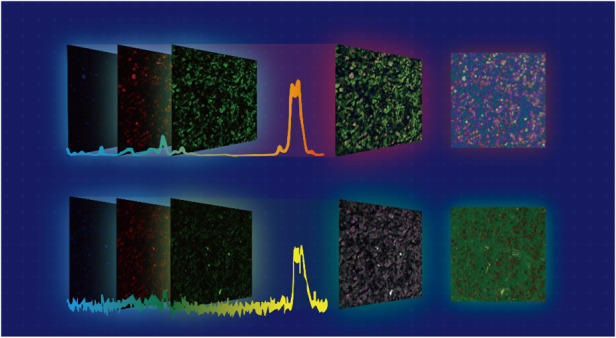

## Introduction

Raman spectroscopy is an interdisciplinary analysis technique based on spectra detection, chemometrics, and informatics^1^. It provides volumetric chemical-specific information in a non-destructive and label-free manner, is valuable in characterizing biological and material samples, and has become an emerging omics technology for clinical research and applications^2–7^. It also showed tremendous value in label-free rapid intraoperative diagnosis^8–11^. However, its analysis is inevitably hampered by noise and slowly fluctuating background signals^12,13^. Noise signals typically come from cosmic rays, ambient light, and the dark current from devices. The slowly fluctuating background signals caused by residual Rayleigh scattering and fluorescence signals are also called baseline signals^12^. This interference affects the intensity of Raman peaks and distorts their shapes^14^. Spectral preprocessing, including denoising and baseline corrections, is the first step and one of the predominant challenges for complex analyte analysis, cross-device, and high throughput applications.

In essence, the spectral noise refers to higher frequency information than ideal Raman peaks, and the baseline signals refer to the trend line with lower frequency information, which usually conceals the actual Raman signal (Fig. S2). Over the decades, mathematical methods, including iterative fitting and frequency domain-based methods, have been used in Raman spectral denoising and baseline corrections. In brief, the iterative fitting includes Polynomial fitting^15^, Iterative morphological operations^16^, and Penalized least squares^17^. The commonly applied Polynomial fitting method functions by iterative fluctuating signal elimination. Iterative morphological operations and Penalized least squares work by iterative baseline fitting based on spectral trends. However, these methods require a manual selection of iteration cycles to adjust their fitting ability. The representative frequency domain-based methods are the Fourier transform^18^ and Wavelet transform^19^. The Fourier transform converts spectra into frequency-domain signals to isolate noise and baselines from Raman peaks. However, due to the lack of periodic changes in the entire spectra, this method cannot decompose the frequency of each wavenumber signal in all Raman shifts. It has a limited effect in full-length spectral preprocessing compared to Wavelet transform. The alternative method, Wavelet transform, uses a frequency series decomposition scheme to achieve higher analytical resolution with a locally adaptive transformation strategy. Although Wavelet transform has a better preprocessing effect, this method still requires manual selection of the series number for different spectral partitions. In summary, math-based preprocessing methods are unsuitable for high throughput and cross-device applications but are valuable in developing new methods.

Deep learning-based spectral preprocessing schemes offer an appealing approach to auto-extract spectral features and distinguish Raman peaks from noises and baselines in a data-driven manner^20^. Within these approaches, neural networks were established to learn the nonlinear mappings from the given input (raw spectra) and label (ideal spectra) pairs, then generate internal criteria for spectral preprocessing. It solves the complex inverse problem of noise and baseline signal deprivation. Recently, Kung^21^ and Liu^22^ et al. used Lorentzian peaks and analog noise, like Gaussian white noise, to generate simulative spectral pairs for ResNet model training, showing a better spectral preprocessing result than mathematical methods. Additionally, Chen^23^ et al. combined the UNet structure with a ResNet network to improve the abstraction ability of high-level features during Downsampling. This modification improves the spectral preprocessing fidelity.

Although deep learning-based methods showed higher efficiency than conventional mathematical methods, they were not widely adopted. Unsatisfied spectral fidelity and preprocessing capacity in cross-device, cross-sample, and cross-spectral type applications are the barriers to universal adoption. The performance of supervision-based neural networks relies on the quality and quantity of training data^13,20,24,25^. However, ideal spectra without noises and baselines cannot be experimentally obtained. The recently reported training data are mathematical simulative spectra. Their Raman peaks, noises, and baselines are derived from Lorentzian peaks, Gaussian white noises, and multipoint fitting functions. Unfortunately, real Raman spectra have more features derived from diverse instruments and samples that are hard to mimic by mathematical methods^26^ (Fig. S2), which leads to unsatisfied spectral fidelity. Therefore, researchers obtained abundant experimental training spectra and averaged thousands of repeatedly collected spectra as ideal spectra (ground truth) in coherent Raman hyperspectral imaging to achieve higher spectral fidelity^20^. However, a new training dataset is required when transferring to another application with a different sample type or device. As far as we know, this method has yet to be reported in spontaneous Raman spectroscopy due to the unaffordable time consumption and the lack of baseline correction capacity (Fig. S1). To solve this problem, we propose to generate a high-diversified training dataset for cross-applications from actual and wide-source Raman spectra as part of our self-supervised learning preprocessing scheme. Since Raman spectra follow the spectral-physical composition relationships, we will design a chimera network combining physical equations and neuron networks to intelligently learn the features from actual spectra under the guidance or restriction of physical laws of Raman spectra. Moreover, the potential of generative adversarial neural networks (GAN) theory in data generation inspired us in the spectral diversity amplification^26–30^. The proposed auxiliary task, Raman spectral generation adversarial network (RSGAN), aims to transform finite real spectra into amplified high-diversity and high-fidelity spectra with paired ideal spectra as labels.

Except for the quality and diversity of the primary training dataset generation, a model with remarkably improved feature learning ability is also critical for the spectral preprocessing capacity. Here, we proposed a novel Raman spectral preprocessing model, Raman spectral Background-Estimation-Patches convolutional neural network (RSBPCNN), with a dedicated structure according to the inherent characteristics of Raman spectra. Its first pipeline uses a patches strategy to avoid intensity interference from the baselines before Raman peak feature abstraction. The second pipeline preliminarily recognizes noises and baselines as part of the self-supervision strategy. These two pipelines merge into a well-designed Downsampling-Upsampling submodule for multi-scale feature extraction and reconstruction. After training with the RSGAN-generated data, the parameters will self-optimize with a double-loss function system. This scheme enables parameter self-optimization without human intervention. It can function independently in arbitrary cross-device spectral denoising and baseline corrections without further data training. Except for the experimental data trial, we will validate its capacity in various applications using different spectral types, devices, excitation wavelengths, and sample types in multiple laboratories.

## Results

### Research pipeline

**Fig. 1:**
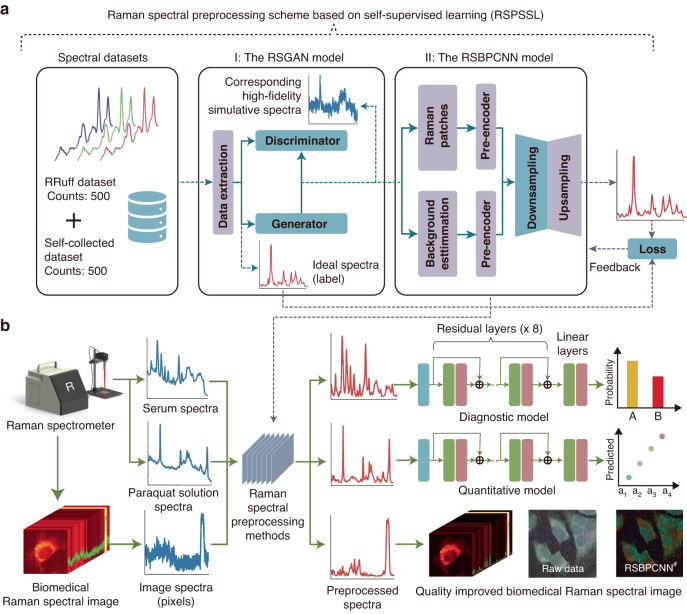
Schematics of this work: The proposed self-supervised learning-based Raman spectral preprocessing (RSPSSL) scheme and the experimental design for its comprehensive performance evaluation. **a** The general pipeline of the RSPSSL scheme includes spectral training datasets, an RSGAN model, and an RSBPCNN model. **b** Experimental design for the spectral preprocessing performance evaluation through multiple biomedical applications, including cancer diagnosis, paraquat concentration quantification, and Raman hyperspectral image preprocessing

The proposed RSPSSL scheme consists of original training datasets with actual Raman spectra from various analytes and devices with or without Raman enhancement, an auxiliary task model RSGAN for high-fidelity labeled spectra creation, and a multiscale feature fitting spectral preprocessing model RSBPCNN (Fig. 1a, b). We evaluated the scheme performance through an experimental data preprocessing trial and the contributions of RSPSSL (RSBPCNN^#^) in various biomedical applications, including cancer diagnosis (classification), paraquat concentration quantification, and hyperspectral image preprocessing (Fig. 1b). These results will compare with established typical spectral preprocessing methods, such as Polynomial fitting, Wavelet transform, Residual CNN^*^ (Fig. S23), and UNet-1D^*^ (Fig. S24).

### Auxiliary task: The RSGAN Model

**Fig. 2:**
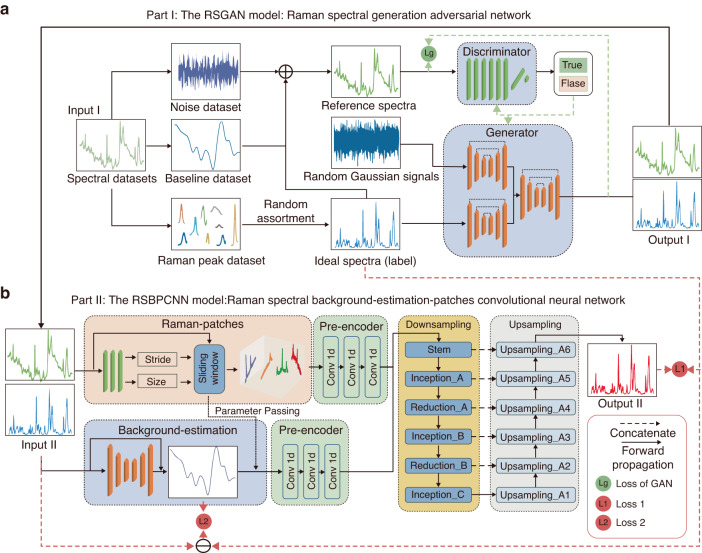
The design of the proposed RSPSSL scheme. **a** Schematic diagram of the auxiliary task model RSGAN. **b** Schematic diagram of the spectral preprocessing model RSBPCNN

To create labeled high-quality spectra, we designed the RSGAN model with a spectra decomposition and reconstruction strategy to generate ideal spectra as labels and a personalized GAN submodule to create simulative spectra with high fidelity and diversity (Fig. 2a). In the beginning, a total of 1 000 randomly selected Raman spectra from the RRUFF and self-collected dataset are decomposed into 1 000 sets of noises and baselines and 6 000 Raman peaks, respectively. During this process, we isolate noise and baseline signals using Wavelet transform and separate Raman peaks by a differential method. Later, the labels, 10 000 ideal spectra without noise and baseline, were randomly assembled as 5–20 Raman peaks per spectrum. Simultaneously, their corresponding reference spectral dataset is synthesized by a random combination of ideal spectra, extracted noises, and baseline signals. By controlling the superposition coefficient of the noise and baseline signals in synthetic Raman spectra, diverse signal-to-noise ratios (SNR) and baselines are created (Fig. S4). This process can synthesize many reference spectra with corresponding ideal spectra as labels. The more diverse original spectra used, the better spectral fidelity to achieve. Finally, the GAN submodule, consisting of a generator and a discriminator, is involved in this data enhancement process. In detail, the generator uses ideal spectra and random Gaussian signals as input to produce primary analog spectra by varying the random Gaussian signals. The discriminator distinguishes synthetic reference spectra from generator-produced spectra until they are too similar to identify. After training, the high-fidelity and diversified property of the output labeled simulative spectra are endowed by the confrontation of the generator and discriminator. In this submodule, the UNet network is modified into UNet-1D to absorb its excellent detail-feature reconstruction ability (Fig. S3). Here, we built three UNet-1D blocks instead of a simple CNN algorithm in the generator block. The discriminator is a classifier for judgment, so we apply a modified ResNet-1D block to achieve excellent classification accuracy^31^. The RSGAN model structure, loss function, and training process are provided in the supplementary information (Fig. S3).

### The spectral preprocessing model: RSBPCNN

The RSBPCNN model requires a higher multi-scale feature abstraction ability to adapt the diversified property of training spectra. The proposed Raman-Patches and Background-Estimation with Pre-Encoder blocks are critical designs to improve the fitting ability and efficiency (Fig. 2b). The self-constructed Downsampling and Upsampling submodules are the core of this model for multiple feature extraction. During the training process, we input RSGAN-generated spectra in parallel to the Raman-Patches and Background-Estimation submodules and then output the data to their respective Pre-Encoder submodules for parameter consistency adjustment. Their subsequent output data is subtracted and imported into the Downsampling-Upsampling block for spectral feature abstraction and reconstruction. Finally, we compare the output preprocessed spectra with corresponding ideal spectra (label) and baseline outlines (products of the Background-Estimation block) for automatic parameter optimization with a double-loss function system. Until now, the well-trained RSBPCNN model (RSBPCNN^*#*^) can be used directly without additional training for arbitrary Raman spectra preprocessing obtained from different devices.

For details of the model design, the Raman-Patches submodule functions in splitting input spectra into partitions by a sliding window method, with window stride length and step size as variable parameters (Fig. S5). This submodule uses a small-unit strategy to enhance the spectral fitting ability. Parameters of window stride length and step size are tunable during the RSBPCNN model training. Once accomplished, both parameters become fixed for all spectral preprocessing. In parallel, the Background-Estimation submodule preliminarily fits the noise and baseline signals of the input spectra by contour extraction. It uses a U-shape structure with three pairs of convolution and deconvolution networks, further improving the baseline correction ability (Fig. 2b, S6). These two parallel pipelines link to each Pre-encoder submodule for the output data parameter adjustment before passing them to the down-strain submodules like Downsampling and Upsampling submodules. The Pre-encoder submodule also consists of three convolutional layers to keep consistency.

The high-level feature extraction and reconstruction mainly depend on the Downsampling and Upsampling submodules. Since the reported ResNet model contains only one Downsampling module that may cause information loss^23,32^, we construct a U-shaped Down-Upsampling submodule with an Inception network to avoid this problem. This structure has parallel connections between the previous and sequential layers (Fig. S6). Each layer learns the sparse features with larger kernels and the non-sparse features from smaller kernels. The design with multiple kernel sizes enhances the multi-scale feature learning ability of the Inception network. The Downsampling submodule includes Stem, Inception_A, Inception_B, Inception_C, Reduction_A, and Reduction_B subnetworks (Fig. S6). Spectra in these neural networks are all matrix operations. The corresponding subnetwork modulates the channel and feature dimensions of each spectrum. The Stem subnetwork creates features and channels by 1/4 and 4 times the input. The Inception_A, Inception_B, and Inception_C subnetworks maintain the dimensionality of input data. The Reduction_A and Reduction_B halve the features and double the channels. This Downsampling submodule extracts wrought multi-scale spectral features through the above subnetworks to provide information for the subsequent Upsampling submodule^33^. The Downsampling submodule utilizes Inception, rather than a simple stack of convolutional layers, to increase the feature extraction granularity and hierarchy.

The Upsampling submodule is devoted to reconstructing the preprocessed spectra with features obtained by the Downsampling submodule, which are output data of the RSBPCNN model. These restored spectra removed noise, and baseline signals will benchmark to the ideal spectra (label) and the contour of the background signal extracted by the Background-Estimation submodule during model training to optimize the fitting parameters. In the Upsampling submodule, we use one-dimensional transposed convolutional layers (ConvTranspose1d) and one-dimensional convolutional layers (Conv1d) to maintain the dimensional consistency with the Downsampling submodule (Fig. S6). In summary, the strategy of this U-shaped Down-Upsampling submodule is to enhance crucial features like Raman peaks and weaken invalid signals such as noise and baseline signals of the preprocessed spectra.

The training process of the RSBPCNN model is divided into two steps with two loss functions, which are defined as,

1 $${L}_{{BE}}(x,y)=\frac{1}{n}{\sum }_{i}{z}_{{BE}-i}$$

2 $$L(y)=\lambda \frac{1}{n}{\sum }_{i}{z}_{i}+(1-\lambda ){L}_{{BE}}(x,y)$$

3 $${z}_{{BE}-i}=\left\{\begin{array}{c}0.5{[B({x}_{i})-{y}_{i}+{x}_{i}]}^{2},{if}\left|{x}_{i}-{y}_{i}\right| \,<\, 1\\ \left|B({x}_{i})-{x}_{i}-{y}_{i}\right|-0.5,{ot}h{erwise}\end{array}\right.$$

4 $${z}_{i}=\left\{\begin{array}{c}0.5{[P({x}_{i})-{y}_{i}]}^{2},{if}\left|{x}_{i}-{y}_{i}\right| \,<\, 1\\ \left|P({x}_{i})-{y}_{i}\right|-0.5,{ot}h{erwise}\end{array}\right.$$

${L}_{{BE}}(x,y)$ is the loss function for Background Estimation submodule training, and the $L(y)$ function is applied to train the entire model. ${x}_{i}$ and ${y}_{i}$ refer to the RSGAN-generated high-fidelity simulative and corresponding ideal spectra, respectively. $B({x}_{i})$ and $P({x}_{i})$ correspond to the output of the Background Estimation submodule and the RSBPCNN model, respectively. Since the RSGAN-generated high-fidelity simulative spectra have severe baseline shifts and fluctuations compared to synthesized ideal spectra, the loss function here is designed based on the Huber loss function. It converges faster than the mean absolute error (MAE) index and is not as sensitive as root mean square error (RMSE) to outliers during training. The training sequence goes firstly to the Background Estimation submodule and then the entire RSBPCNN model to maximize the preprocessing effect. A weight coefficient $\lambda$ is employed to balance the feedback of these two pipelines. When the value of $\lambda$ increases, the Background-Estimation submodule weighs less in the training procedure, and vice versa. The preprocessing effect of the RSBPCNN^#^ model is optimal when $\lambda =0.2$.

### Experimental data preprocessing trial: Capacity and fidelity of the RSPSSL scheme

We evaluate the performance of the RSPSSL scheme and compare it to the established spectral preprocessing techniques, including Polynomial fitting and Wavelet transform as typical mathematical methods, Residual CNN (ResNet) and UNet-1D (ResNet + UNet) as representatives of deep learning methods. The details for the above models, including RSBPCNN, can be found in the supplementary information (Fig. S23, 24). Additionally, since the training data quality is a critical impact factor for deep learning performance, we will investigate the influence of training datasets, such as mathematical simulative spectra (marked as ^*^) and RSGAN-generated data (marked as ^#^) in Residual CNN, UNet-1D, and RSBPCNN. Moreover, the accuracy metrics will include RMSE and the infinite norm (${L}_{{\infty }}$) in this trial. RMSE is a measurement of the numerical difference that represents a perception estimation of the spectral similarity to the corresponding ideal spectra, which indicates the denoising and baseline correction effect. Meanwhile, ${L}_{{\infty }}$ is a target metric that focuses on the intensity between Raman peaks, suggesting spectral intensity fidelity. These indicators all negatively correlate to the spectral preprocessing capacity.

**Fig. 3:**
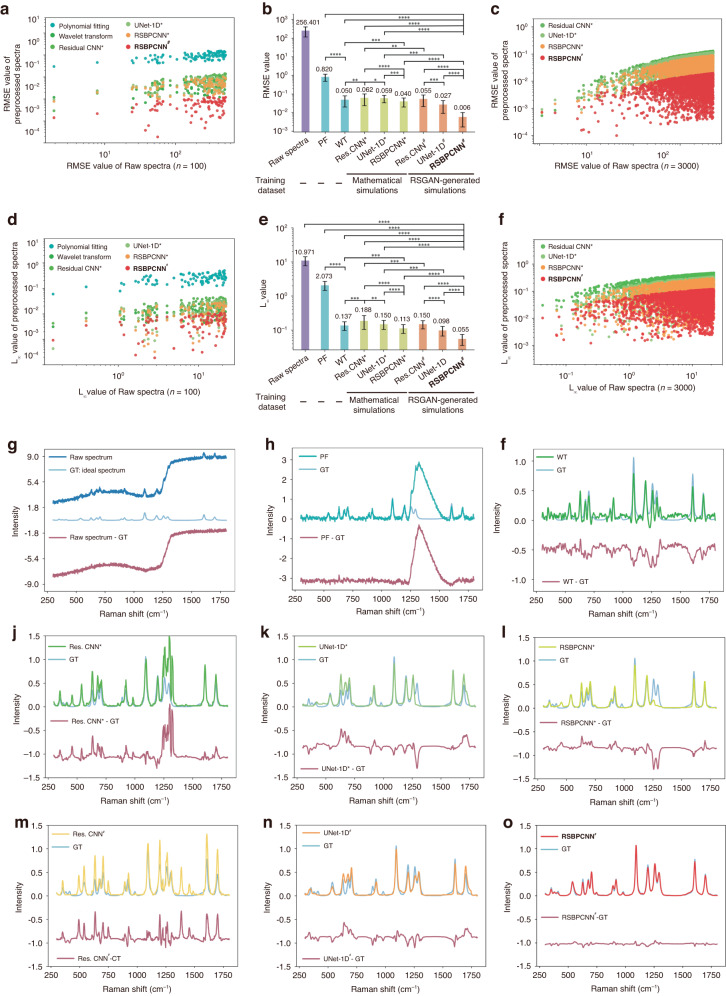
Results of the experimental data preprocessing trial. **a**–**f** Summary of the Raman spectral preprocessing effect evaluation matrix of different methods (**a**, **b**, **d**, **e**: *n* = 100 spectra; **c**, **f**: *n* = 3 000 spectra). **a**, **d** Scatter diagrams show the RMSE and ${L}_{{\infty }}$ level of preprocessed spectra (*n* = 100), which reflect the denoising and baseline correction effects of different methods. Y-axis: mean ± SD of the RMSE or ${L}_{{\infty }}$ values of preprocessed spectra with different spectral preprocessing methods; X-axis: Raw spectra are indicated by their original RMSE or ${L}_{{\infty }}$ values. **b**, **e** Histograms of **a,** **d** with mean ± SD. Statistical significance was calculated using the Wilcoxon signed-rank test for two correlated samples. An asterisk * indicates *P* < 0.05, ** indicates *P* < 0.01, *** indicates *P* < 0.001, **** indicates *P* < 0.0001. **c**, **f** Scatter diagrams show the RMSE and ${L}_{{\infty }}$ level of preprocessed spectra (*n* = 3 000) with different deep learning-based preprocessing methods. Histograms of **c**, **f** are shown in Fig. S10. **g** Visualization of a randomly selected raw spectrum (navy blue, RMSE of 227.89) and its corresponding ideal spectrum (baby blue). The roseate curve subtribes the raw spectrum and the ideal spectrum, reflecting the spectral fidelity. **g**–**o** Visualization of the preprocessed spectra using Polynomial fitting (PF), Wavelet transform (WT), Residual CNN^*^, UNet-1D^*^, RSBPCNN^*^, Residual CNN^#^, UNet-1D^#^, and RSBPCNN^#^, respectively. A superscript asterisk ^*^ indicates that the training dataset of this model is mathematical simulative spectra. A superscript pound sign ^#^ indicates that the training dataset of the model is RSGAN-generated spectra. Res. CNN: Residual CNN. GT: Ground truth

The independent validation dataset contains 100 or 3 000 randomly selected RSGAN-generated simulative spectra with labels of corresponding ideal spectra as ground truth (Fig. 3a–f, S10). Since mathematical preprocessing methods require manual parameter adjustment for each spectrum, we use 100 randomly selected spectra as validation data (Fig. 3a, b, d, e). Deep learning preprocessing methods further validated with 3 000 spectra (Figs. 3c, f, S10) show a similar pattern as the trial with 100 spectra. In the scattering diagrams, spots with different colors show the preprocessing effect of various methods. Their positions relate to the values of RMSE or ${L}_{{\infty }}$ before (X-axis) and after (Y-axis) spectral preprocessing (Figs. 3a–c, d–f). We summarized these results as mean ± standard deviations (SD) in histograms (Fig. 3b, e, S10, Table S2).

The trial result shows that Polynomial fitting improves spectral quality in RMSE and ${L}_{{\infty }}$ compared to raw data but is still not as good as other methods. Wavelet transform has a better spectral preprocessing effect and spectral fidelity than conventional deep-learning spectral preprocessing methods, such as Residual CNN^*^ and UNet-1D^*^, with significantly lower RMSE and ${L}_{{\infty }}$ values (*P* > 0.001). The U-shape structure of the UNet-1D model makes it better spectral feature extraction and reconstruction capabilities than Residual CNN, no matter whether using mathematical simulation (*P* > 0.01) or RSGAN-generated spectra as training dataset (*P* > 0.001). The RSBPCNN model demonstrates excellent denoising and baseline correction capacity when using either training dataset, indicating superior fitting ability. The proposed RSPSSL scheme (RSBPCNN^#^) shows a remarkable reduction of RMSE and ${L}_{{\infty }}$ values by 99%, 88%, 91%, 90%, and 97%, 60%, 71%, 62% when compared to Polynomial fitting (*n* = 100), Wavelet transform (*n* = 100), Residual CNN^*^ (*n* = 3 000), and UNet-1D^*^ (*n* = 3 000), respectively. Moreover, by using RSGAN-generated simulative spectra as a training dataset, the RMSE/${L}_{{\infty }}$ values of Residual CNN, UNet-1D, and RSBPCNN preprocessed spectra reduce 5%/21%, 50%/30%, and 85%/54%, respectively. We found that the improvement rises when the model fitting ability increases. With the advantage of high-quality training data, UNet-1D^#^ shows a better preprocessing effect than mathematical simulative spectra trained RSBPCNN^*^ with a 27%/16% reduction of RMSE/${L}_{{\infty }}$. This phenomenon further confirms the vital role of training dataset quality.

To visualize the performance of the above methods, a spectrum (original RMSE = 277.89) is randomly selected and compared for demonstration (Fig. 3g–o). In this result, we found that Polynomial fitting failed to recover the Raman peaks distorted by a cataclysmic signal (the cliffy region of the spectrum, Fig. 3g) produced by a multi-level grating lens switching. This phenomenon is complex to mimic by the mathematical fitting, so it fails to identify this mutation signal and generates a fake peak in that spectral region (Fig. 3h). Residual CNN^*^, UNet-1D^*^_,_ and RSBPCNN^*^ also fail to deal with this mutation signal and generate fake peaks after preprocessing due to the lack of actual spectra features in mathematical simulative training spectra (Fig. S2). With RSGAN-generated training spectra, UNet-1D^#^ and RSBPCNN^#^ show significantly increased preprocessing effect and spectra fidelity, but Residual CNN^#^ only shows a marginal improvement. This difference indicates that the spectral preprocessing model requires higher feature abstraction and reconstruction abilities to benefit from high-fidelity training spectra.

Wavelet transform performs better than other established methods with the advantage of automatic adaption to focus the details of a spectrum, so it has better Raman peaks recognition ability than Residual CNN^*/#^ and UNet-1D^*^. Unfortunately, the compromised denoising ability impedes its performance in the upcoming application trials (Fig. 3i). In Fig. 3o, the roseate curve showing differences between RSBPCNN^#^ and the ground truth is smooth and horizontal, indicating outstanding spectral fidelity of the proposed RSPSSL scheme.

### Contribution in complex analyte classification: Cancer diagnosis trial

Analyte classification is the most demanded Raman spectroscopy application category, including drug detection, residual pesticide identification, bacteria classification, etc^9,11,12,34^. Disease diagnosis is one of the worthy applications reported in the literature. However, it has yet to be used in clinics due to the difficulties in complex spectral preprocessing and analysis and the immaturity of high throughput systems. Since the diagnosis accuracy reflects spectral fidelity, we will evaluate the contribution of the RSPSSL scheme in cancer diagnostic accuracy using preprocessed spectra.

**Fig. 4:**
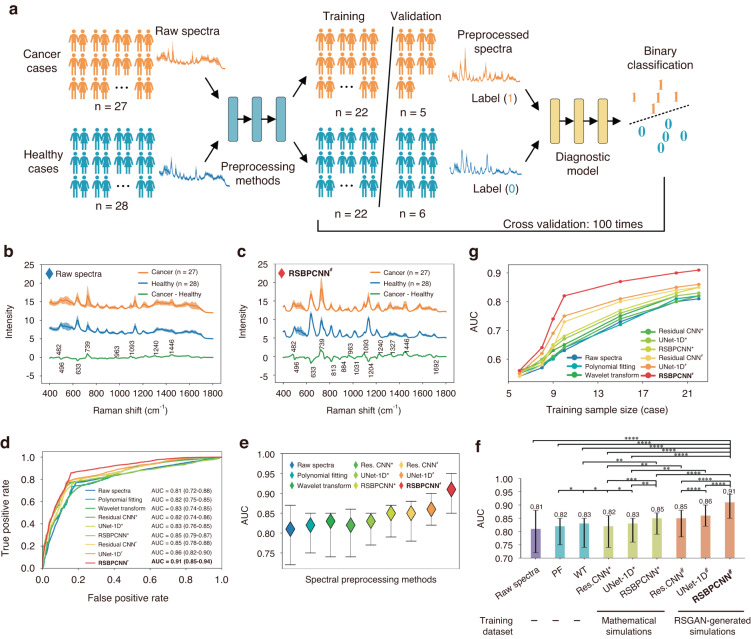
Cancer diagnosis trial with spectra preprocessed by different methods. **a** The trial workflow. **b** Curves and shadows show the normalized serum SERS spectra with mean ± SD collected from 27 Cancer (orange) and 28 healthy (blue) cases. The green curve is the subtraction of cancer and healthy serum spectra. **c** RSBPCNN^#^ preprocessed spectra of **b. d**–**f** Comparison of AUCs indicating cancer diagnosis accuracy of spectra preprocessed by different methods (training data size: *n* = 22 cancer and 22 healthy cases). **g** Cancer diagnostic accuracy of spectra preprocessed by different spectral preprocessing methods at different training sample sizes (case). Statistical significance was calculated using the Wilcoxon signed-rank test for two correlated samples. An asterisk * indicates *P* < 0.05, ** indicates *P* < 0.01, *** indicates *P* < 0.001, **** indicates *P* < 0.0001

This few-shot classification experiment classifies preprocessed spectra from 27 cancer serums and 28 healthy controls using a classification model, ResNet-1D (Fig. S8, S25). Twelve surface-enhanced Raman scattering (SERS) spectra for each serum sample were collected. The dataset proportion is training cases: validation cases = 8:2 with 100 times of cross validation (Fig. 4a). All spectra are preprocessed by the RSPSSL scheme (RSBPCNN^#^) (Fig. 4c) or other established preprocessing techniques (Fig. S7b–h) before input to the diagnostic model. The raw spectral group plays a role of control (Fig. 4a). The diagnosis accuracy matrices are indexes of integration areas under the receiver operating characteristic (ROC) curve (AUC), sensitivity, specificity, positive predictive value (PPV), and negative predictive value (NPV) (Fig. 4d–f, Table 1). After spectral preprocessing, nearly all the spectral baselines of cancer serum (orange) and healthy control serum (blue) are horizontal with reduced fluctuations but different richness and intensity of Raman peaks. The green curves showing the average subtraction result of these two kinds of serum spectra reflect their differences (Fig. 4b, c, S7). We found that RSBPCNN^#^ enhances the prominence of diagnostic featured spectral peaks. The intensity of peaks at 482 cm^−1^, 739 cm^−1^, 963 cm^−1^, 1093 cm^−1^, 1240 cm^−1^, and 1446 cm^−1^ was elevated in cancer spectra compared to Raw spectra (Fig. 4g). Since ideal spectra are inaccessible, the fidelity can only be reflected by diagnostic accuracy. From the result, we found that Polynomial fitting, Wavelet transform, Residual CNN^*^, and UNet-1D^*^ only improve the AUC by 1–2%, indicating unsatisfied spectral preprocessing fidelity (Table 1). Wavelet transform performs better than Polynomial fitting and Residual CNN^*^ but shows no significant difference with UNet-1D^*^ (Fig. 3, Table S2).

**Table 1:** Cancer diagnostic accuracy of spectra preprocessed by different spectral preprocessing methods

Preprocessing methods	Evaluation indexes				
	AUC	Sensitivity	Specificity	PPV	NPV
Raw spectra	0.81 (0.72–0.88)	77.08% (69.63–84.10%)	81.35% (73.71–87.58%)	78.03% (70.16–83.93%)	80.50% (71.37–84.72%)
Polynomial fitting	0.82 (0.75–0.85)	73.66% (68.17–78.63%)	83.36% (78.92–86.06%)	76.20% (72.18–82.53%)	81.40% (76.38–84.19%)
Wavelet transform	0.83 (0.74–0.85)	76.66% (71.83–80.31%)	85.90% (80.37–87.95%)	78.66% (73.67–81.73%)	84.45% (79.18–87.68%)
Residual CNN^*^	0.82 (0.74–0.86)	74.37% (67.32–77.53%)	81.60% (77.08–85.49%)	76.10% (73.25–80.14%)	80.17% (67.32–77.53%)
UNet-1D^*^	0.83 (0.76–0.85)	76.03% (72.98–79.63%)	83.27% (77.64–86.90%)	77.65% (72.09–82.87%)	81.96% (76.51–85.24%)
RSBPCNN^*^	0.85 (0.79–0.87)	78.30% (74.86–83.93%)	84.77% (81.85–88.32%)	79.57% (75.60–82.76%)	83.70% (79.09–86.88%)
Residual CNN^#^	0.85 (0.78–0.88)	77.69% (73.83–83.29%)	86.46% (82.49–89.37%)	79.62% (74.95–83.97%)	85.06% (82.97–88.14%)
UNet-1D^#^	0.86 (0.82–0.90)	79.01% (76.86–84.47%)	84.73% (80.72–87.90%)	80.21% (74.34–84.97%)	83.75% (79.67–86.49%)
RSBPCNN^#^	**0.91** (0.85–0.94)	**85.67%** (81.86–89.57%)	**87.53%** (84.65–90.97%)	**85.93%** (81.27–89.11%)	**87.29%** (85.31–90.03%)

Data are expressed by mean (95% CI). *AUC* area under the receiver operating characteristic (ROC) curve. *NPV* negative predictive value, *PPV* positive predictive value. ^*^: models are trained with mathematical simulation datasets. ^#^: models are trained with RSGAN-generated datasets. The highest mean value of each experimental group is highlighted in bold

Surprisingly, all the cancer diagnostic accuracy indexes of RSBPCNN^#^ treated spectra elevate dramatically with increases of 10% in AUC, 8.6% in sensitivity, 6.2% in specificity, 7.9% in PPV, and 6.8% in NPV compared to Raw spectra. It demonstrates great potential in translational medicine. The few-shot diagnostic accuracy is 0.91 in AUC (95% confidence interval (CI): 0.85–0.94), 85.67% in sensitivity, 87.53% in specificity, 85.93% in PPV, and 87.29% in NPV (Fig. 4c–e, Table 1, Fig. S11). The diagnostic accuracy will increase when the training data size goes up. The improvement of diagnostic accuracy by the RSPSSL scheme (RSBPCNN^#^) is ten times (ΔAUC) of Polynomial fitting and Residual CNN^*^, and five times (ΔAUC) of Wavelet transform and UNet-1D^*^. The RSGAN-generated training dataset contributes a 75% improvement (ΔAUC) of Residual CNN^#^ and a 60% improvement of UNet-1D^#^ and RSBPCNN^#^ in diagnostic accuracy. Although without a high-fidelity training dataset, RSBPCNN^*^ shows a 400% accuracy improvement than Polynomial fitting and Residual CNN^*^ and a 200% improvement than Wavelet transform and UNet-1D^*^ (Table 1). When the case number of the training dataset (training sample size) reduces, although the diagnostic accuracy of the RSBPCNN^#^ group declines, the advance of this work is more remarkable. The increased AUC of RSBPCNN^#^ is 8%, 8%, 11%, and 15% compared to the established preprocessing method UNet-1D* when the training sample size is 22, 20, 15, and 10 cases, respectively (Fig. 4g, Table S3). These results suggest excellent spectral preprocessing capacity and the high fidelity of the RSPSSL scheme in complex biomedical analytes.

### Performance in quantification: Paraquat concentration prediction

**Fig. 5:**
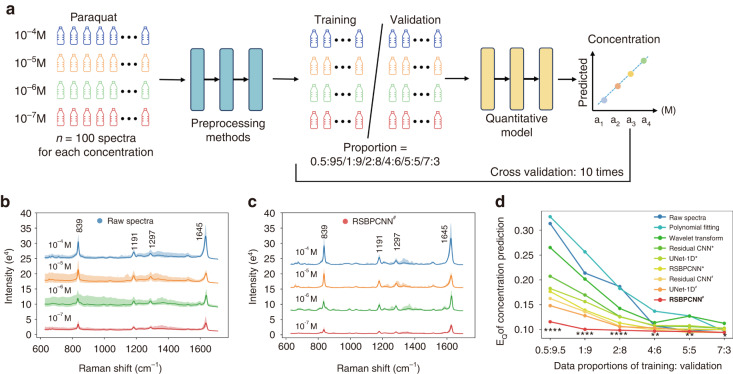
Paraquat concentration prediction trial using spectra preprocessed by different methods. **a** The trial workflow. **b** Curves and shadows showing the normalized SERS spectra with mean ± SD obtained from paraquat solution with different concentrations (10^−4^ M, 10^−5^ M, 10^−6^ M, and 10^−7^M). **c** Preprocessed spectra of **b** using RSBPCNN^#^. **d** Comparison of average paraquat concentration prediction error E_Q_ of spectra preprocessed by different methods at different training data proportions (*n* = 100 spectra for each concentration). Statistical significance was calculated using the Wilcoxon signed-rank test for two correlated samples between the superior established methods (UNet-1D^*^ for proportions of 0.5:9.5, 1:9, and 2:8; Residual CNN^*^ for proportions of 4:6, 5:5, and 7:3) and RSBPCNN^#^. An asterisk * indicates *P* < 0.05, ** indicates *P* < 0.01, *** indicates *P* < 0.001, **** indicates *P* < 0.0001

Another valuable application of Raman spectroscopy is concentration measurement, which relies on the slight changes in the featured Raman peak intensity of the analyte^11,35–38^. This kind of application has a higher demand for spectral fidelity, especially the intensity fidelity of the Raman peaks. In this trial, we included SERS spectra of paraquat solution with concentrations of 10^−4^ M, 10^−5^ M, 10^−6^ M, and 10^−7^ M (Fig. 5b) and 100 spectra for each concentration. Since the compositions of the paraquat solution are singular, the spectra are simply with only several Raman peaks. After being preprocessed with different methods, the spectra with the same concentration show identical features (Fig. S12). To further evaluate the slight differences among them, we conducted a concentration prediction trial at a series of training sample sizes (Fig. 5a, Fig. S13) with proportions of training and validation groups from 0.5:9.5 to 7:3. The quantitative model here is a continuous variable regression model with a basic structure of ResNet-1D (Fig. S9).

Spectra preprocessed by Polynomial fitting show increases in concentration prediction errors (E_Q_) compared to the Raw spectra group in most training data proportions, indicating unacceptable spectral preprocessing effect and fidelity. Wavelet transform, Residual CNN^*^, and UNet-1D^*^ preprocessed spectra also show increased prediction errors than the Raw spectra when the quantification training spectra are *n* ≥ 40 (for each concentration), suggesting unsatisfied spectral fidelity. However, when *n* ≤ 20, they show reduced prediction error of 7–24% in Wavelet transform, 21–34% in Residual CNN^*^, and 27–42% in UNet-1D^*^. This impressive result indicates that spectral fidelity is the predominant impact factor when the training data size is big enough to compensate for the disturbances of noises and baselines. When in few-shot applications, the impact of the denoising effect becomes critical to extract valuable features from the interference of noises and baselines, and the denoising effect is UNet-1D^*^ > Residual CNN^*^ > Wavelet transform in this trial.

In this singular component detection trail, Residual CNN^*^ shows a lower prediction error than Wavelet transform, and UNet-1D^*^ when *n* ≥ 40 indicates its higher spectral intensity fidelity in simple spectra preprocessing. RSGAN-generated spectra trained Residual CNN^#^ shows a consistent advance than UNet-1D^#^ when *n* ≥ 40. However, the balance changed when *n* ≤ 20, UNet-1D^*^ shows a better prediction accuracy than Residual CNN^*^ and Wavelet transform, indicating its better denoising capacity in simple spectral preprocessing. consistently, UNet-1D^#^ also shows advanced prediction accuracy than Residual CNN^#^ when *n* ≤ 20. Unlike complex analyte applications such as cancer diagnosis, deep learning-based spectral preprocessing methods perform better than Wavelet transform in simple spectral applications. However, the unsatisfied spectral fidelity of the established preprocessing methods still limits their robustness in analytical applications.

Although RSBPCNN^*^ preprocessed spectra show better prediction accuracy than the established methods, the improvement is marginal due to the simplicity of spectra. In contrast, the contribution of high-fidelity training spectra is remarkable. After training with RSGAN-generated data, Residual CNN^#^ and UNet-1D^#^ show significantly increased prediction accuracy in all training proportion groups, with the prediction errors reduction from 21–34% and 27–42% to 36–48% and 40–53% when *n* ≤ 20, and from (−8)–0% and (−10)–(−1)% to 4–7% and 1–6% when *n* ≥ 40. This result suggests the recovery of spectral fidelity after high-fidelity spectra training. Notably, the proposed RSPSSL scheme (RSBPCNN^#^) demonstrates a remarkably higher prediction accuracy than other preprocessing methods with error reduction from 35–44% to 47–63% when *n* ≤ 20 and from (−3)–5% to 2–10% when *n* ≥ 40, indicating excellent spectral preprocessing capacity, fidelity, and robustness in translational applications of quantification (Fig. 4, Table S4).

### Chemical resolution improvement through Raman hyperspectral image preprocessing

With chemical fingerprint information, label-free Raman spectral imaging is initiating a revolution in biomedical science and clinical applications^34,39,40^. New molecular mechanisms can be discovered by inspecting the spatial distribution of chemical bonds. Different from classification and quantification, any residual spectral noise and baseline signals will damage the pixel fidelity and chemical resolution of Raman hyperspectral images. The interpretation of Raman hyperspectral imaging demands a high-fidelity preprocessing technique.

**Fig. 6:**
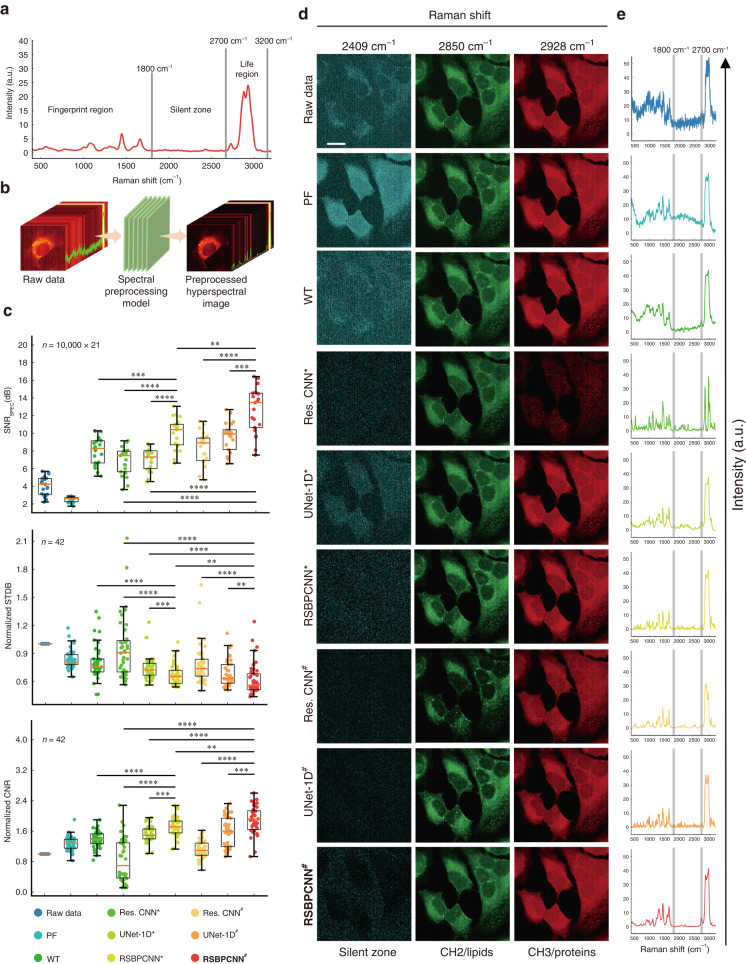
Raman hyperspectral image quality enhancement. **a** Regions demonstration of a full spectrum. **b** The workflow of the Raman hyperspectral image preprocessing trial. Samples include twelve images from the COS-7 cell line and nine from the Hela cell line with spectral integration times of 0.01, 0.05, and 0.1 s. **c** SNR_spec_: comparison of the average SNR of the top 10 000 spectra from the Raw data or hyperspectral images preprocessed by different methods. *n* = 10 000 spectra/image * 21 images. Comparison of normalized STDB and normalized CNR of the raw images and images preprocessed by different preprocessing methods. *n* = 21 lipid images (Raman shift of 2850 cm^−1^, CH_2_) + 21 protein/DNA images (Raman shift of 2928 cm^−1^, CH_3_). The central line, box, error bar, and dots indicate the median, inter-quartile range (Q1 and Q3), min-max range, and outliers, respectively. Statistical significance was assessed using the Mann-Whitney U Test for two independent samples and the Wilcoxon signed-rank test for two correlated samples. An asterisk * indicates *P* < 0.05, ** indicates *P* < 0.01, *** indicates *P* < 0.001, **** indicates *P* < 0.0001. **d** Visual inspection of spectral images of the Hela cell line with channels of the spectral silent zone, lipid (2850 cm^−1^), and proteins (2928 cm^−1^). The rows show the spectral channels, and the columns demonstrate different spectral preprocessing methods. **e** One randomly selected spectrum with an integration time of 0.1 s for visual inspection. Scale bar: 20 μm. PF: Polynomial fitting; WT: Wavelet transform; Res. CNN: Residual CNN

**Fig. 7:**
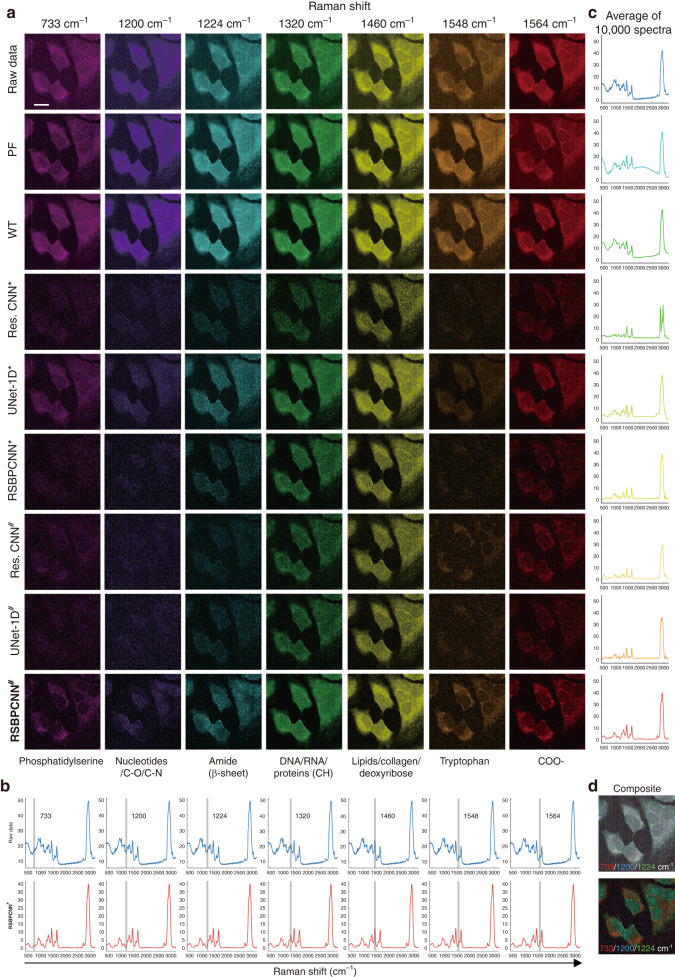
Chemical resolution recovery by the RSPSSL scheme. **a** Representative preprocessed Raman spectral image of the Hela cell line mapped with Raman shifts of the molecular fingerprint region. **b** Raman shift indication on the average spectra of the corresponding Raman spectral image. Each blue spectrum demonstrates the average of 10 000 spectra with top SNR_spec_ of the raw data image. Each red spectrum shows the average of 10 000 RSBPCNN^#^ preprocessed spectra with top SNR_spec_ from the corresponding hyperspectral image. **c** Average 10 000 spectra with top SNR_spec_ of each image preprocessed by different methods. **d** Merged images of the Raw data or RSBPCNN^#^ preprocessed spectra with Raman shifts of 733 cm^−1^ (red, phosphatidylserine), 1200 cm^−1^ (blue, nucleic acids & phosphates/C-O/C-N), and 1224 cm^−1^ (green, amide III (*β-*sheet structure)). Scale bar: 20 μm

In this trial, we captured nine hyperspectral images from the Hela cell line and twelve from the COS-7 cell line with spectral integration times of 0.01 s, 0.05 s, and 0.1 s, demonstrating various spectral qualities for robustness validation (Table S5). We calculated the image spectral signal-to-noise ratio (SNR_spec_) by averaging the top 10 000 spectra of 40 000 data for each image to avoid the involvement of background signals from the blank areas of coverslips. After spectral preprocessing, all the image SNR_spec_ increases except for Polynomial fitting (Fig. 6b, S16b, Table S6). When inspecting them, we found that Polynomial fitting preprocessed spectra had higher baselines than the Raw data, which introduced strong background signals to the images (Figs. 6, 7, S14–16, Movie S1, S2). Wavelet transform preprocessed spectra retain similar baselines as the Raw data, which damages their chemical resolution despite the SNR_spec_ being higher than Residual CNN^*^ and UNet-1D^*^. Residual CNN^*^ and UNet-1D^*^ show no significant difference in SNR_spec_ but with shortages of abnormal peak shape/intensity and high baselines, respectively (Figs. 6, 7, S14–16, Movie S1, S2). RSBPCNN^*^ shows a ≥50% improvement in SNR_spec_, indicating better denoising and baseline correction effects than the above-established preprocessing methods. The SNR_spec_ of Residual CNN, UNet-1D, and RSBPCNN increased by 1.5 dB, 2.5 dB, and 2.6 dB, with 52%, 83%, and 43% improvements after training with RSGAN-generated data (Fig. 6b). Their denoising effect also increased, especially the RSBPCNN^#^ model, showing clear Raman peaks and radically baseline elimination with flat Raman spectral silence zones (Fig S16b). The SNR_spec_ of RSBPCNN^#^ increases by 8.55 dB compared to Raw data and more than 4.58 dB to the established methods, which is a 212% improvement to Raw data and a 115% improvement to the established methods (Fig. 6b, Table S6). Moreover, the ratio of RSBPCNN^#^ preprocessed image SNR_spec_ to the superior established-method Wavelet transform increased from 155% to 169% when the integration times increased from 0.01 s to 0.4 s, suggesting the robustness of RSBPCNN^#^ (Fig S17, Table S7). This result primarily indicates the stability and fidelity of the denoising and baseline correction capacity of RSBPCNN^#^ when preprocessing high-throughput complex biomedical spectra with different qualities.

Except for spectral quality inspection, we evaluated the chemical resolution improvement by mapping all the spectral Raman shifts in hyperspectral images (Movie S1–S3). A biological Raman spectrum usually contains a fingerprint region (400–1800 cm^−1^) reflecting molecular chemical bond variations, a silent zone (1800–2700 cm^−1^) without any signals, and a life region (2700–3200 cm^−1^) showing superimposed Raman peaks of biomacromolecules^20,41^ (Fig. 6a). The images of the fingerprint region indicating chemical bond distribution are most informative but challenging to isolate from noise and baselines (Fig. 7, S16). The life region images play a role in morphology control, and the silent zone images are blank control. To quantify the image quality, we create life region images with Raman shifts of 2928 cm^−1^ and 2850 cm^−1^ (Fig. 6, S16). The spectral Raman shift of 2928 cm^−1^ corresponds to CH_3_ bond vibration dominating proteins/DNA showing cell profiles^12^ (Fig. 6b, S14b). The lipid channel images created by the Raman shift of 2850 cm^−1^ corresponding to the CH_2_ bond show the morphology of the cell membrane system and organelles such as endoplasmic reticulum, Golgi apparatus, and mitochondrion. Normalized SD of the background (STDB) and contrast-to-noise ratio (CNR) are image quality metrics due to a lack of ground truth (Fig. 6, S17–19). From the result, we found that the normalized CNR of images preprocessed by Polynomial fitting, Wavelet transform, and UNet-1D^*^ increased gradually from 28% to 52%, except for Residual CNN^*^. Residual CNN^*^ shows reduced normalized CNR with wide error bars due to unsatisfied fidelity, partially rescued by RSGAN-generated training spectra. RSBPCNN^*^ and RSBPCNN^#^ show improved normalized CNRs by 73% and 91% compared to raw data. Normalized STDB reflecting preprocessing stability is negatively related to image quality, which shows a consistent pattern among the above preprocessing methods. From the silent zone control spectral images of 2409 cm^−1^, we found that Polynomial fitting, Wavelet transform, and UNet-1D^*^ preprocessed images have strong background signals in the silent zones due to high baselines, indicating the incredibility of their fingerprint region images. RSGAN-generated training spectra further improve the background signal elimination effect of all deep preprocessing methods. With high-fidelity properties, RSBPCNN^#^ preprocessed images demonstrate more refined and continual details of subcellular structures, such as the tube structure of endoplasmic reticulum and silklike pseudopods. These results suggest the potential of RSBPCNN^#^ in chemical resolution improvement of the fingerprint region images.

**Fig. 8:**
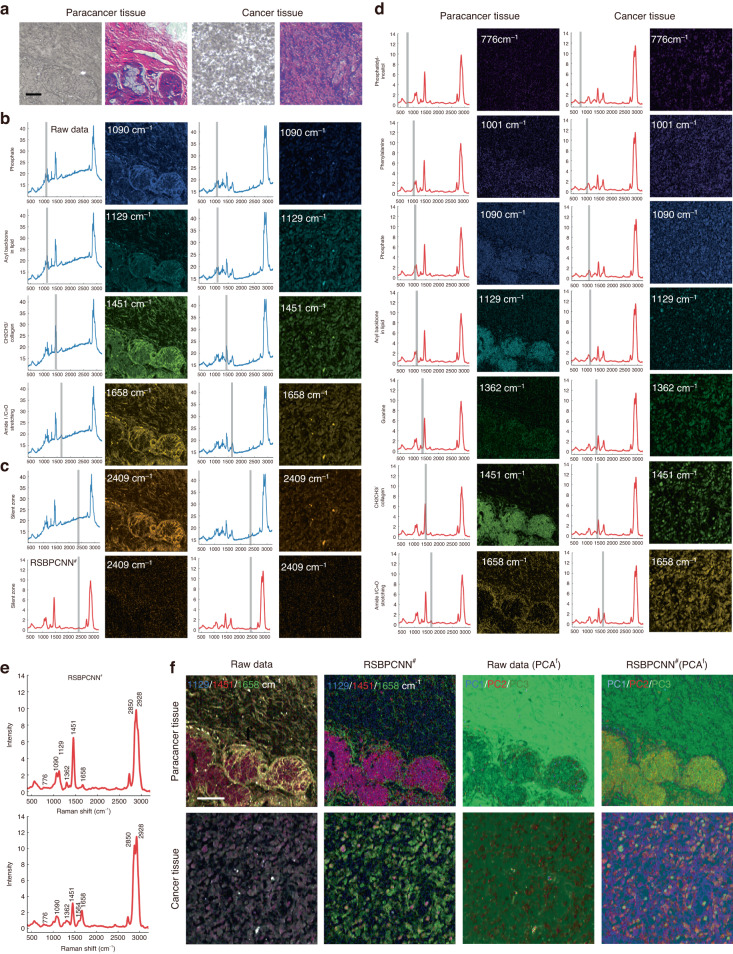
Chemical bond distribution in paracancer and cancer tissues. **a** Bright field and hematoxylin & eosin (H&E) staining images of paracancer and cancer (nasopharyngeal carcinoma) tissues. **b** Raman spectral image of Raman shifts at the fingerprint region of the Raw data. Left: Raman shift indications (gray line) on an averaged spectra of 10 000 data with top SNR_spec_ from the corresponding Raman hyperspectral image. Right: Raman spectral images with the indicated Raman shifts of paracancer and cancer tissues. **c** Comparison of the silent zone images of the Raw data (blue) and RSBPCNN^#^ preprocessed spectra (red). **d** Raman spectral images of the Raman shift at the fingerprint region of the RSBPCNN^#^ preprocessed spectra. Left: Raman shift indication (gray line) on average 10 000 RSBPCNN^#^ preprocessed spectra with top SNR_spec_. Right: Raman spectral images with representative Raman shifts from the spectral fingerprint region of paracancer or cancer tissues. **e** Demonstration of the representative Raman peaks of the averaged spectra of 10 000 RSBPCNN^#^ preprocessed spectra with top SNR_spec_ of paracancer or cancer tissues. **f** Merged images of Raman shifts 1129 cm^−1^ (blue), 1451 cm^−1^ (red), and 1658 cm^−1^ (green) of the Raw data or RSBPCNN^#^ preprocessed Raman spectral images of paracancer and cancer tissues. Merged images of the top three PCA features abstraction (PCA^f^) channels. Scale bar: 40 μm

Since Polynomial fitting, Wavelet transform, and UNet-1D^*^ fail to correct the spectral baselines and Residual CNN^*^ cannot restore accurate Raman peak shape and intensity, the analytical and scientific value of the spectral fingerprint region image is like a pearl covered by dust (Figs. 6–8, S14–16, Movie S1–S3). With the capacity of radical noise elimination and baseline correction, together with the high fidelity in Raman peak recovery and intensity maintenance, RSBPCNN^#^ preprocessed Raman spectral images show remarkable chemical-specific resolution (Figs. 7a, 8d, S20). Here, we demonstrated the chemical distribution images of the Hela cell line with fingerprint region Raman shifts of 733 cm^−1^, 1200 cm^−1^, 1224 cm^−1^, 1320 cm^−1^, 1460 cm^−1^, 1548 cm^−1^, 1564 cm^−1^ indicating the distributions of Phosphatidylserine, Nucleic acids & phosphates/C-O/C-N, Amide III (*β-*sheet structure), G(DNA/RNA)/CH deformation (proteins), Lipids/collagen/deoxyribose, Tryptophan, and COO− showing the correct location of related molecules (Fig. 7, S22, Movie S1)^42^. After combining channels of Raman shift of 733 cm^−1^, 1200 cm^−1^, and 1224 cm^−1^, the merged images show a complementary distribution of lipid organelles and nucleotides, which overlays with the distribution of protein with Amide III *β*-sheet structure indicating the cell profile (Fig. 7d). The COS-7 cell line shows similar results (Fig. S16, Movie S2).

Then, we further investigated this novel capacity of RSBPCNN^#^ in chemical resolution recovery of the spectral fingerprint region images of clinical tissue sections (Fig. 8, Movie S3). We found that the spontaneous Raman spectral images of cancer and paracancer tissues superimposed auto-fluorescence signals in all Raman shift channels, including the spectral silent zone, which concealed the actual signal of chemical bonds (Fig. 8b, c, Movie S3). The severe background signals make 1129 cm^−1^ (blue)/1451 cm^−1^ (red)/1658 cm^−1^ (green) merged picture show too much-unexpected overlay and lose the complementary distribution of 1658 cm^−1^ with the other two channels. The merged image of cancer tissue also demonstrates an unexpected overlay that leads to the gray color with the interference of noises and baselines. After spectral preprocessing using RSBPCNN^#^, the chemical bond signals are released from noises and baselines and recover their spatial information in hyperspectral images (Fig. 8c, d). The chemical bonds distribution with Raman shifts of 1129 cm^−1^ (*ν*(C-C) skeletal of acyl backbone in lipid (*trans* conformation)^43^) and 1451 cm^−1^ (CH_2_CH_3_ deformation (collagen assignment)^44^) are overlaid in most of the paracancer epithelial cells but partially overlaid in cancer tissue, indicating multiple cell types infiltration **(**Fig. 8e, Fig. S26). The distribution of chemical bonds of 1658 cm^−1^ (Amide I (*α*-helix)/collagen-like proteins^45^) changes from the gland surface of paracancer tissue into single-cell parcels in cancer tissues. The three-channel merged image of cancer tissue shows a scattering distribution of 1129 cm^−1^ and 1451 cm^−1^ double-positive cells (purple). We also found some diverse distribution of chemical bonds in tissues, such as phosphatidylinositol (776 cm^−1^), phosphate (1090 cm^−1^), guanine (1361 cm^−1^), and carbon particles (1590 cm^−1^), which can be further investigated in the future (Fig. 8d, Movie S3). In addition, we applied principal component analysis (PCA) on the hyperspectral images before or after spectral preprocessing for feature extraction. Surprisingly, the merged images of the top three PCA^f^ channels have consistent features with those chemical resolutions released by RSBPCNN^#^ with Raman shifts of 1129 cm^−1^, 1451 cm^−1^, and 1658 cm^−1^ (Fig. 8f, S21). This novel chemical-specific resolution recovery function of the high-fidelity RSPSSL scheme endows more potential for hyperspectral biomedical imaging in biomedical mechanism research and applications.

## Discussion

Here, we report a novel high-fidelity, robust, and universal Raman spectral preprocessing scheme RSPSSL for the prevalence of Raman spectroscopy, especially in spatial chemical bond omics profiling and biomedical mechanism screening. The broad applicability of the proposed RSPSSL scheme relied on the innovative construction of a self-supervised learning approach based on the spectral-physical composition relationship. The personalized design of the two-stage model has dramatically increased both the denoising and baseline correction capacity and the spectral fidelity. The first stage of the RSPSSL scheme self-constructs a high-fidelity spectra dataset with diverse signals of devices, samples, and spectral types. This RSGAN-generated dataset improves the preprocessing capacity and spectral fidelity of all the deep learning-based preprocessing methods. It contributes an 85% reduction in RMSE and a 54% reduction in ${L}_{{\infty }}$ values, a 60% improvement in the cancer diagnostic trial, a 71% (few-shot) and 52% improvement in paraquat concentration prediction, and a 30% improvement in SNR_spec_ to the RSBPCNN^*^ model (maximum = 100%). The RSGAN-generated training data also rescues some deficiencies of Residual CNN and UNet-1D. The second stage of the RSPSSL scheme (the preprocessing model RSBPCNN) demonstrates superior preprocessing effect and spectral fidelity than other methods, either trained by mathematical simulation or RSGAN-generated dataset, indicating improved fitting capacity.

These improvements break the barriers of cross-device, cross-sample, and cross-spectral type applications and enable spontaneous Raman entire spectral-based biomedical mechanism screening with label-free volumetric molecular images. Visualization of the chemical resolution in biomedical spontaneous Raman hyperspectral imaging requires excellent denoising capacity to improve the SNR, excellent baseline correction capacity to eliminate auto-fluorescence and recover the spectral intensity, and spectral fidelity to present the actual information. RSBPCNN^#^ unseals the Raman chemical-specific resolution, which enables observing the chemical bond distribution of the spectral fingerprint region in biomedical tissues. More disease-related multiplexed metabolic profiling can be visualized by Raman spectroscopy with this high-fidelity spectral preprocessing scheme in the future. Advanced spectral unmixing techniques are required to equip this unprecedented analytical imaging tool of Raman spectroscopy as an emerging spatial omics technique for mechanistic research and screening.

Despite the superior spectral fidelity demonstrated here, we have observed the presence of small spurious peaks in some spectra of the low SNR hyperspectral images (Fig. S14b). Although these peaks did not affect the chemical resolution due to the randomness of its appearance, we can improve it by involving spatial information instead of single spectra preprocessing. Nevertheless, as a proof of concept, the robustness and universality of RSBPCNN^#^ were validated by various applications using diverse sample types, data sizes, detection types, and devices. Its stability was also proved in the hyperspectral image preprocessing trial. In the future, more cross-device applications can be conducted to promote the translational application of the RSPSSL scheme in biomedical Raman spectroscopy. With minor modifications, the RSPSSL scheme is capable of background signal elimination and denoising of coherent Raman spectroscopy, such as the non-resonant background (NRB) in coherent anti-Stokes Raman scattering (CARS) and the cross-phase modulation (XPM) in stimulated Raman spectroscopy (SRS).

## Materials and methods

### Clinical samples

Clinical samples, data collection, and analysis in this research were performed under guidelines approved by the ethics committee of Southern University of Science and Technology (2022GZR048). All analyses were retrospective and in an anonymous manner, and the informed consent was waived by the local ethics and privacy committee.

### Materials

All the chemicals, like silver nitrate (AgNO_3_, Sigma-Aldrich), ascorbic acid (AA, Sigma-Aldrich), polyvinylpyrrolidone (PVP, Sigma-Aldrich), sodium citrate (Shanghai Macklin Biochemical Co., Ltd.), sodium chloride (NaCl, Shanghai Lingfeng Chemical Reagent Co., Ltd.), ammonia water (Shanghai Lingfeng Chemical Reagent Co., Ltd.), were analytical reagents without further purification. Ultrapure water (18 M\Omega·cm, 25 °C) was used for solution preparation.

### Primary spectral training datasets for the RSGAN model training

A total of 500 spectra from an open-accessed mineral dataset, named RRuff database, generated by the RRUFF project^46^ are randomly selected as one of the training datasets for the RSGAN model. The other 500 SERS spectra were domestically collected from 14 substances, including Glufosinate-ammonium, Glyphosate, Dipterex, Phosalone, Methamidophos, Parathion-methyl, Cartap, Orthene, Aquacide, Ethyl alcohol, Hexyl alcohol, Crystal violet 4-Aminothiophenol, and Rhodamine 6G. These spectra were collected by Raman spectroscopy (LabRAM HR Evolution, Horiba) with flower-like silver nanoparticles as SERS enhancement substrate. A typical synthesis procedure was applied^47^. In brief, we added 0.2 mL of 1 M AgNO_3_ solution and 2 mL of 1% PVP solution into a beaker with 10 mL ultrapure water. After mixing evenly on a thermostat magnetic stirrer at room temperature, 1 mL of 0.1 M AA solution was quickly added to the above-mixed solution and stirred for another 15 min. Finally, the reaction product was separated from the solution by centrifugation at 5 000 r/min for 10 min and then washed several times with ultrapure water to remove the impurities.

### Serum SERS detection

Serum samples come from 27 cancer patients and 28 normal healthy controls. They were detected using SERS with the enhancing substrates of silver nanoparticles (AgNPs). The spectra were collected by a Raman spectrometer (Portman785, Oceanhood, China) with an excitation wavelength of 785 nm, power of 50 mW, and integral time of 8 s per spectrum. Six spectra of each sample were collected randomly. The spectra with Raman shift ranging from 400 cm^−1^ to 1800 cm^−1^ were included in our analysis. AgNPs are synthesized through a seed growth method^48^, which can adjust the particle size. First, 50 µL of 0.1 M AA was added to a mixture that was boiled for five minutes, consisting of 47.5 mL boiling water, 1 mL of 1% sodium citrate, 1 mL of 1% AgNO_3,_ and 1.25 mL of 80 µM NaCl. Then, heat and stir the new mixture solution for one hour to ensure a complete reaction. The silver seed solution was produced. It was cooled to room temperature and stored at 4 °C. Then, the silver ammonia mixture was prepared by mixing 800 µL saturated ammonia water with 2 mL of 1% silver nitrate, then incubating for 10 min. To produce AgNP solution, 1 mL silver nano-seeds solution was used to react with 700 µL silver ammonia mixture, 20 mL 2.5 mM ascorbic acid, and 48.3 mL water successively. This solution was mixed evenly on a thermostat magnetic stirrer at room temperature for one hour. The AgNP solution was stored in the refrigerator at 4°C for later use.

### Paraquat solution SERS detection

Flower-like silver nanoparticles were used as SERS substrates. Raman signals were enhanced by mixing samples with flower-like nano-silver. SERS spectra of paraquat solution with concentrations of 10^–4^ M, 10^–5^ M, 10^–6^ M, and 10^–7^ M were collected by a Raman spectrometer (Renishaw Ina, USA) with an excitation wavelength of 785 nm, power of 50 mW, and integral time of 8 s. One hundred spectra were collected from each concentration. The Raman shifts ranging from 620–1800 cm^−1^ of the spectra were used in data analysis.

### Cell culture and tissue preparation

HeLa (Procell, CL-0101) and COS-7 (Procell, CL-0069) cells were obtained from Procell (Wuhan, China) and cultured in Dulbecco’s modified Eagle’s medium (DMEM, Procell) supplemented with 10% fetal bovine serum (Procell) in a humidified 5% CO_2_ atmosphere at 37 °C. For cell imaging, HeLa and COS-7 cells were seeded on glass slips placed in TC-treated 6-well plates (LabServ™, ThermoFisher) and culture for 24–48 h. Cells were washed twice with PBS and dried for image capture. Formalin-fixed paraffin-embedded tissues were dewaxed by dimethylbenzene, rehydrated in alcohol gradients, and dried at room temperature before hyperspectral imaging.

### Raman hyperspectral imaging

Raman hyperspectral images were captured by a confocal Raman microscope (WITec Alpha 300 R, UK) using an excitation laser with a wavelength of 532 nm. The laser beam was focused on tissue or cell samples by a 100X or 50X objective with a numerical aperture (NA) of 0.9 or 0.75. Forty thousand spectra per image were collected. The Raman shifts of each spectrum range from 400 cm^−1^ to 3200 cm^−1^. We activated the focus-tracking function for auto-focus during image acquisition.

### Accuracy metrics

In the experimental data preprocessing trial, the root mean squared error (RMSE) and the infinite norm (${L}_{{\infty }}$) are used to evaluate the spectral preprocessing effect and fidelity. Their functions are as follows,

5 $${RMSE}(x,y)=\sqrt{\frac{1}{n}{\sum }_{i}{({y}_{i}-{x}_{i})}^{2}}$$

6 $${L}_{{\infty }}(x,y)=\max {{\rm{|}}}_{n}[{y}_{i}-{x}_{i}]{\rm{|}}$$

Where $x$ and $y$ represent the preprocessed spectrum and ideal spectrum, respectively. $n$ represents data points of the spectrum.

In the cancer diagnosis trial, the accuracy metrics include area under the curve (AUC), sensitivity, specificity, negative predictive value (NPV), and positive predictive value (PPV). Their functions are as follows,

7 $${AUC}=\frac{\sum ({m}_{i},{n}_{j}{)}_{{m}_{i} > {n}_{j}}}{M* N}$$

8 $${Sensitivity}=\frac{{TP}}{{TP}+{FN}}$$

9 $${Specificity}=\frac{{TN}}{{TN}+{FP}}$$

10 $${PPV}=\frac{{TP}}{{TP}+{FP}}$$

11 $${NPV}=\frac{{TN}}{{TN}+{FN}}$$

The *TP, FN, FP*, and *TN* stand for true positive, false negative, false positive, and true negative, respectively. *M* represents the number of true positive samples, *N* represents the number of true negative samples, *m*_*i*_ represents the prediction score of true positive samples, and *n*_*j*_ represents the prediction score of true negative samples. All these indexes correlate positively to the diagnosis accuracy. Within these indexes, AUC is a comprehensive indicator for accuracy evaluation.

We use quantitative error (${E}_{Q}$) as an indicator in the paraquat concentration prediction trial. Its function is as follows,

12 $${E}_{Q}(x,y)=\sqrt{{\sum }_{n}^{i=1}(\log ({y}_{i})-\log {({x}_{i}))}^{2}}$$

Where $y$ is the actual concentration, $x$ is the predicted concentration from the quantitative model, and $n$ is the number of samples. The value of the quantitative error ${E}_{Q}$ correlates negatively to the prediction accuracy.

We use spectral signal-to-noise ratio (SNR_spec_), the normalized standard deviation of background (STDB), and contrast-to-noise ratio (CNR) to evaluate spectral quality, noise level, and contrast of images, respectively, in the Raman hyperspectral biomedical image preprocessing trial.

The function of SNR_spec_ is defined as follows,

13 $${SN}{R}_{{spec}}=10\mathrm{lg}\left(\frac{\frac{1}{s}{\sum }_{2800 < s < 3200}^{i}{I}_{s}}{\frac{1}{n}{\sum }_{1800 < n < 2800}^{i}{I}_{n}}\right)$$

Where ${I}_{s}$ represents the intensity of the C-H vibration region (2800–3200 cm^−1^), and ${I}_{n}$ represents the signal intensity of the silent region (1800–2800 cm^−1^). The silent region only contains noise signals in the spectra of label-free cells, so SNR_spec_ is the signal-to-noise ratio of the spectra.

The normalized STDB and normalized CNR are defined as follows,

14 $${CNR}=({C}_{{sig}}-{C}_{{bg}})/{\sigma }_{{bg}}$$

15 $${STDB}={{\sigma }_{{bg}}}^{p}$$

16 $${Normalized}\,{STDB}=\frac{{{\sigma }_{{bg}}}^{r}}{{{\sigma }_{{bg}}}^{p}}$$

17 $${Normalized}\,\text{CNR}=\frac{{CN}{R}^{r}}{{CN}{R}^{p}}$$

Where ${C}_{{sig}}$ and ${C}_{{bg}}$ are the means of the signal and background. ${\sigma }_{{bg}}$ is STDB. The ${{\sigma }_{{bg}}}^{r}$ and ${{\sigma }_{{bg}}}^{p}$ are STDB of the raw and preprocessed data. The ${{CNR}}^{r}$ and ${{CNR}}^{p}$ are CNR of the raw and preprocessed data. In this metric, we need to tag the regions of the sample and background in images. We implemented these evaluation processes with Python 3.11.2.

### Established spectral preprocessing methods

Polynomial fitting uses a numpy 1.23.5 built-in function to obtain polynomial coefficients and reconstruct baselines. They were followed by the error calculation with the original spectra after baseline corrections. When the iteration error was less than 5%, we terminated the iteration. The denoising process is with a similar process and calculation window of 50 pixels.

**Wavelet transform** is based on implementing the PyWavelets 1.4.1 with eight-layer decomposition using the wavelet base of “dB8” and then selects three to six layers as spectral signals for reconstruction. We run the iteration cycle 100 times to achieve a better denoising and baseline correction effect.

We provided the model structures and training processes of the Residual CNN^*/#^ and UNet-1D^*/#^ models in supplementary information (Fig. S23, S24).

### Statistics and reproducibility

Other than specially stated, quantitative data are presented as box-and-whisker plots with a center line demonstrating the median, limits indicating 75% and 25%, whiskers showing maximum and minimum, or bar charts with mean ± SD. The AUC of the 100 times cross validation results are expressed as a mean with 95% confidence intervals. We used the Wilcoxon signed-rank test for correlated samples to calculate the statistical significance between two experimental groups in the experimental data preprocessing trial, cancer diagnosis trial, paraquat concentration prediction trial, hyperspectral image STDB, and CNR. We performed the Mann-Whitney U test for independent samples to calculate the statistical difference between two experimental groups for the SNR_spec_ of the Raman hyperspectral cell image. Type I error correction has been carried out between each pairwise comparison. All the statistical tests are implemented with Python 3.11.2 (scipy 1.10.1). Statistical significance at *P* < 0.05, 0.01, 0.001, and 0.0001 are denoted by *, **, ***, and ****, respectively.

### Supplementary information

Supplementary Information for RSPSSL A Novel High-fidelity Raman Spectral Preprocessing Scheme to Enhance Biomedical Applications and Chemical Resolution Visualization

Movie S1. Raman shift screening of Hela cell line hyperspectral image before or after spectral preprocessing using different techniques

Movie S2. Raman shift screening of COS-7 cell line hyperspectral image before or after spectral preprocessing using different techniques

Movie S3. Raman shift screening of cancer (nasopharyngeal carcinoma) or paracancer tissue hyperspectral image before or after spectral preprocessing using RSBPCNN#

## Data Availability

The datasets generated and analyzed during this study are available in the GitHub repository. Source data are provided in this paper.

## Code availability

Code for the modeling work is available from the corresponding authors upon request. The link to use the proposed Raman spectral preprocessing scheme RSPSSL with sample data for the demo is available at https://github.com/oilab-sustech/RSPSSL.
