# Supplementary material for: RSPSSL: A novel high-fidelity Raman spectral preprocessing scheme to enhance biomedical applications and chemical resolution visualization
Source: Light Sci Appl. 2024 Feb 20;13:52. doi: 10.1038/s41377-024-01394-5 (PMC10876988; doi:10.1038/s41377-024-01394-5)
Supplement: Supplementary file 1 — Supplementary Information for RSPSSL A Novel High-fidelity Raman Spectral Preprocessing Scheme to Enhance Biomedical Applications and Chemical Resolution Visualization [file 41377_2024_1394_MOESM1_ESM.docx]

**Supplementary Information**

**for**

**RSPSSL: A Novel High-fidelity Raman Spectral Preprocessing Scheme to Enhance Biomedical Applications and Chemical Resolution Visualization**

Jiaqi Hu^#^, Gina Jinna Chen^#^*, Chenlong Xue, Pei Liang, Yanqun Xiang, Chuanlun Zhang, Xiaokeng Chi, Guoying Liu, Yanfang Ye, Dongyu Cui, De Zhang, Xiaojun yu, Hong Dang, Wen Zhang, Junfan Chen, Quan Tang, Penglai Guo, Ho-Pui Ho, Yuchao Li, Longqing Cong, Perry Ping Shum*

Contents

[Supplementary information of the RSPSSL scheme 3](#_Toc149666173)

[The cancer diagnostic model and the paraquat quantitative model 4](#_Toc149666174)

[Details of the training process of the deep learning-based preprocessing models 5](#_Toc149666175)

[Description of serum SERS peaks 5](#_Toc149666176)

[Supplementary Figures 6](#_Toc149666177)

[Supplementary Tables 28](#_Toc149666178)

# Supplementary information of the RSPSSL scheme

**The RSGAN model**

In fact, the noise and baseline extracted by wavelet transform from actual Raman spectra are different from the random Gaussian signal mimic noise and the Polynomial generated simulative baseline (Fig. S2). The actual baseline is more complex than the mathematical simulation. Previous simulative spectrum generation fails to fit the real Raman spectrum.

The critical point of the RSGAN model is the design of the loss function, which is^1^,

$$\min_{G}\max_{D}L(D,G)=E_{x_{1}\sim P_{d}(.)}\log D(x_{1})+E_{(x_{2},z)\sim P_{g}(.)}\log[1-D(G(x_{2},z))]$$

where $E_{x_{1}\sim P_{d}(.)}$ and$E_{(x_{2},z)\sim P_{g}(.)}$ represent the probability of whether the input is distinguished as a reference spectrum${(x}_{1})$ or a simulative spectrum $(G(x_{2},z))$. The simulative spectrum is produced by the generator$(G)$ by the combination of random Gaussian signals $(z)$ and the synthesized ideal spectra ${(x}_{2})$.

The loss function of RSGAN consists of two parts, including $\max_{D}L(D, G)$ and $\min_{G}L(D,G)$. They represent the training objectives for the discriminator $(D)$ and the generator $(G)$, respectively. The task of the discriminator $(D)$ is to identify simulative spectra synthesized and inputted by the generator $(G)$ and the reference spectrum ${(x}_{1})$ generated by the second step. The value of $\log(1-D(G(x_{2},z)))$ or $\log D(x_{1})$ is positively correlated to the distinguishing ability of the discriminator. The discriminator $(D)$ training is to push the value of $\max_{D}L(D,G)$ to the maximum. In another aspect, $\min_{G}L(D,G)$ achieves minimum means that the generator (G) generates a spectrum that conforms to the characteristics of the reference spectrum, and the discriminator $(D)$ fails to identify. In this situation, the value of $\log(1-D(G(x_{2},z)))$ and $\log D(x_{1})$ will be minimized. Within the iteration cycles, if the discriminator $(D)$ successfully recognizes the simulated spectrum, the generator $(G)$ will update to generate a more reliable spectrum; if the discriminator$(D$) fails to identify the spectrum, then it will be updated to increase the performance of classification. After training, this adversarial network reaches a balance where the generator $(G)$ produces high-fidelity simulative spectra, which the discriminator $(D)$ cannot recognize even after iteration.

After the training is completed, the ideal spectrum and the random Gaussian signal are input to the generator to generate the RSGAN-simulated spectrum. The generator generated the spectrum with different background signals by varying the random Gaussian noise (Fig. S4). The Raman peaks of these spectra are the same, with differences in baseline and noise.

**The RSBPCNN model**

The RSBPCNN model includes submodules of Raman-Patches, Pre-Encoder, Background Estimation, Downsampling, and Upsampling. Raman Patches uses a sliding window algorithm to segment the spectrum into different partitions to ensure optimal results (Fig.S5). Raman-Patches has two key parameters: Stride and Size. The size indicates the length of the window, which determines the size of a patch at the time of data input. The stride indicates the step size of the sliding window. This ensures that the Raman peaks at the edges of the dropping window are still handled well by sliding at a certain step size. In addition, the Downsampling and Upsampling are structured in detail (Fig. S6), mainly using Conv1d and ConvTranspose1d, with different structures at different depths of the Downsampling to extract different features. Then the features are restored to the spectrum using Upsampling.

# The cancer diagnostic model and the paraquat quantitative model

**Details of the diagnostic model and its training process**

The diagnostic model here is a binary model with a basic structure of ResNet-1D (Figure S8). In general, the diagnostic model learns the diagnostic features from the input training spectra and links them to their clinical diagnosis result (label) during the model training process by the feedback of a loss function. After training, the parameters of the diagnostic model are optimized by this data-driven approach, which makes the diagnostic model obtain the diagnostic capacity. The quality of training data decides the diagnosis capacity of the trained diagnostic model. At this moment, we can validate its diagnosis capacity by using an independent validation dataset (case n = M+N) as input without a label, the output result of the diagnostic model is 1 (cancer case) or 0 (healthy case). When comparing this diagnostic result to the ground truth (clinical diagnosis), we can calculate the accuracy rate of this diagnostic model. In our experiment, we repeated these independent training and validation cycles 100 times. The results shown in the article are the average accuracy of this 100-times validation (Fig. 4a).

This model follows the ResNet architecture, in which the networks are composed of one feature extractor and two full connection layers (Figure S8). The feature extractor discerns cancer-diagnostic relevant features within the spectra, while two full connection layers subsequently translate these features into classification outcomes. The feature extractor consists of multiple convolutional layers forming a residual network. Each conv1d contains a convolution layer, batch normalization, and the rectified linear unit (ReLU) nonlinear activation, in which the batch normalization layer aims to speed up the training process. ReLU could provide nonlinearity in the network.

The loss function is the cross-entropy loss function. The update-optimizer uses adaptive moment estimation (Adam) to optimize with a learning rate of 10^-3^. The batch size of each training is 32, and the training period is 200 epochs. All models were trained using PyTorch (version 2.0.0) on a single NVIDIA RTX A6000 GPU with 48 GB of RAM. The learning rate is multiplied by 0.6 if validation loss does not improve for 20 epochs.

**Details of the quantitative model and its training process**

The quantitative model here is a continuous variable regression model with a basic structure of ResNet-1D (Figure S9). In general, the quantitative model learns the spectral features from the input training spectra and links them to their concentration result (label) during the model training process by the feedback of a loss function. After training, the parameters of the quantitative model are optimized by this data-driven approach, which makes the quantitative model obtain the concentration prediction capacity. The quality of training data decides the concentration prediction capacity of the trained quantitative model. At this moment, we can validate its concentration prediction capacity by using an independent validation dataset (case n) as input without label, the output result of the quantitative model is concentration. When comparing this prediction result to the ground truth (concentration), we can calculate the E_Q_ of this quantitative model. In our experiment, we repeated these independent training and validation cycles 10 times. The results shown in the article are the average E_Q_ of this 10-times validation (Fig. 5a).

This model follows the ResNet architecture, in which the networks are composed of one feature extractor and two full connection layers (Figure S9). The feature extractor discerns quantitative relevant features within the spectra, while two full connection layers subsequently translate these features into concentration outcomes. The feature extractor consists of multiple convolutional layers forming a residual network. Each conv1d contains a convolution layer, batch normalization, and the rectified linear unit (ReLU) nonlinear activation, in which the batch normalization layer aims to speed up the training process. ReLU could provide nonlinearity in the network.

The loss function was selected as the Huber loss function. The update-optimizer uses adaptive moment estimation (Adam) to optimize with a learning rate of 10^-3^. The batch size of each training is 32, and the training period is 200 epochs. All models were trained using PyTorch (version 2.0.0) on a single NVIDIA RTX A6000 GPU with 48 GB of RAM. The learning rate is multiplied by 0.6 if validation loss does not improve for 20 epochs.

# Details of the training process of the deep learning-based preprocessing models

**Residual CNN^*/#^**, **UNet-1D^*/#^**, and **RSBPCNN^*/#^**

The models trained by mathematical simulative spectra are marked as ^*^ or those trained by RSGAN-generated spectral data are marked as ^#^. The models use raw spectra with noise and baseline as inputs and ideal spectra as outputs. In these models, their loss functions are the same: Huber loss function. The update-optimizer uses adaptive moment estimation (Adam) to optimize with a learning rate of 10^-4^. The batch size of each training is 64, and the training period is 300 epochs. All models were trained using PyTorch (version 2.0.0) on a single NVIDIA RTX A6000 GPU with 48 GB of RAM. The learning rate is multiplied by 0.7 if validation loss does not improve for 20 epochs.

**Description of serum SERS peaks**

We found that RSBPCNN^#^ enhances the prominence of key spectral peaks beneficial for diagnosis (Table S11, Fig. 4). The intensity of peaks at 482 cm^−1^, 739 cm^−1^, 963 cm^−1^, 1093 cm^−1^, 1240 cm^−1^, and 1446 cm^−1^ were elevated in cancer spectra compared to Raw spectra. The peaks of 496 cm^−1^ and 1327 cm^−1^ in the substrate curve of cancer to healthy control spectra become visible after RSBPCNN^#^ preprocessing. The decreasing intensity of 633 cm^−1^ Raman peak indicating phenylalanine was found after RSBPCNN^#^ spectral preprocessing. The decreasing Raman peaks of 813 cm^−1^, 884 cm^−1^, 1031 cm^−1^, 1131 cm^−1^, 1204 cm^−1^, 1240 cm^−1^, 1204 cm^−1^, and 1692 cm^−1^ appeared in the substrative curve of cancer compared to healthy control spectra after RSBPCNN^#^ preprocessing.

Moreover, several studies in the literature have shown that various biological constituents in serum samples, such as lipids, proteins, and nucleic acids, affect corresponding SERS signal intensity. Therefore, SERS peak analysis helped to explain the distinct serum component changes that were observed among the various cancerous groups. The mean normalized intensities in the cancer groups of the serum SERS bands at 482 cm^−1^ (DNA), 739 (DNA/Tryptophan), 963 cm^−1^ (Stretching C-O ribose), 1093 cm^−1^ (ν(C–N) d-Mannos), 1240 cm^−1^ (A, C, or T, ring stretching), 1327 cm^−1^ (DNA and phospholipids Collagen) and 1446 cm^−1^ (δ(CH2) lipids/protein) were found to be higher than the healthy control group. The SERS peak at 1446 cm^−1^ with higher intensity was previously regarded as a diagnostically significant cancer characteristic, (e.g., nasopharyngeal cancer and gastric cancer). In contrast, the serum SERS band intensities at 496 cm^−1^ (Ring vibration/Tryptophan), 633 cm^−1^ (L-tyrosine/C-S stretch), 813 cm^−1^ (Tyrosine), 884 cm^−1^ (δ(C-O-H)/glutathione/d-(C)-galactosamine), 1031 cm^−1^ (δ(C-H) Phenylalanine), 1131 cm^−1^ (C–N stretch/D-mannose), 1204 cm^−1^ (Tryptophan/phenylalanine), and 1692cm^−1^ (Citric Acid) appear higher in the healthy control group serum samples.

# Supplementary Figures


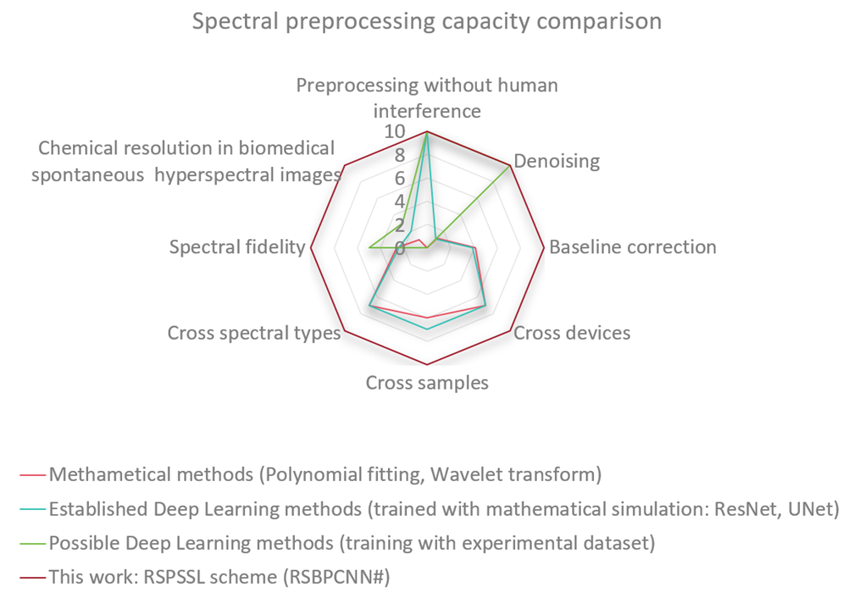


**Figure S1. Spectral preprocessing capacity comparison.**


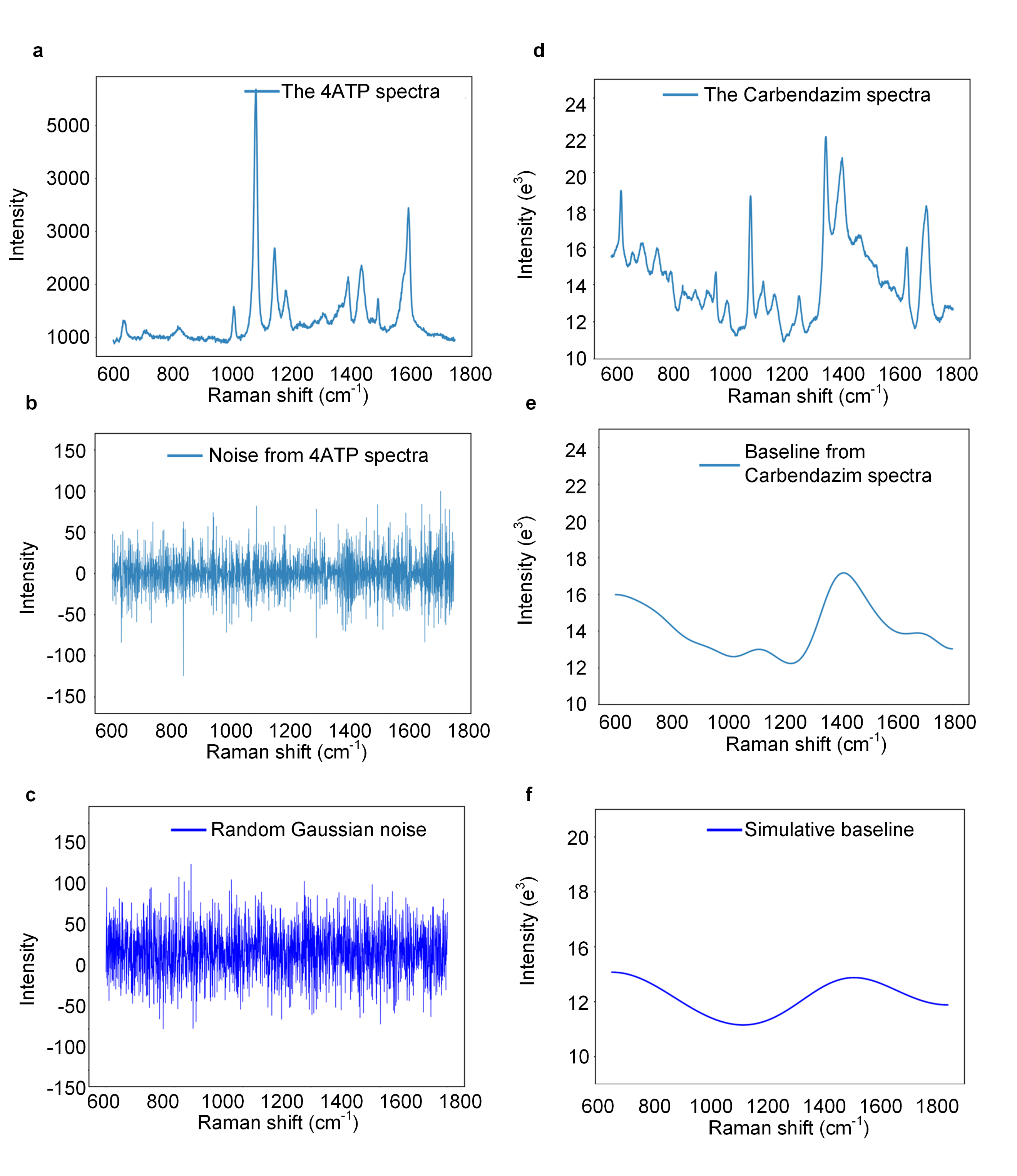


**Figure S2. Differences in noise/baseline signals between actual Raman spectra and mathematical simulations. (**a) A SERS spectrum of 4-aminothiophenol (4ATP). (b) Noise signals extracted from (a**)** through Wavelet transform. (c) Simulative noise signals using random Gaussian signals. (d) A SERS spectrum of carbendazim. (e) Baseline signal extracted from (d) through Wavelet transform. (f) Simulative baseline signal generated by Polynomial.


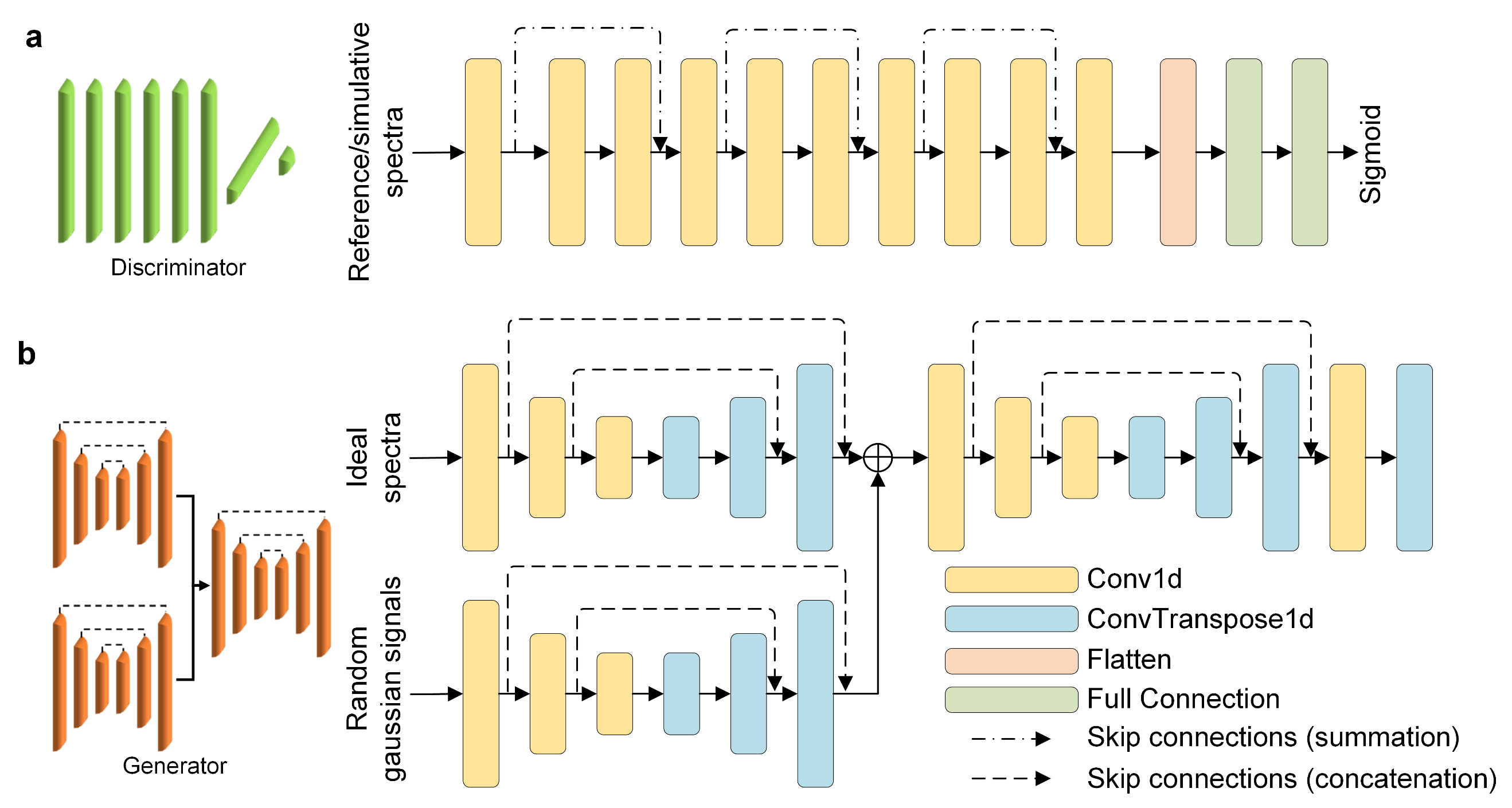


**Figure S3. The model structures of discriminator and generator blocks in the RSGAN model.** (a) Structure of the discriminator block. (b) Structure of the generator block.


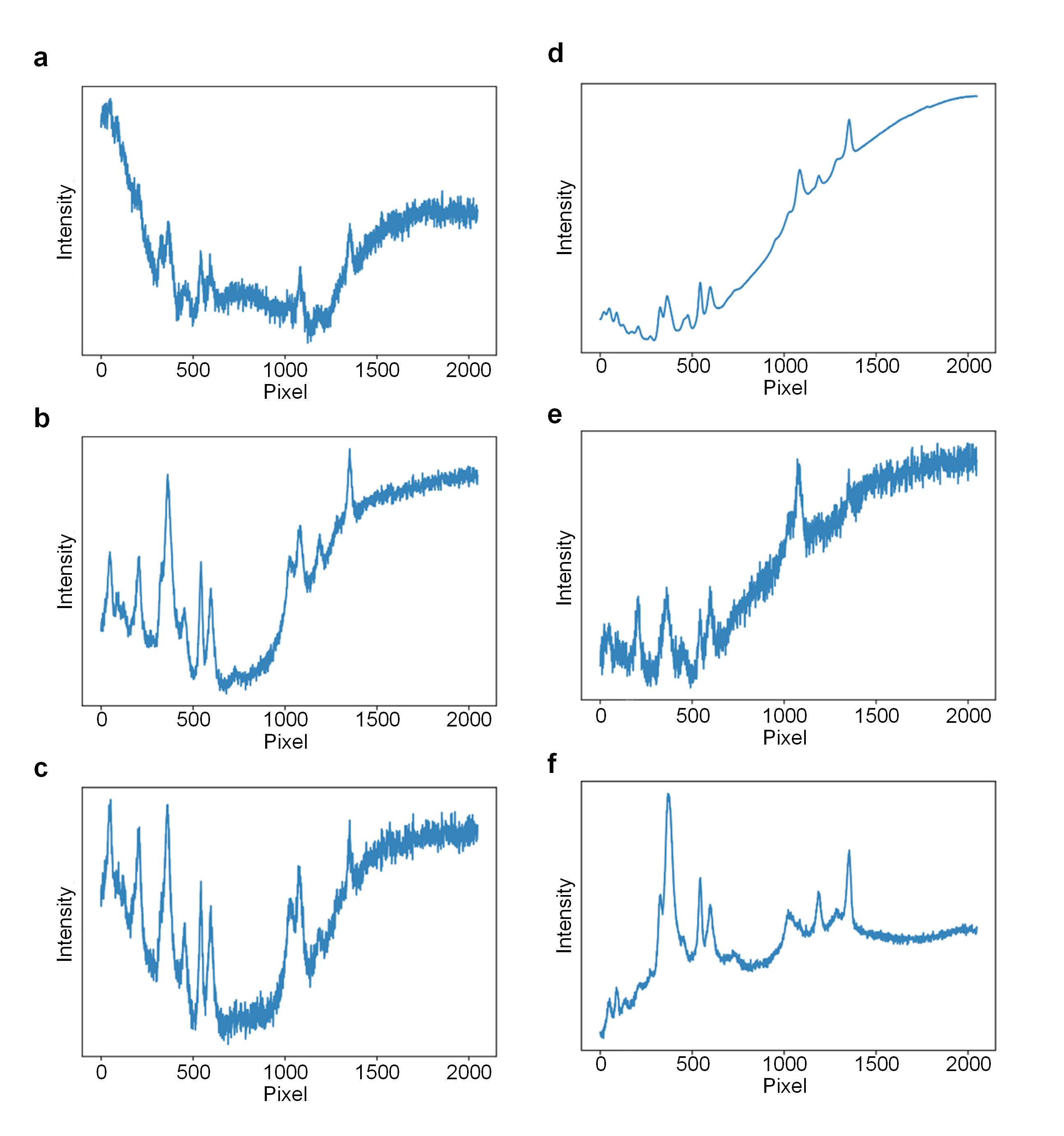


**Figure S4.** **Raman spectra with diverse noise and baseline signals generated by the RSGAN model based on one ideal spectrum.**


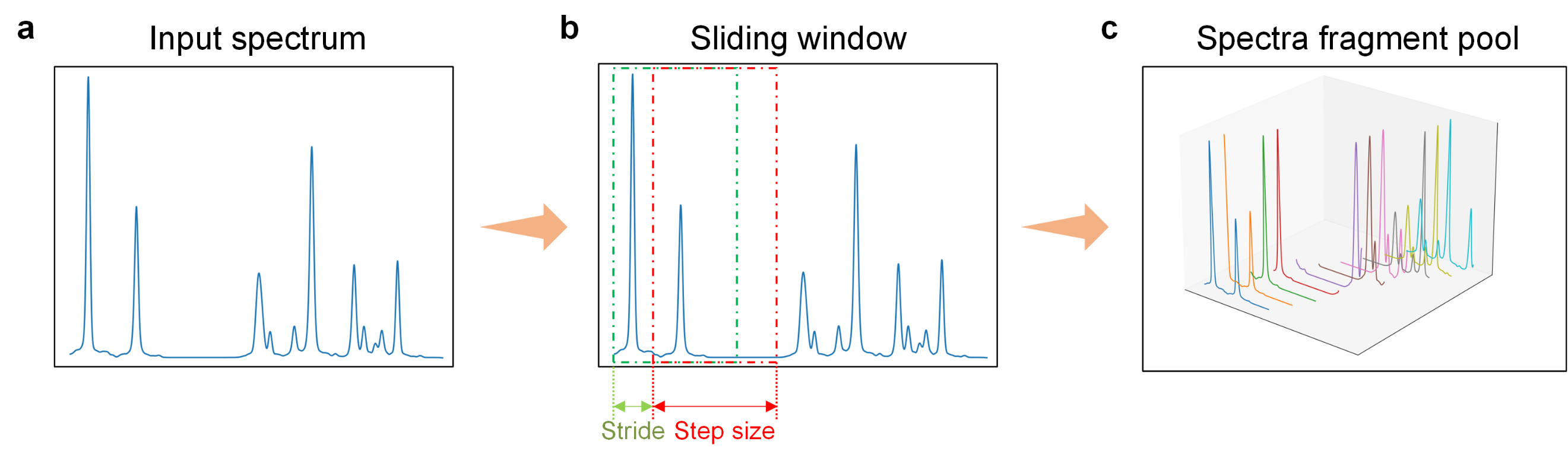


**Figure S5. Demonstration of the Sliding window segmentation strategy.**


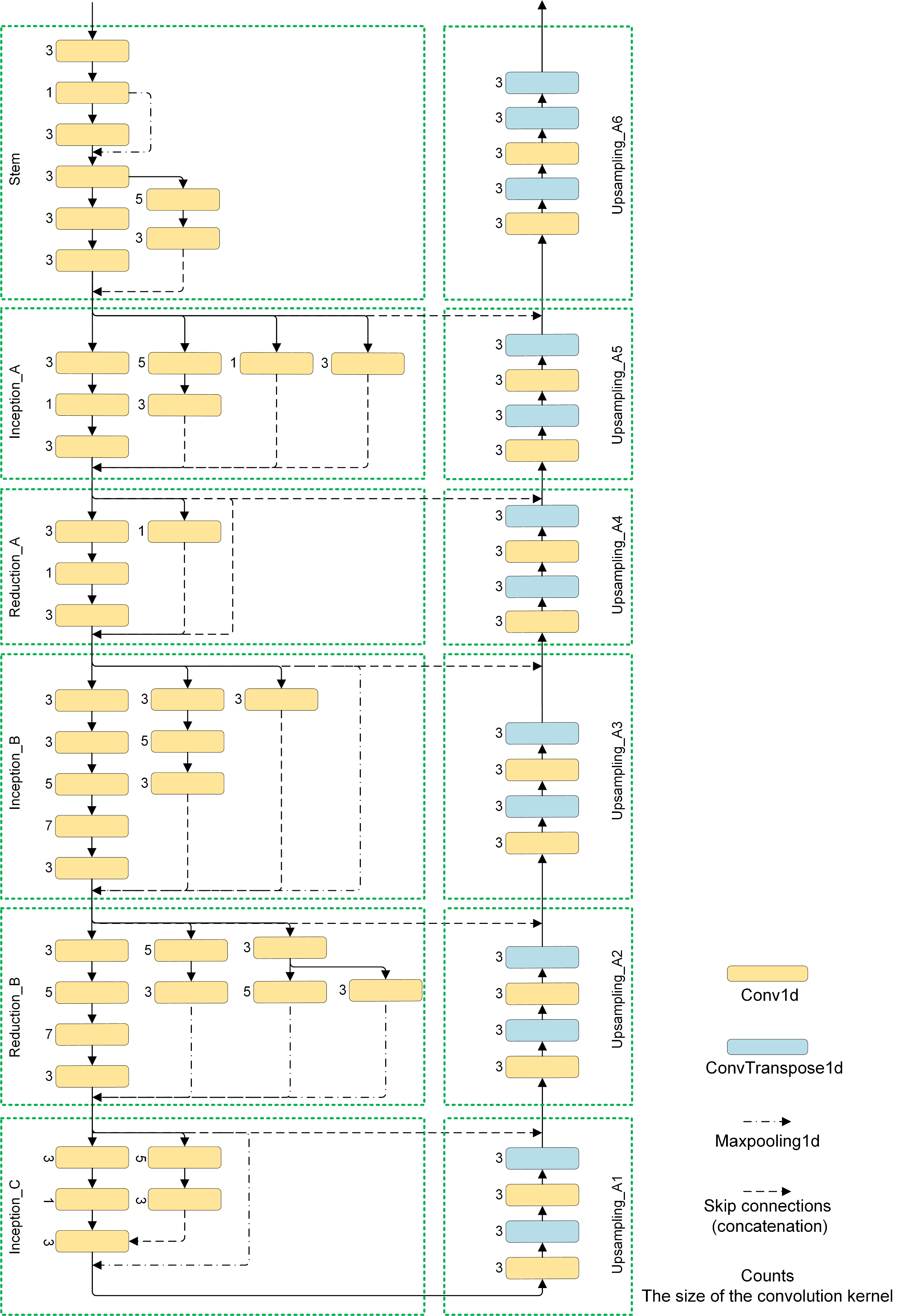
**Figure S6. Structure of the Downsampling and Upsampling submodules in the RSBPCNN model.**


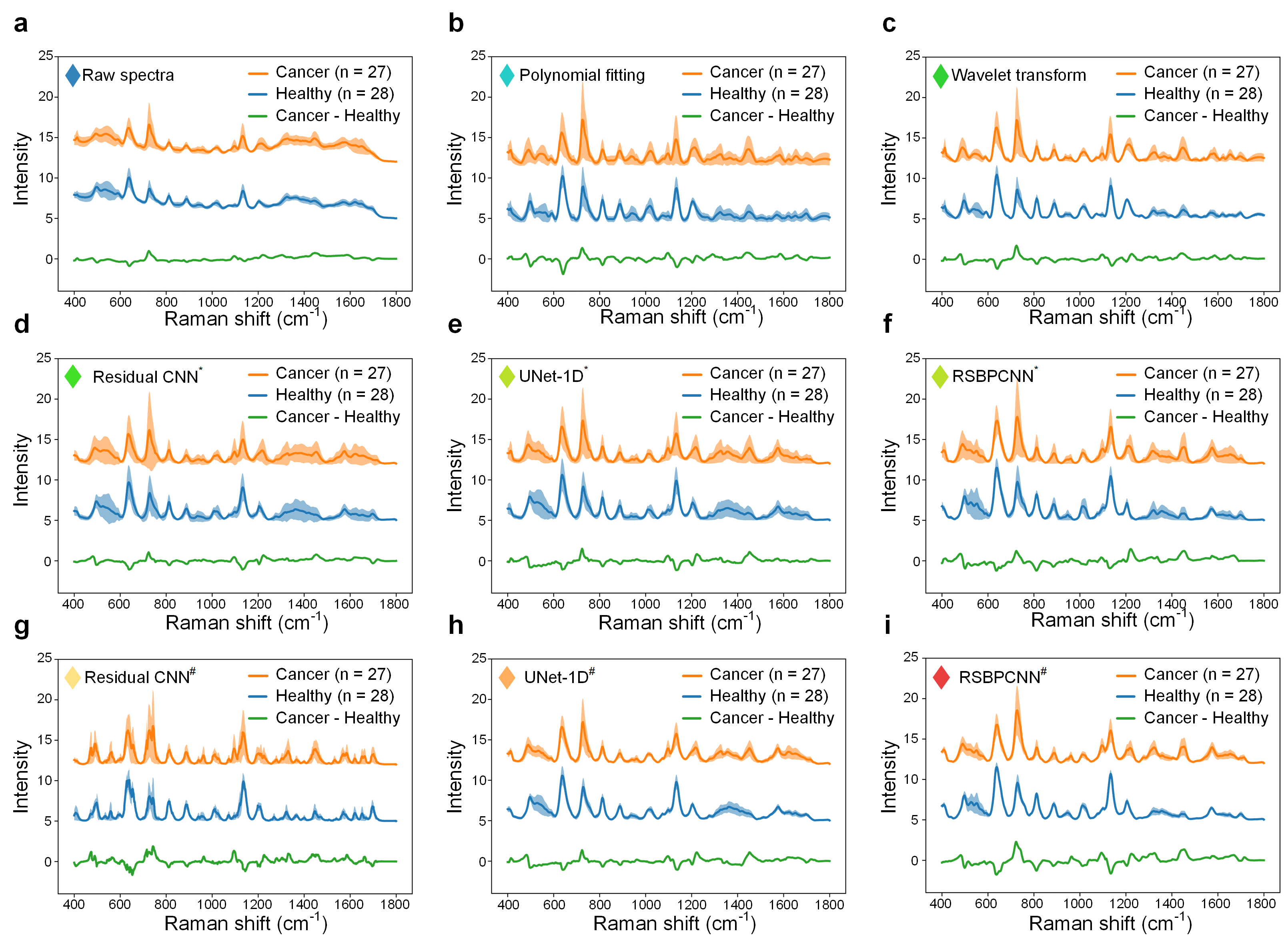


**Figure S7.** **Normalized SERS spectra used in the cancer diagnosis trial.** (a) Curves and shadows show the normalized serum SERS spectra with mean ± SD collected from cancer serum (n = 27 cases) or healthy controls (n = 28 cases). Twelve spectra for each case. (b) Spectra preprocessed by Polynomial fitting. (c) Spectra preprocessed by Wavelet transform. (d) Spectra preprocessed by Residual CNN^*^. (e) Spectra preprocessed by UNet-1D^*^. (f) Spectra preprocessed by RSBPCNN^*^. (g) Spectra preprocessed by Residual CNN^#^. (h) Spectra preprocessed by UNet-1D^#^. (i) Spectra preprocessed by RSBPCNN^#^. Cancer - Healthy (green curve): the subtraction of average cancerous serum spectra and average healthy control spectra. ^*^: The training dataset is mathematical simulation. ^#^: The training dataset is RSGAN-generated spectra.


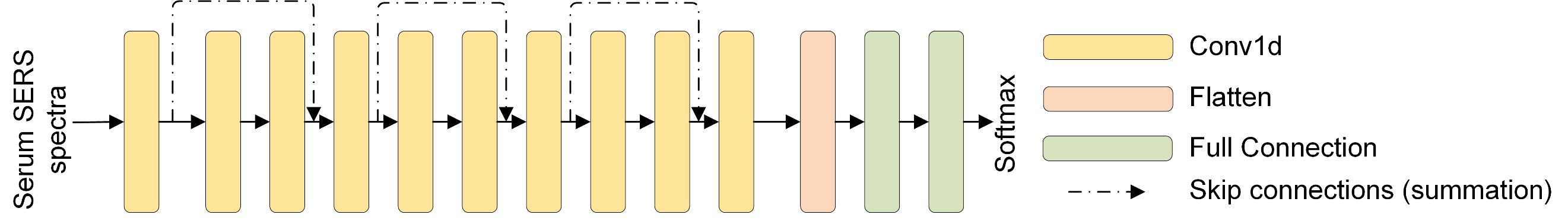
**Figure S8.** **Structure of the diagnostic model ResNet-1D used in cancer diagnosis trial (a binary model).**


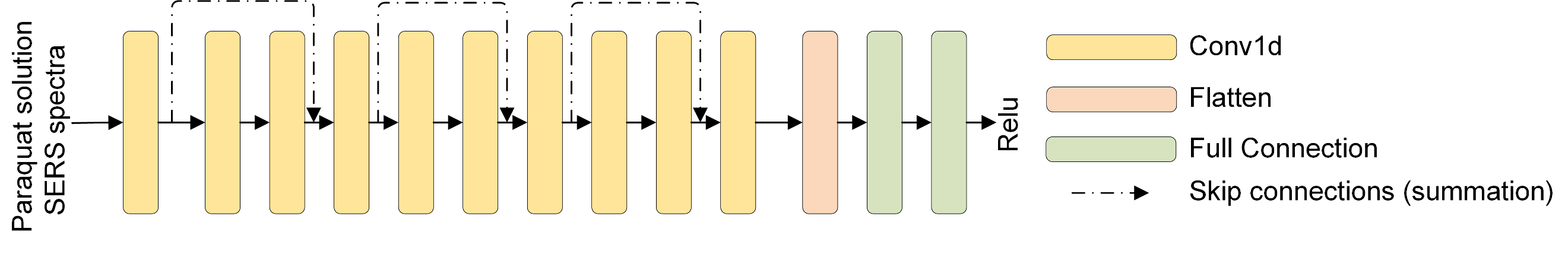
**Figure S9.** **Structure of the quantitative model ResNet-1D used in the SERS spectra-based paraquat concentration prediction trial (a continuous variable regression model).**


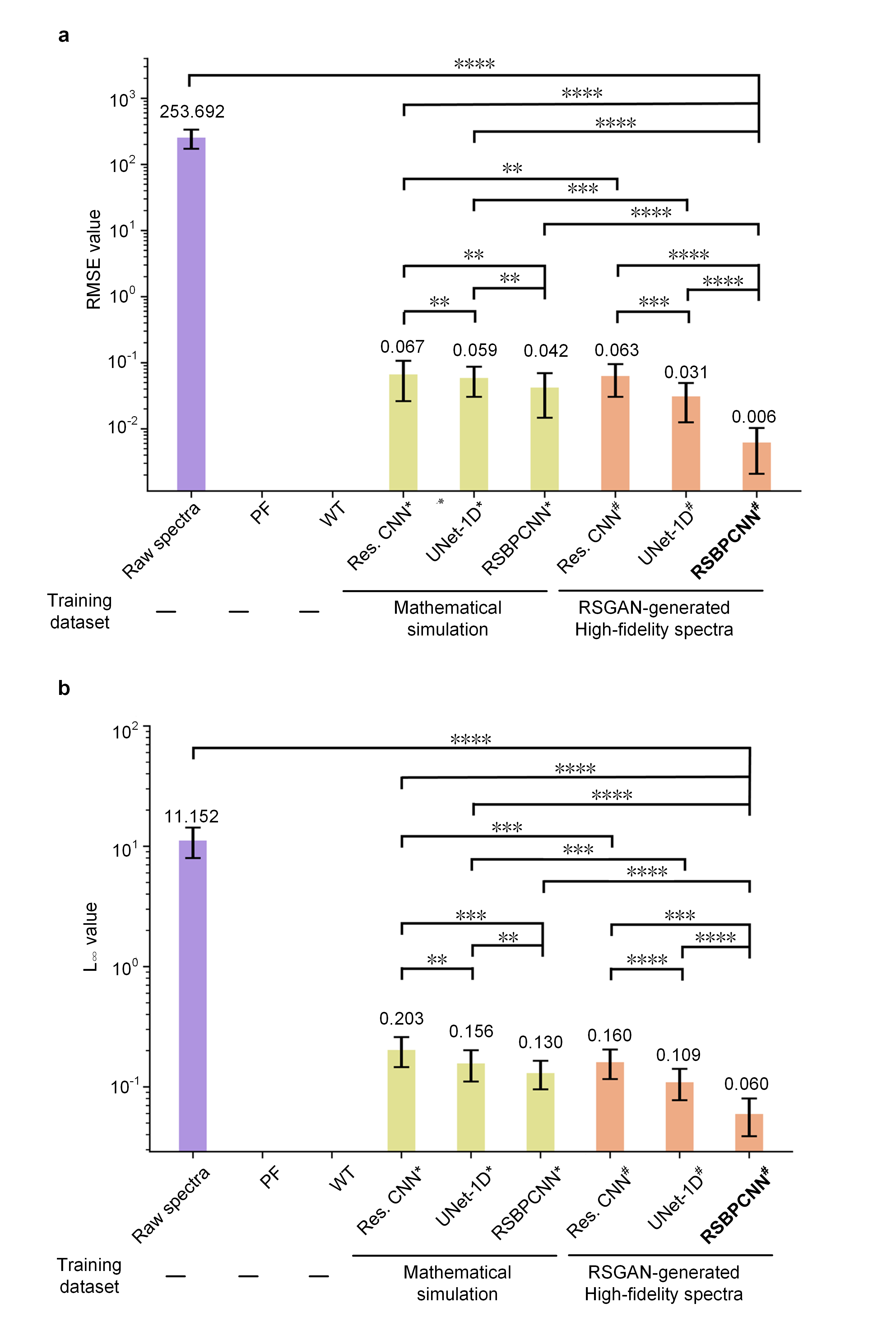


**Figure S10. Results of the experimental data preprocessing trial.** (a-b) Raman Spectral preprocessing effects of the proposed and established methods with independent spectral datasets (n = 3 000 spectra). Results are showed in histograms. Y-axis: The mean ± SD of the RMSE (a)/L_∞_ (b) values of the raw or preprocessed spectra; X-axis: Raw spectrum group and different deep learning-based spectral preprocessing method groups, including Residual CNN^*^, UNet-1D*, RSBPCNN^*^, Residual CNN^#^, UNet-1D^#^, and RSBPCNN^#^. Statistical significance was accessed using Wilcoxon signed-rank test for two correlated samples. * Represents *P* value < 0.05, ** represents *P* value < 0.01, *** represents *P* value < 0.001, **** represents *P* value < 0.0001. ^*^: indicates that the model was trained using mathematical simulation datasets. ^#^: indicates that the model was trained by RSGAN-generated spectral datasets. Res. CNN: Residual CNN.


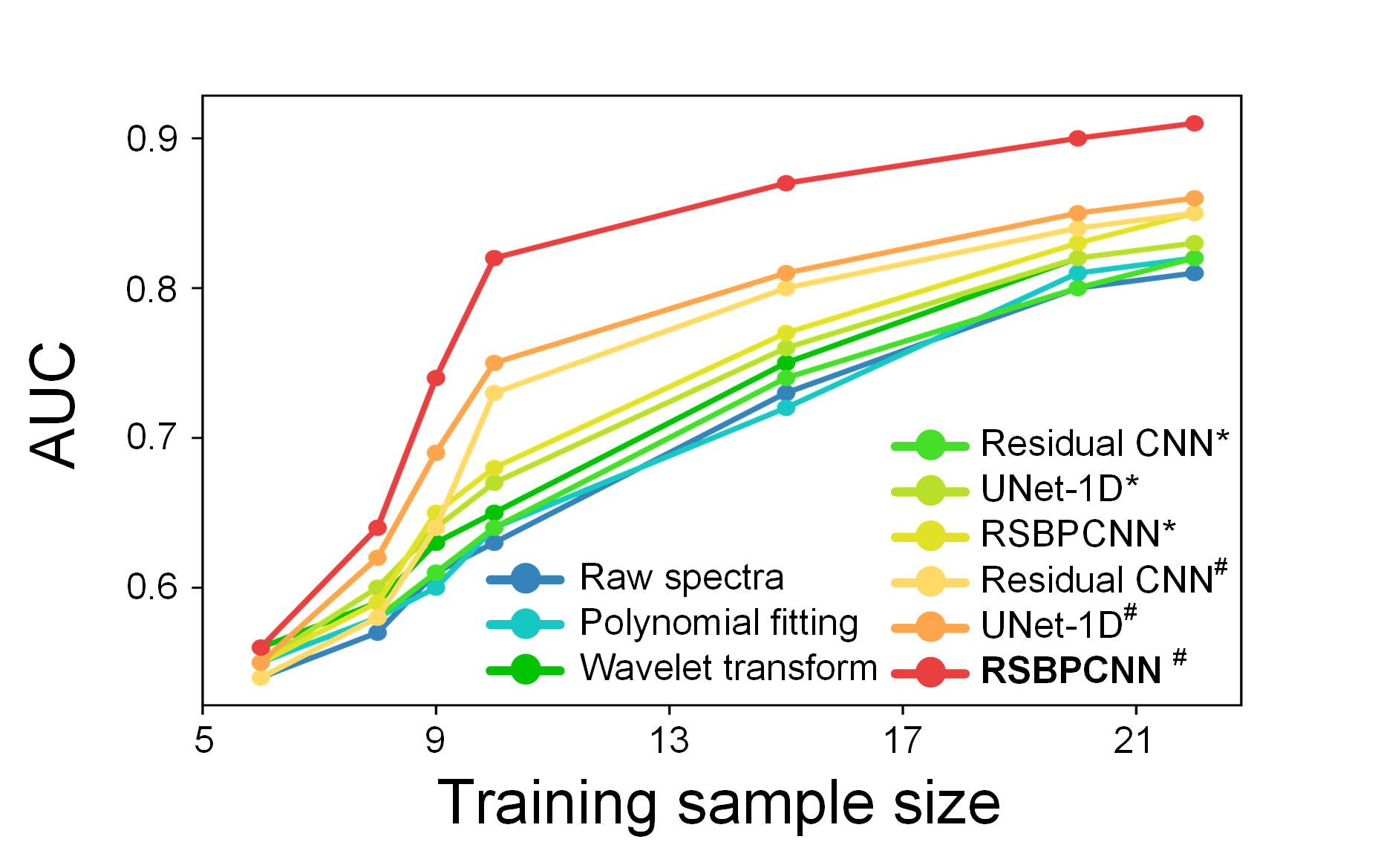


**Figure S11.** **Cancer diagnostic accuracy of spectra preprocessed by different spectral preprocessing methods at different training sample sizes (case)**.


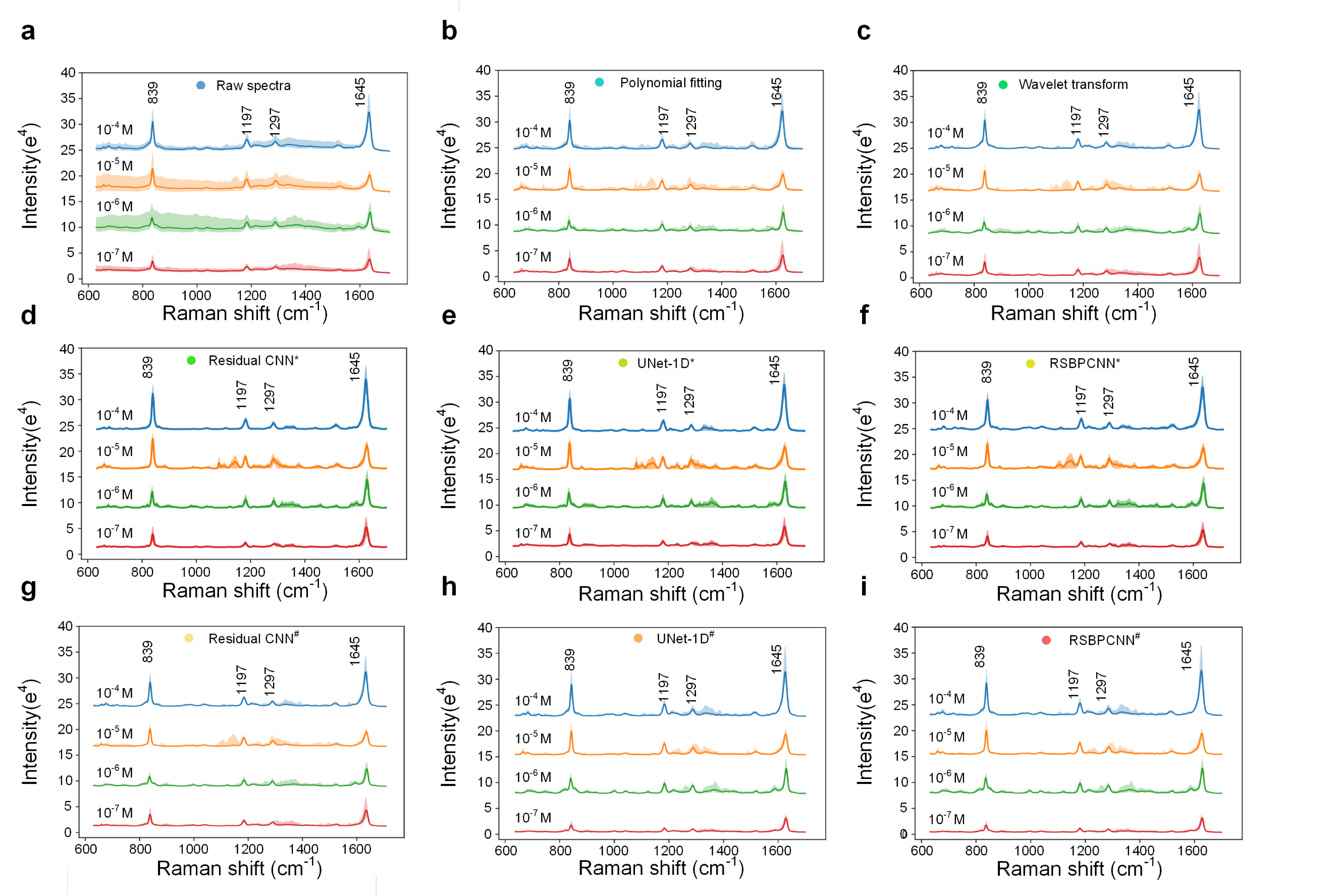


**Figure S12. Normalized SERS spectra for paraquat quantitation.** (a) Curves and shadows show the normalized spectra with mean ± SD before preprocessing. (b) Spectra preprocessed by Polynomial fitting. (c) Spectra preprocessed by Wavelet transform. (d) Spectra preprocessed by Residual CNN^*^. (e) Spectra preprocessed by UNet-1D^*^. (f) Spectra preprocessed by RSBPCNN^*^. (g) Spectra preprocessed by Residual CNN^#^. (h) Spectra preprocessed by UNet-1D^#^. (i) Spectra preprocessed by RSBPCNN^#^. ^*^: Representing that the model was trained with mathematical simulation datasets. ^#^: represents that the model was trained using RSGAN-generated datasets.


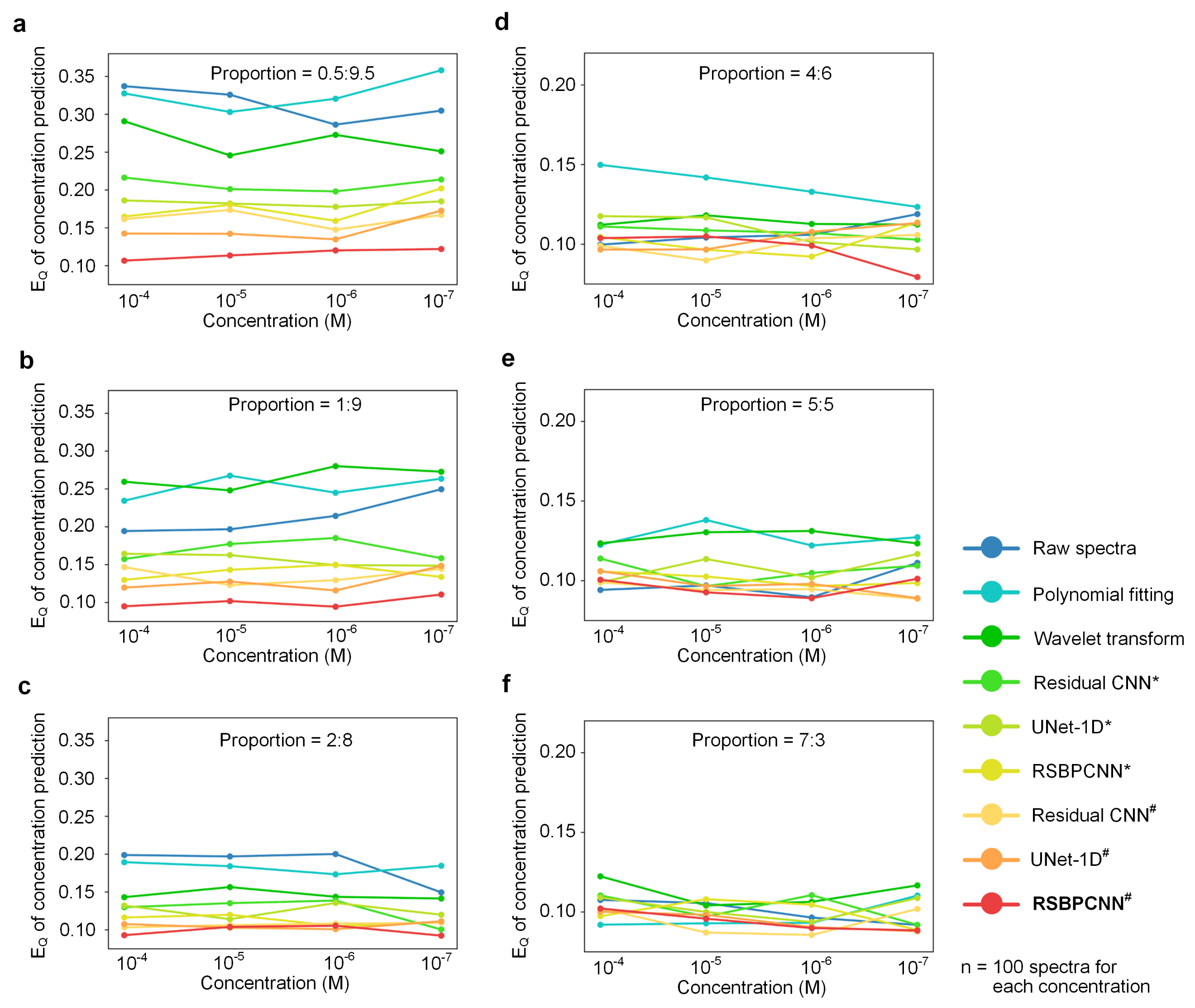


**Figure S13.** **Prediction**$\boldsymbol{E}_{\boldsymbol{Q}}$ **of spectra preprocessed by different methods at various training data and validation data proportions.**


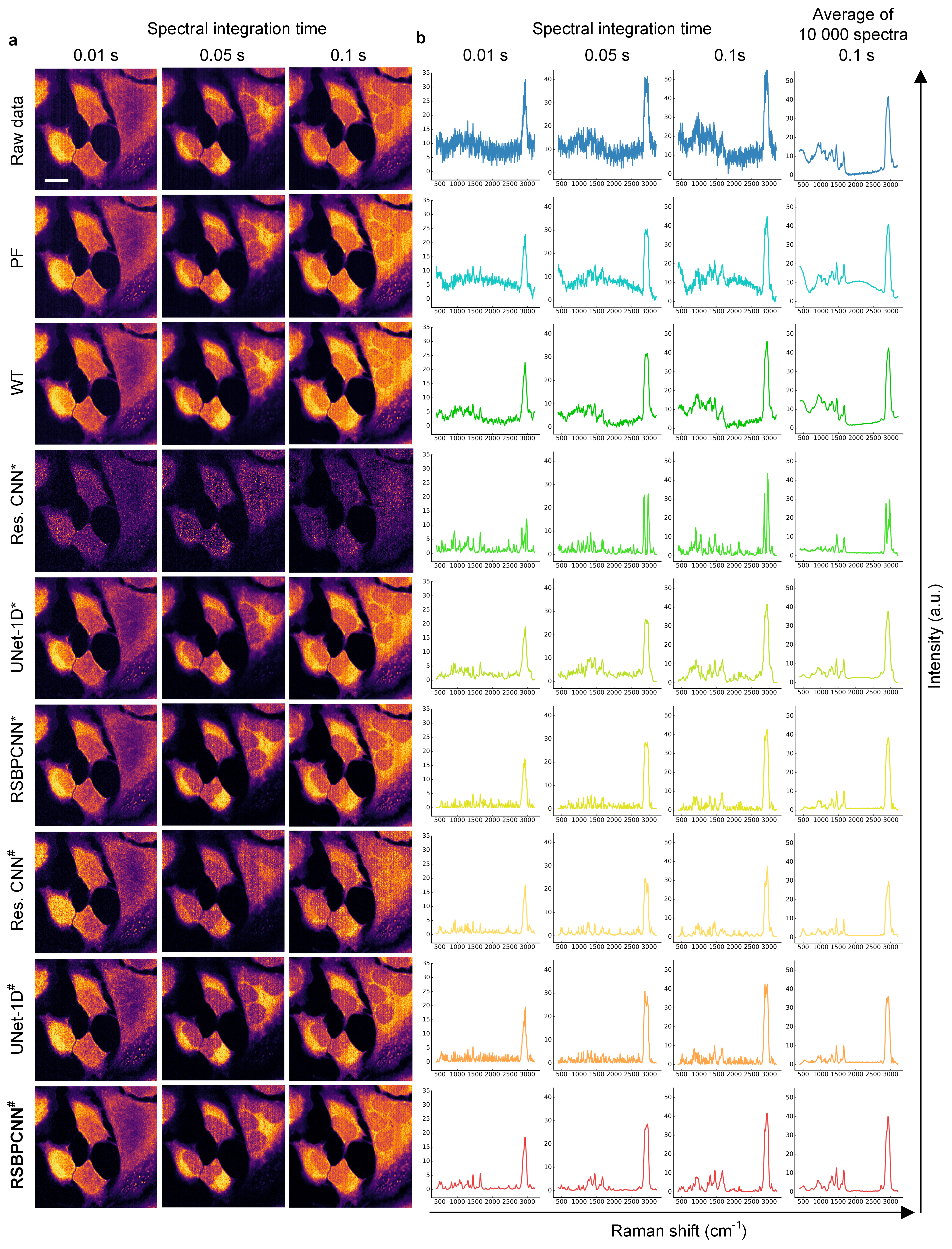


**Figure S14. Raman hyperspectral image quality enhancement with different integration times.** (a) Visualization of the raw or preprocessed Hela cell line spectral images at the protein (CH_3_, 2928 cm^-1^) channel. The rows indicate spectral integration times. The columns indicate preprocessing methods. (b) One or average spectrum of the raw or preprocessed Hela cell line image spectra. PF: Polynomial fitting; WT: Wavelet transform; Res. CNN: Residual CNN. Scale bar: 20 µm. ^*^: The training dataset is mathematical simulation. ^#^: The training dataset is RSGAN-generated spectra.


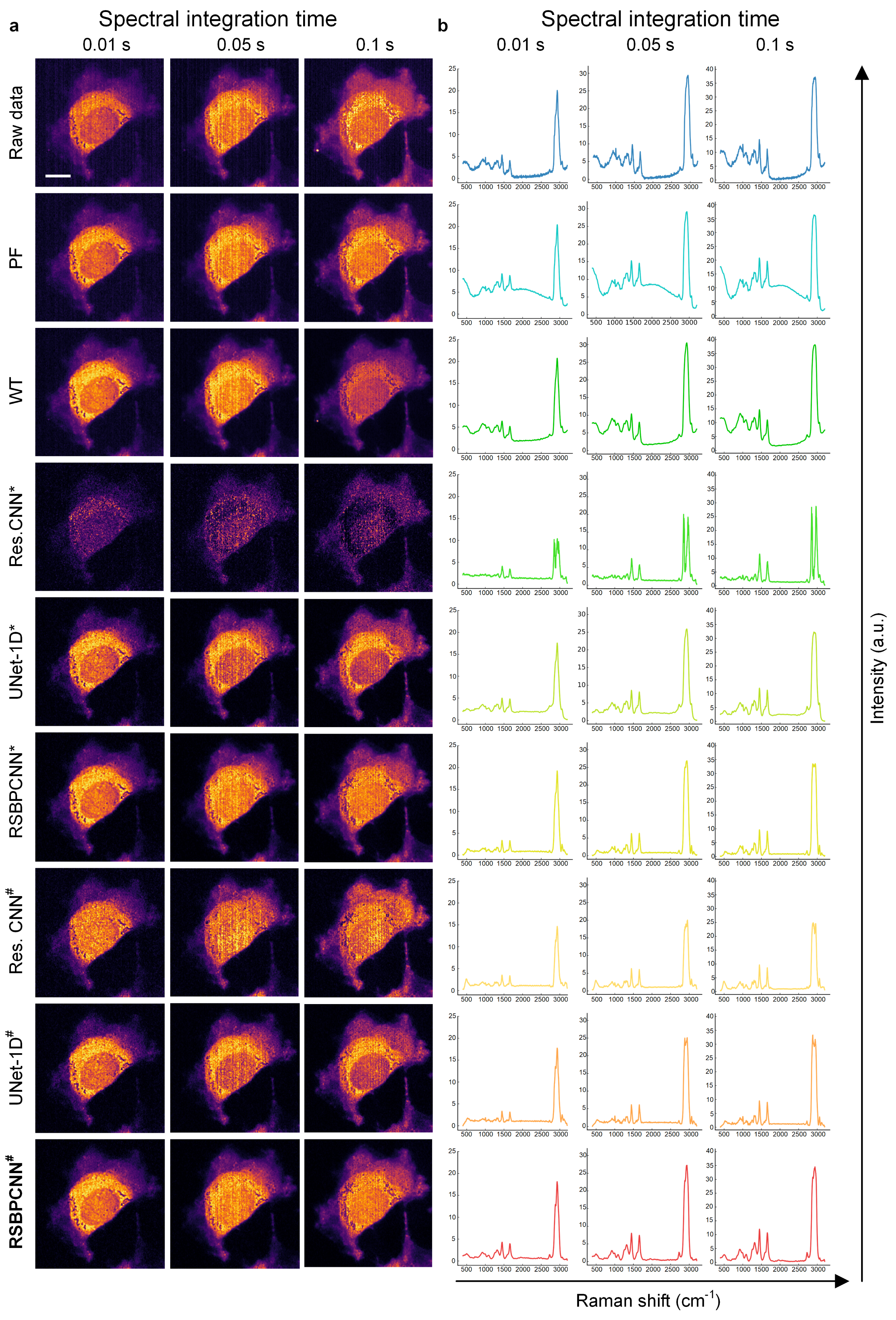


**Figure S15. Raman hyperspectral image quality enhancement with different integration times.** (a) Visualization of the raw or preprocessed COS-7 cell line spectral images at the protein (CH_3_, 2928 cm^-1^) channel. The rows indicate spectral integration times. The columns indicate preprocessing methods. (b) Average spectra of 10 000 data with top SNR_spec_ of the raw or preprocessed COS-7 cell line hyperspectral image spectra. PF: Polynomial fitting; WT: Wavelet transform; Res. CNN: Residual CNN. Scale bar: 8 μm. ^*^: The training dataset is mathematical simulation. ^#^: The training dataset is RSGAN-generated spectra.


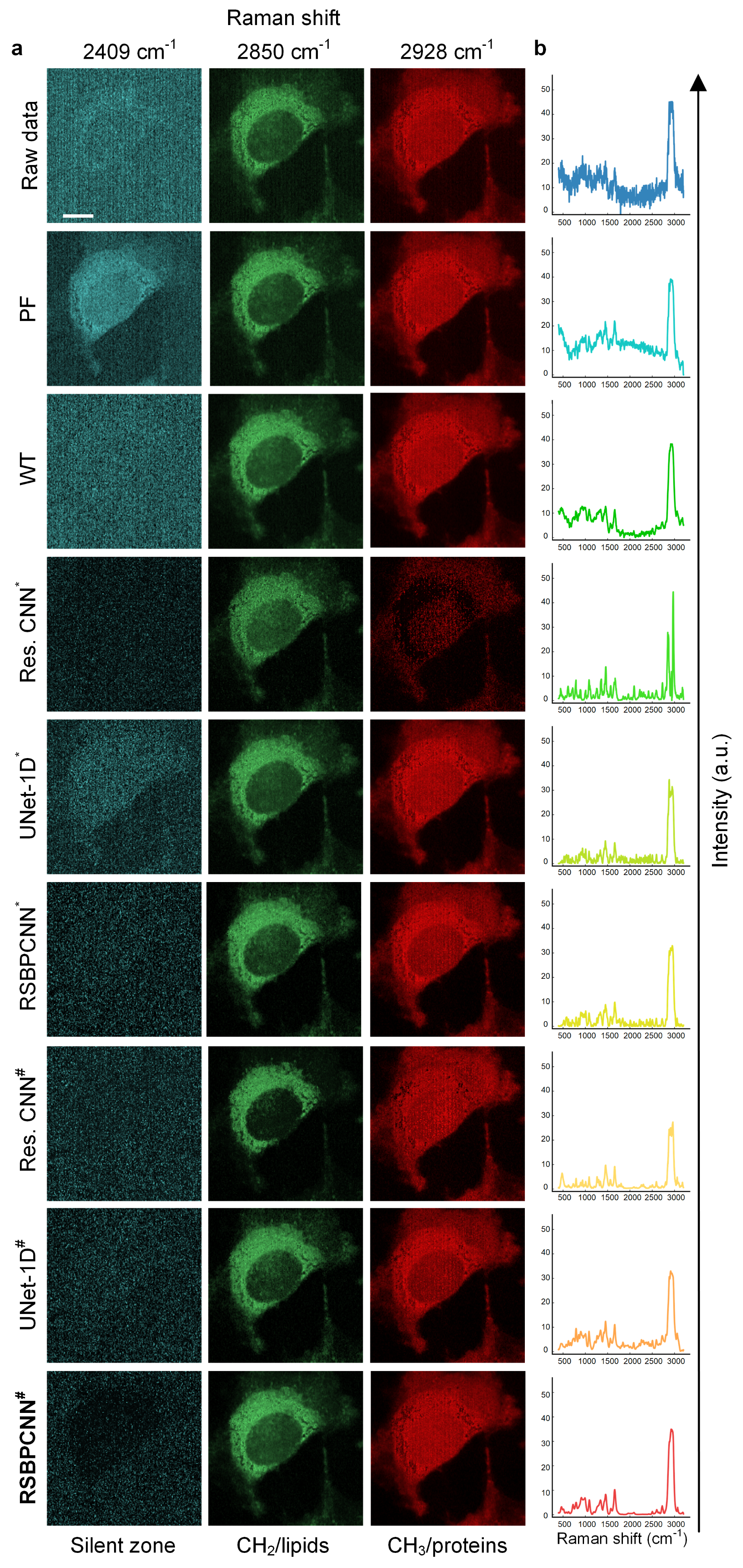


**Figure S16. Raman hyperspectral image quality.** (a) Visualization of the raw data and preprocessed COS-7 cell line hyperspectral images at the silent zone (2409 cm^-1^), lipids (CH_2_, 2850 cm^-1^) and proteins (CH_3_, 2928 cm^-1^) channels. The rows indicate Raman shift channels. The columns indicate preprocessing methods. (b) One randomly selected spectrum with integration time of 0.1 s for visual inspection. PF: Polynomial fitting; WT: Wavelet transform; Res. CNN: Residual CNN. ^*^: The training dataset is mathematical simulation. ^#^: The training dataset is RSGAN-generated spectra. Scale bar: 8 μm.


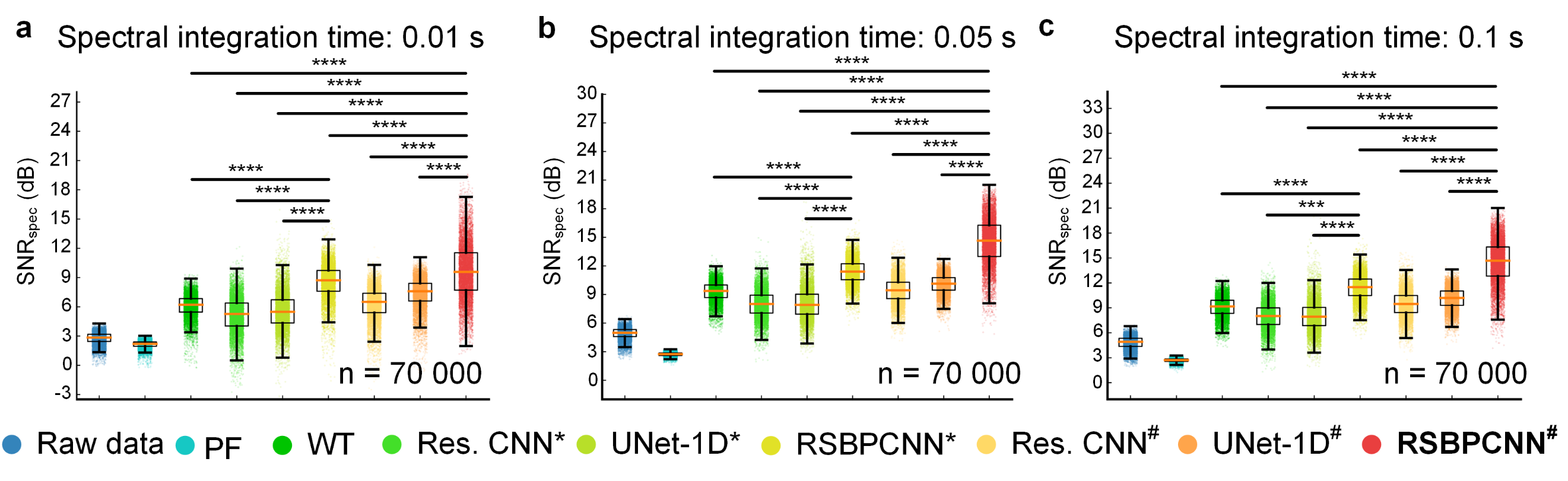


**Figure S17. SNR of the raw or preprocessed spectral images with different spectral integration times.** (a) Spectral integration time of 0.01 s. (b) Spectral integration time of 0.05 s. (c) Spectral integration times of 0.1 s. Statistical significance was accessed using the Mann-Whitney U Test for two independent samples. * Represents *P* value < 0.05, ** represents *P* value < 0.01, *** represents *P* value < 0.001, **** represents *P* value < 0.0001. PF: Polynomial fitting; WT: Wavelet transform; Res. CNN: Residual CNN. ^*^: The training dataset is mathematical simulation. ^#^: The training dataset is RSGAN-generated spectra. n = 10 000 spectra/image * 7 images.


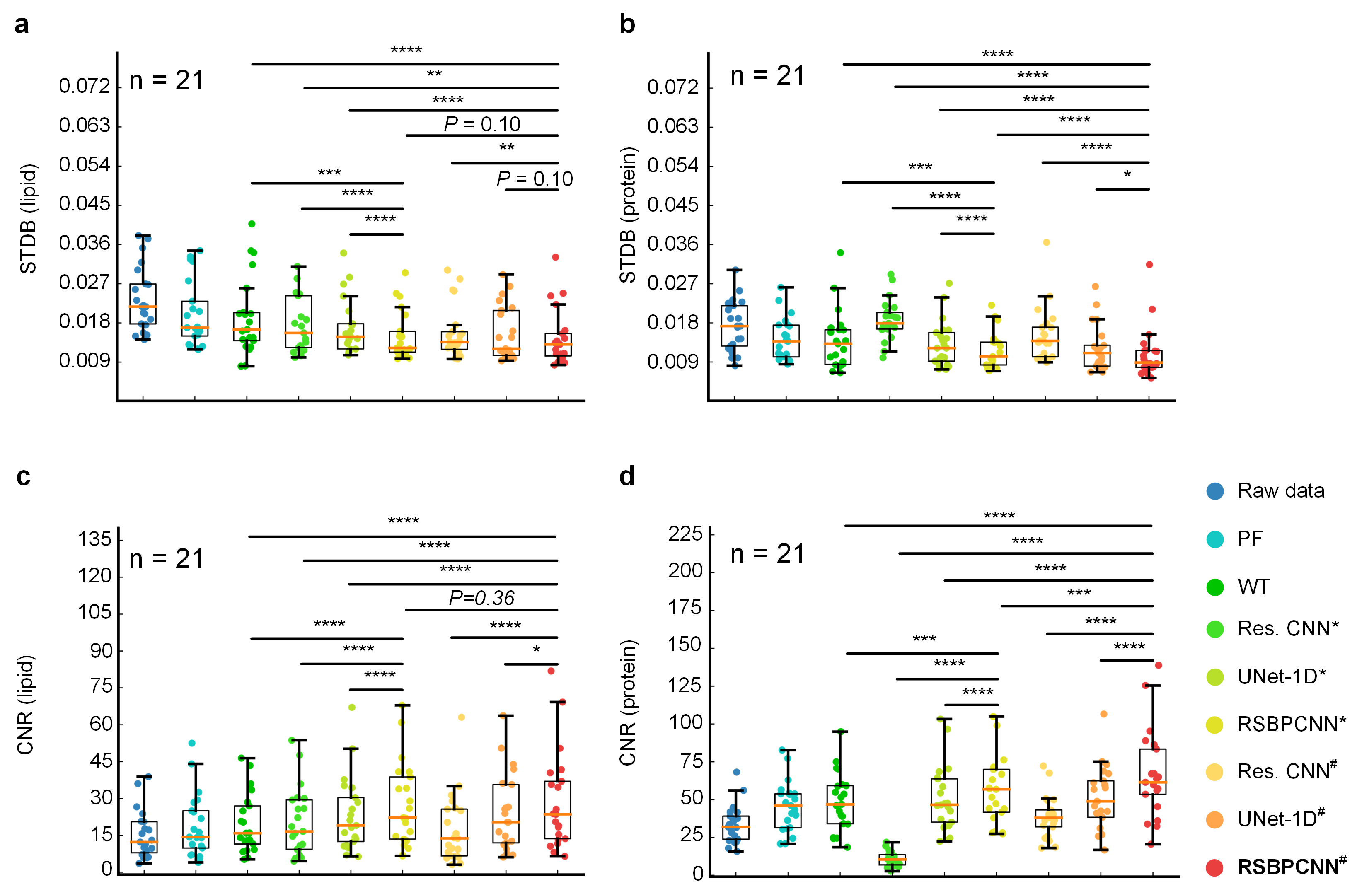


**Figure S18. STDB and CNR of the raw or preprocessed Raman spectral images with different preprocessing methods.** (a) STDB of images with a lipid channel (Raman shift of 2850 cm^-1^, CH_2_). (b) STDB of images with a protein channel (Raman shift of 2928 cm^-1^, CH_3_). (c) CNR of images with a lipid channel (Raman shift of 2850 cm^-1^, CH_2_). (d) CNR of images with a protein channel (Raman shift of 2928 cm^-1^, CH_3_). Statistical significance was accessed using the Wilcoxon signed-rank test for two correlated samples. * Represents *P* value < 0.05, ** represents *P* value < 0.01, *** represents *P* value < 0.001, **** represents *P* value < 0.0001. PF: Polynomial fitting; WT: Wavelet transform; Res. CNN: Residual CNN. ^*^: The training dataset is mathematical simulation. ^#^: The training dataset is RSGAN-generated spectra. n = 21 images.


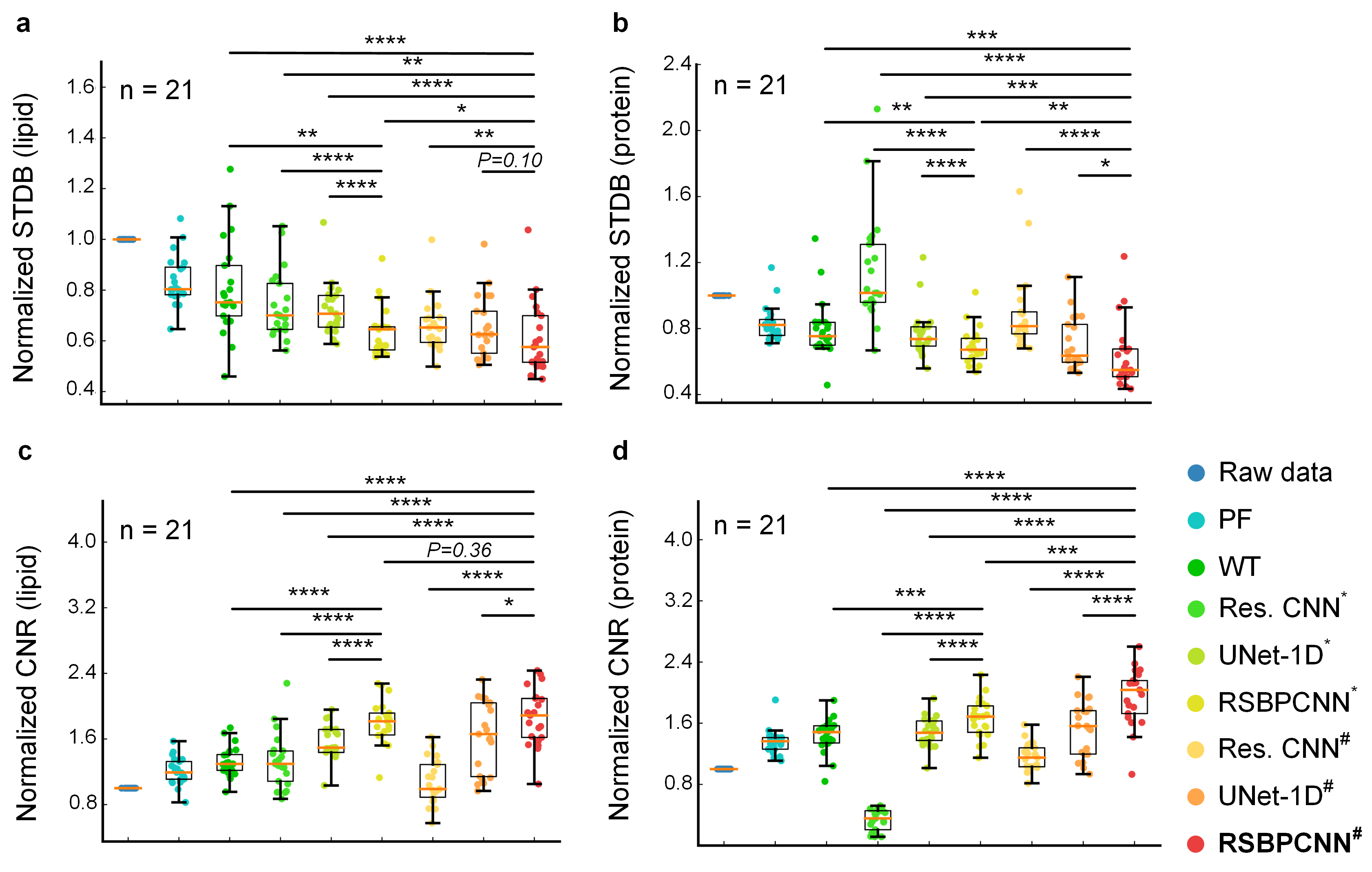


**Figure S19. Normalized STDB and normalized CNR of the raw or preprocessed Raman hyperspectral images with different preprocessing methods.** (a**)** Normalized STDB of images with a lipid channel (Raman shift of 2850 cm^-1^, CH_2_). (b**)** Normalized STDB of images with a protein channel (Raman shift of 2928 cm^-1^, CH_3_). (c) Normalized CNR of images with a lipid channel (Raman shift of 2850 cm^-1^, CH_2_). (d) Normalized CNR of images with a protein channel (Raman shift of 2928 cm^-1^, CH_3_). Statistical significance was accessed using the Wilcoxon signed-rank test for two correlated samples. * Represents *P* value < 0.05, ** represents *P* value < 0.01, *** represents *P* value < 0.001, **** represents *P* value < 0.0001. PF: Polynomial fitting; WT: Wavelet transform; Res. CNN: Residual CNN. ^*^: The training dataset is mathematical simulation. ^#^: The training dataset is RSGAN-generated spectra. n = 21 images.


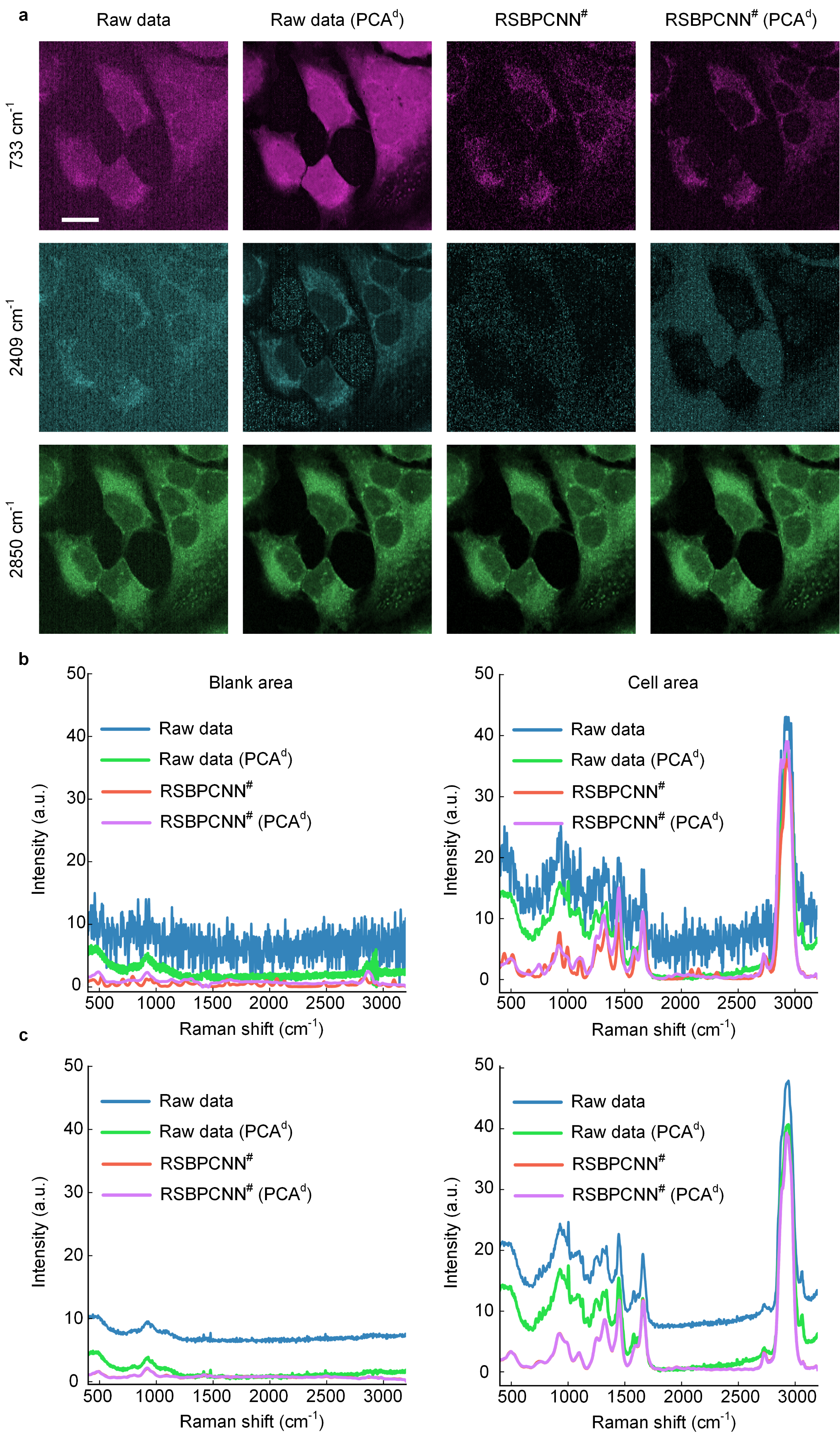


**Figure S20**. (a) Representative preprocessed Raman spectral image of the Hela cell line mapped with Raman shifts of phosphatidylserine (733 cm^-1^), spectral silent zone (2409 cm^-1^), and lipid (2850 cm^-1^). (b) Visualization of one randomly selected spectrum with integration time of 0.1 s of the blank or cell area. (c) Visualization of average spectra of 10 000 data with low or top SNR_spec_ from the blank (left) or cell (right) area. A superscript pound sign ^#^ indicates that the training dataset of the model is RSGAN-generated spectra. PCA^d^: PCA denoising was conducted on the Raw data or RSBPCNN^#^ preprocessed hyperspectral images. Scale bar: 20 μm.


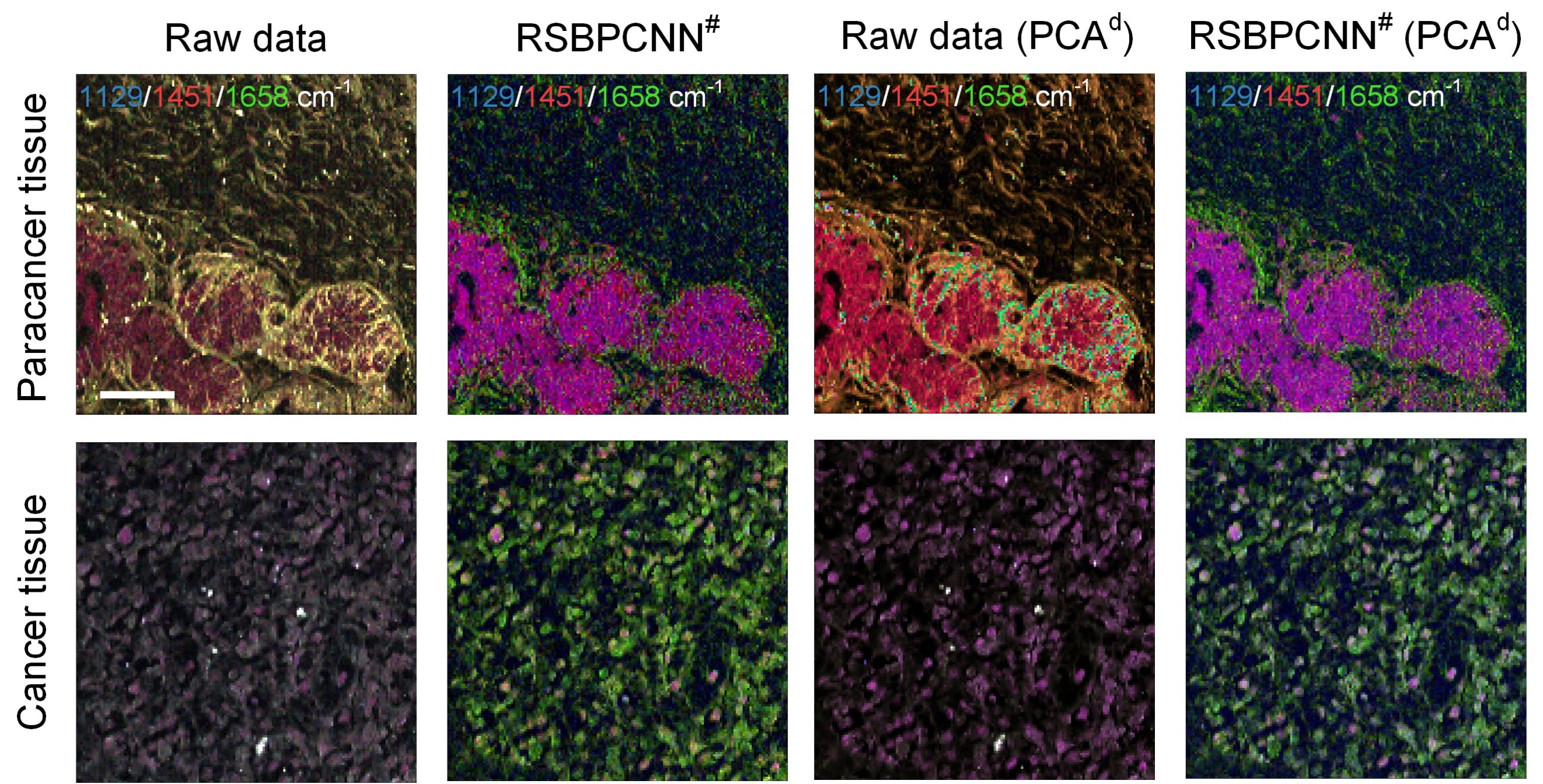


**Figure S21.** Merged images of paracancer and cancer tissues with Raman shifts of 1129 cm^-1^ (blue), 1451 cm^-1^ (red), and 1658 cm^-1^ (green) of the Raw data, RSBPCNN^#^, Raw data (PCA^d^), and RSBPCNN^#^ (PCA^d^) preprocessed Raman spectra. Scale bar: 40 μm.


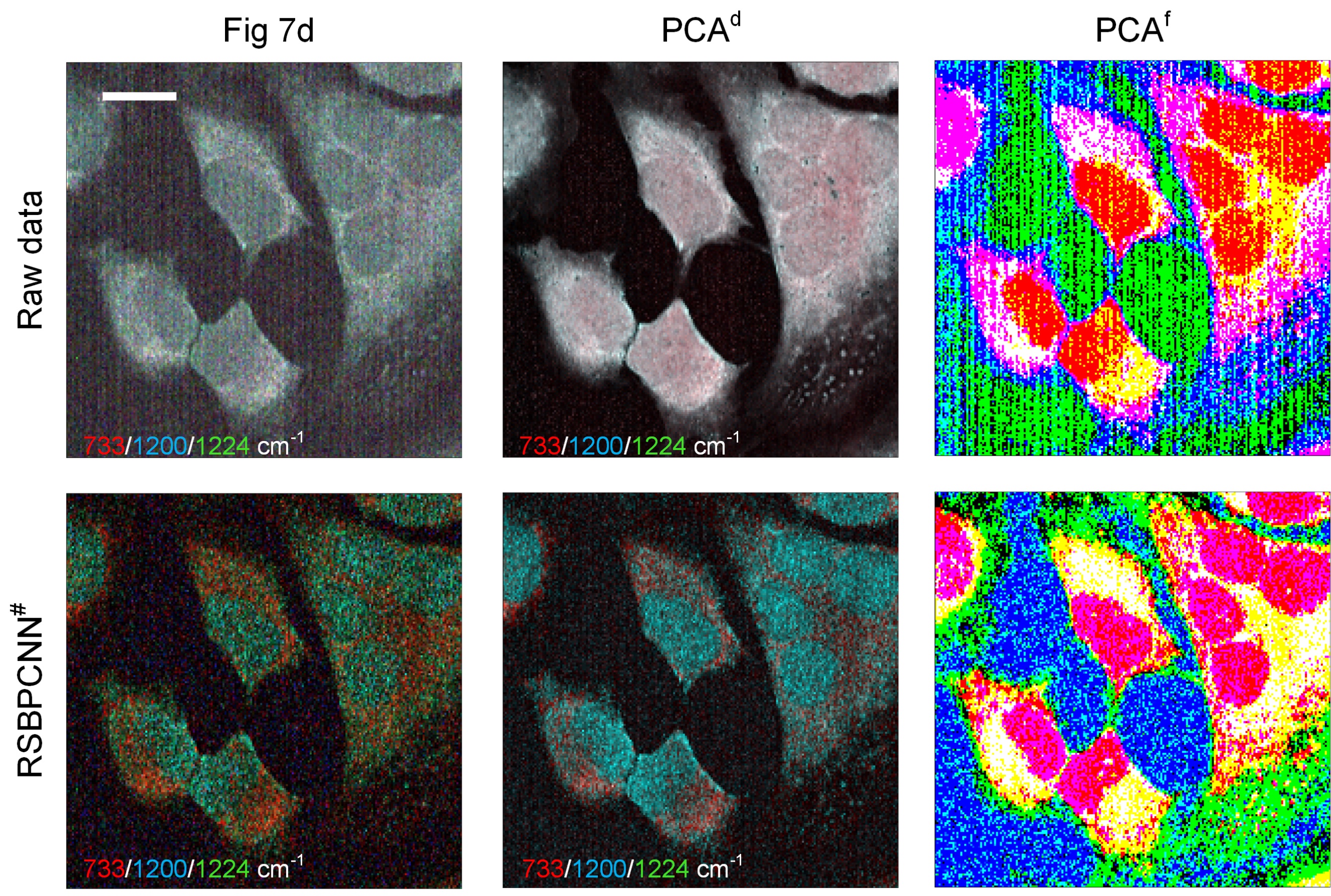


**Figure S22.** Merged images of the Hela cell line with Raman shifts of 733 cm^-1^ (red, phosphatidylserine), 1200 cm^-1^ (blue, nucleic acids & phosphates/C-O/C-N), and 1224 cm^-1^ (green, amide III (*β*-sheet structure)) of the Raw data or RSBPCNN^#^ preprocessed Raman spectral images. PCA^d^: PCA denoising was conducted on the Raw data or RSBPCNN^#^ preprocessed hyperspectral images. PCA^f^: PCA feature extraction was conducted on the Raw data or RSBPCNN^#^ preprocessed hyperspectral images. Scale bar: 20 μm.


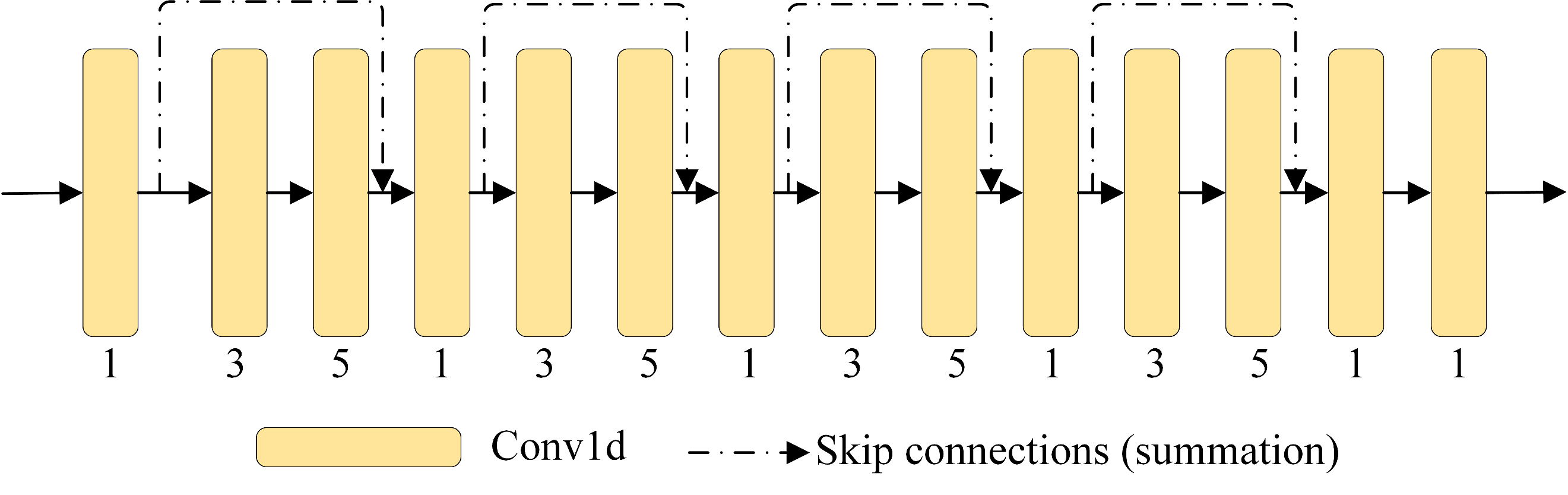


**Figure S23. Structure of the Residual CNN model.**


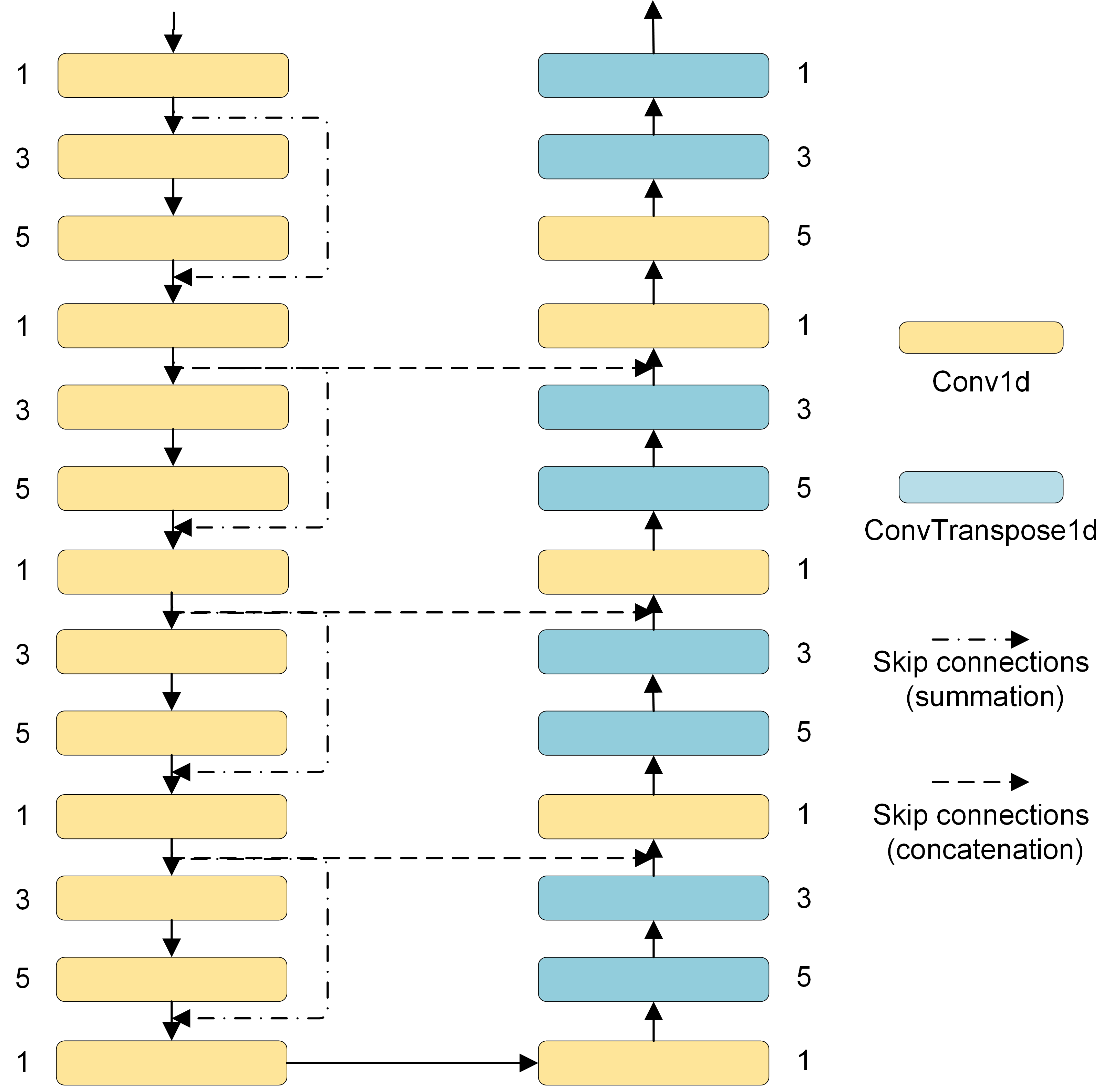


**Figure S24. Structure of the UNet-1D model.**


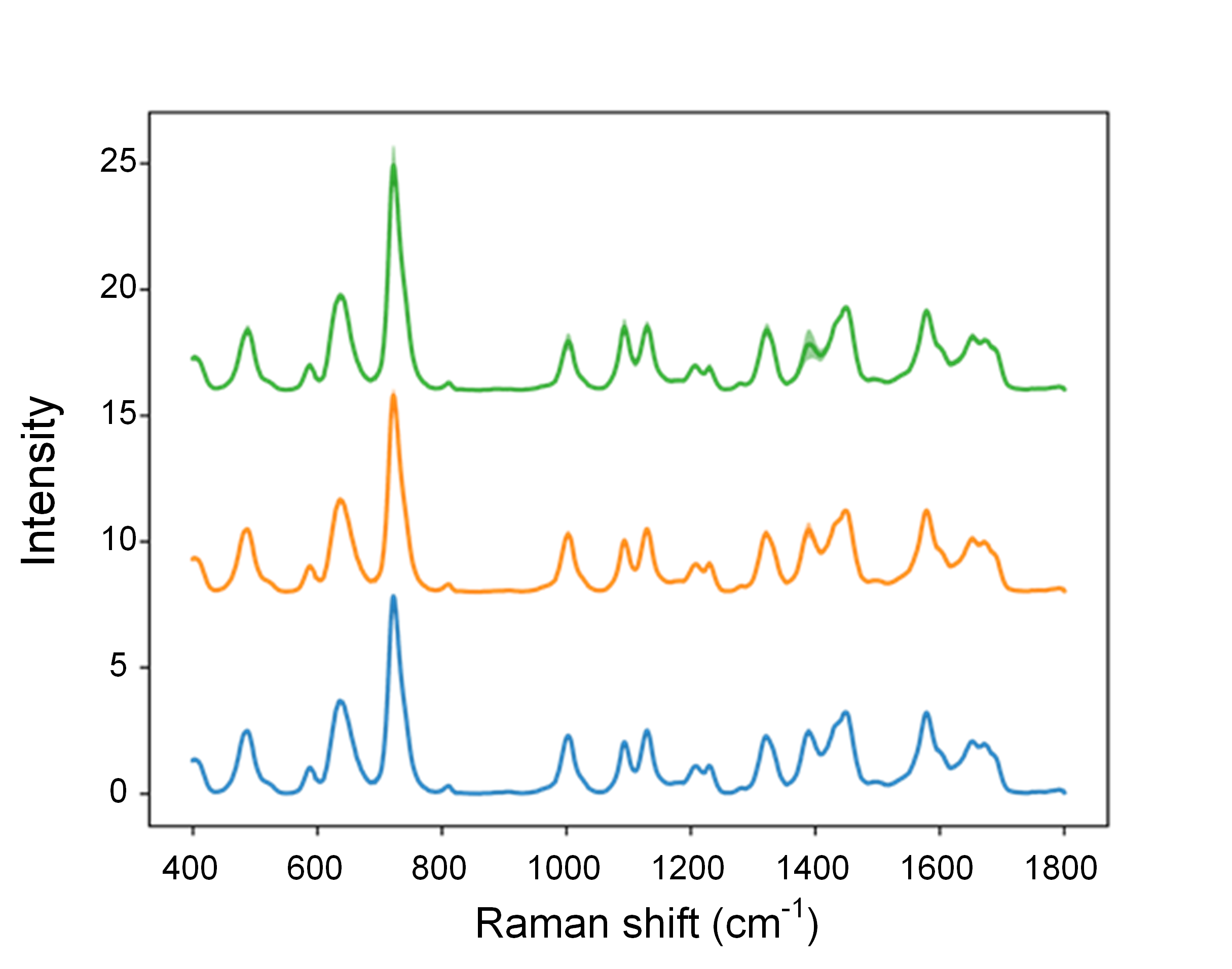


**Figure S25**. Curves and shadows show the normalized serum SERS spectra with mean ± SD collected from three independent replicates of healthy human serum. n = 10 spectra for each curve.


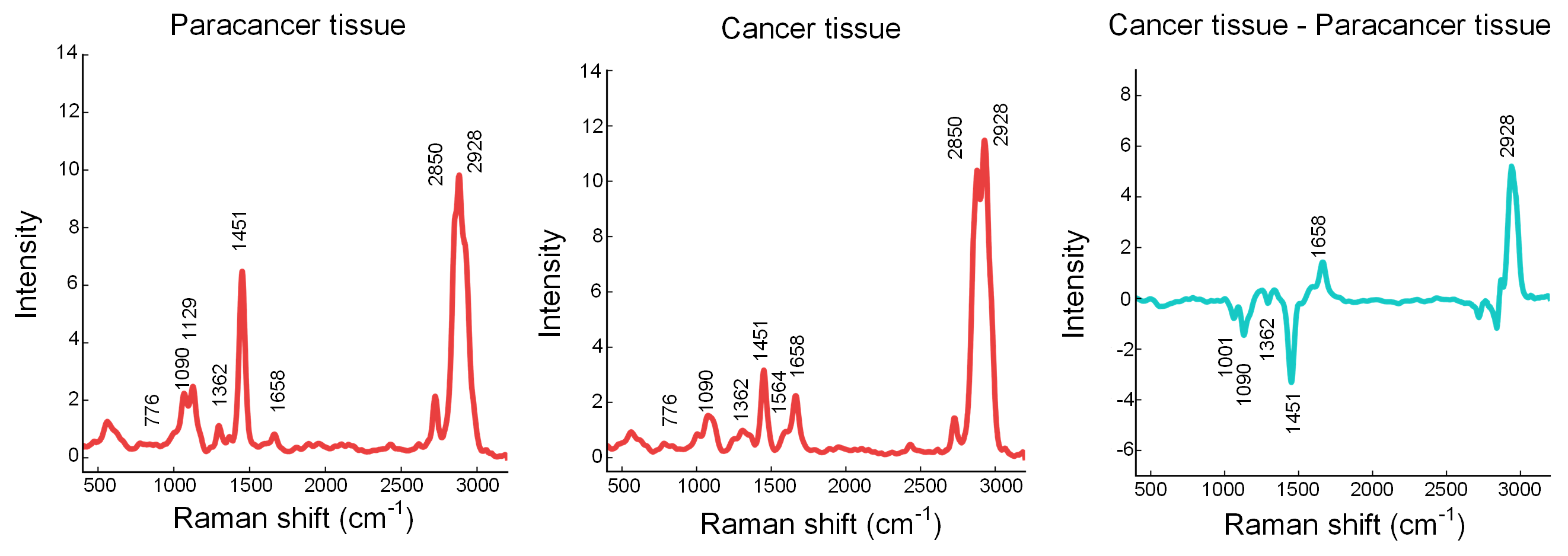


**Figure S26**. RSBPCNN^#^ preprocessed spectra of tissues (average of 10 000 spectra). The green curve indicates the subtraction of cancer and paracancer tissue average spectra.

# Supplementary Tables

**Table S1**. Spectral diversity

| **Experiment** | **Dataset** | **Instrument;**  **Wavelength** | **Laboratory** |
| --- | --- | --- | --- |
| **RSPSSL Establishment**: Input datasets of the RSGAN model for the  generation of diverse spectra with corresponding ideal spectra for the training of RSBPCNN | RRUFF database | N/A;  532/785 nm | Highlights in mineralogical crystallography, 2015, 1-30 |
|  | Other SERS spectra | LabRAM HR Evolution, Horiba; 532/633 nm | College of Optical and Electronic Technology, China Jiliang University |
| **RSPSSL Validation**:  Cancer diagnosis trial | Serum SERS spectra | Portman785, Oceanhood; 785 nm | Department of Electronic and Electrical Engineering, Southern University of Science and Technology |
| **RSPSSL Validation**:  Concentration prediction trial | Paraquat solution SERS spectra | Renishaw Ina; 785 nm | College of Optical and Electronic Technology, China Jiliang University |
| **RSPSSL Validation:**  Hyperspectral image preprocessing trial | Cells and tissue hyperspectral images | WITec Alpha 300R; 532 nm | Department of Ocean Science and Engineering, Southern University of Science and Technology |

**Table S2.** RMSE and [infinite norm](javascript:;) values of spectra preprocessed by different preprocessing methods.

| Preprocessing method | | RMSE | | [Infinite norm](javascript:;) | | Validation data size (spectra) |  |
| --- | --- | --- | --- | --- | --- | --- | --- |
| Catalog | Name | Mean | SD | Mean | SD |  |  |
| Original data | Raw spectra | 256.40 | 140.15 | 10.971 | 6.391 | 100 |  |
| **Established** mathematical method | Polynomial fitting | 0.8202 | 0.3374 | 2.0732 | 1.2139 |  |  |
|  | Wavelet transform | **0.0498** | 0.0299 | **0.1375** | 0.0788 |  |  |
| **Established** deep learning method | Residual CNN* | 0.0621 | 0.0386 | 0.1875 | 0.1645 |  |  |
|  | UNet-1D* | 0.0594 | 0.0255 | 0.1503 | 0.0891 |  |  |
| Ablation analysis of this work | RSBPCNN* | 0.0404 | 0.0205 | 0.1130 | 0.0660 |  |  |
|  | Residual CNN^#^ | 0.0547 | 0.0336 | 0.1497 | 0.0814 |  |  |
|  | UNet-1D^#^ | 0.0271 | 0.0180 | 0.0983 | 0.0623 |  |  |
| **This work: RSPSSL** | **RSBPCNN^#^** | **0.0058** | 0.0042 | **0.0553** | 0.0392 |  |  |
| Original data | Raw spectra^3000^ | 253.69 | 147.95 | 11.152 | 6.333 | 3 000 |  |
| **Established** deep learning method | Residual CNN*^3000^ | 0.0665 | 0.0404 | 0.2030 | 0.1137 |  |  |
|  | UNet-1D*^3000^ | **0.0613** | 0.0282 | **0.1565** | 0.0912 |  |  |
| Ablation analysis of this work | RSBPCNN*^3000^ | 0.0422 | 0.0323 | 0.1302 | 0.0880 |  |  |
|  | Residual CNN^#3000^ | 0.0629 | 0.0323 | 0.1604 | 0.0879 |  |  |
|  | UNet-1D^#3000^ | 0.0309 | 0.0210 | 0.1094 | 0.0637 |  |  |
| **This work**: **RSPSSL** | **RSBPCNN^#3000^** | **0.0062** | 0.0041 | **0.0596** | 0.0412 |  |  |
| **^*^: models trained using mathematical simulation datasets.**  **^#^: models trained using RSGAN-generated datasets.**  *^3000^ and ^#3000^: average of 3 000 spectra.  The smallest RMSE and [infinite norm](javascript:;) values of the established methods and this work are highlighted in bold. | | | | | | | |

**Table S3**. Cancer diagnostic accuracy of spectra preprocessed by different preprocessing methods at different training sample sizes.

| Preprocessing  methods | Training sample size (case) | | | | | | |
| --- | --- | --- | --- | --- | --- | --- | --- |
|  | 6 | 8 | 9 | 10 | 15 | 20 | 22 |
| Raw spectra | 0.54  (0.51-0.57) | 0.57  (0.54-0.60) | 0.61  (0.57-0.65) | 0.63  (0.58-0.66) | 0.73  (0.67-0.75) | 0.80  (0.72-0.87) | 0.81  (0.72~0.88) |
| Polynomial fitting | 0.55  (0.51-0.58) | 0.58  (0.53-0.62) | 0.60  (0.55-0.64) | 0.64  (0.59-0.67) | 0.72  (0.68-0.76) | 0.81  (0.74-0.85) | 0.82  (0.75~0.85) |
| Wavelet transform | **0.56**  (0.51-0.59) | 0.59  (0.53-0.64) | 0.63  (0.54-0.67) | 0.65  (0.59-0.68) | 0.75  (0.70-0.79) | 0.82  (0.73-0.85) | 0.83  (0.74~0.85) |
| Residual CNN^*^ | 0.54  (0.51-0.57) | 0.58  (0.52-0.63) | 0.61  (0.56-0.67) | 0.64  (0.59-0.68) | 0.74  (0.68-0.79) | 0.80  (0.71-0.85) | 0.82  (0.74~0.86) |
| UNet-1D^*^ | 0.55  (0.51-0.58) | 0.60  (0.54-0.65) | 0.64  (0.57-0.68) | 0.67  (0.62-0.70) | 0.76  (0.71-0.80) | 0.82  (0.75-0.84) | 0.83  (0.76~0.85) |
| RSBPCNN^*^ | 0.55  (0.51-0.59) | 0.59  (0.53-0.64) | 0.65  (0.58-070) | 0.68  (0.63-0.71) | 0.77  (0.71-0.81) | 0.83  (0.77-0.86) | 0.85  (0.79~0.87) |
| Residual CNN^#^ | 0.54  (0.51-0.57) | 0.58  (0.52-0.63) | 0.64  (0.55-0.71) | 0.73  (0.67-0.77) | 0.80  (0.76-0.83) | 0.84  (0.78~0.88) | 0.85  (0.78~0.88) |
| UNet-1D^#^ | 0.55  (0.51-0.58) | 0.62  (0.54-0.67) | 0.69  (0.62-0.77) | 0.75  (0.68-0.79) | 0.81  (0.77-0.83) | 0.85  (0.81-0.90) | 0.86  (0.82~0.90) |
| RSBPCNN^#^ | **0.56**  (0.52-0.58) | **0.64**  (0.55-0.69) | **0.74**  (0.67-0.80) | **0.82**  (0.75-0.85) | **0.87**  (0.83-0.90) | **0.90**  (0.85-0.93) | **0.91**  (0.85~0.94) |
| Data are presented by mean (95% CI).  AUC: area under the receiver operating characteristic (ROC) curve.  ^*^: models trained with mathematical simulation datasets.  ^#^: models trained with RSGAN-generated datasets.  The highest mean value of each experimental group is highlighted in bold. | | | | | | | |

**Table S4.** Paraquat concentration prediction$E_{Q}$ using spectra preprocessed by different methods.

| Processing method | Proportion | | | | | |
| --- | --- | --- | --- | --- | --- | --- |
|  | 0.5:9.5 | 1:9 | 2:8 | 4:6 | 5:5 | 7:3 |
| Raw spectra  Polynomial fitting  Wavelet transform  Residual CNN^*^  UNet-1D^*^  RSBPCNN^*^  Residual CNN^#^  UNet-1D^#^  RSBPCNN^#^ | 0.3135  0.3273  0.2651  0.2074  0.1828  0.1767  0.1625  0.1481  **0.1156** | 0.2138  0.2565  0.2017  0.1696  0.1563  0.1392  0.1359  0.1279  **0.1005** | 0.1864  0.1829  0.1426  0.1261  0.1255  0.1130  0.1068  0.1057  **0.0987** | 0.1072  0.1370  0.1138  0.1074  0.1081  0.1017  0.0996  0.1036  **0.0968** | 0.0980  0.1275  0.1271  0.1062  0.1078  0.1009  **0.0941**  0.0974  0.0959 | 0.1004  0.0971  0.1124  0.1025  0.1029  0.0997  0.0942  0.0942  **0.0941** |
| Proportion = Training set: Validation set (n = 100 spectra per concentration).  ^*^: models are trained using mathematical simulation datasets.  ^#^: models are trained using RSGAN-generated datasets.  The smallest average $E_{Q}$values are highlighted in bold. | | | | | | |

**Table S5.** Acquisition parameters of the biomedical hyperspectral images.

| Sample | No. | Laser wavelength  (nm) | Laser  power  (mW) | Integration  time (s) | Objective  /NA | n Pixels  (spectra) |
| --- | --- | --- | --- | --- | --- | --- |
| Hela cell  Hela cell  Hela cell  COS-7 cell  COS-7 cell  COS-7 cell  COS-7 cell  Paracancer tissue  Cancer tissue | 1  2  3  1  2  3  4  1  1 | 532  532  532  532  532  532  532  532  532 | 23  23  23  23  23  23  23  15  15 | 0.01s/0.05s/0.1s  0.01s/0.05s/0.1s  0.01s/0.05s/0.1s  0.01s/0.05s/0.1s  0.01s/0.05s/0.1s  0.01s/0.05s/0.1s  0.01s/0.05s/0.1s  0.01s  0.05s | 100x / 0.9  100x / 0.9  100x / 0.9  100x / 0.9  100x / 0.9  100x / 0.9  100x / 0.9  50x / 0.75  50x / 0.75 | 40 000  40 000  40 000  40 000  40 000  40 000  40 000  22 500  22 500 |

**Table S6.** SNR_spec_, normalized STDB, and normalized CNR of top 1 0000 spectra per images preprocessed by different methods.

| Preprocessing algorithm | Evaluation index | | | | | | | | |
| --- | --- | --- | --- | --- | --- | --- | --- | --- | --- |
|  | SNR_spec_ | | | Normalized STDB | | | Normalized CNR | | |
|  | Mean | SD | n  (spectra) | Mean | SD | n  (image) | Mean | SD | n  (image) |
| Raw data  Polynomial fitting  Wavelet transform  Res CNN^*^  UNet-1D^*^  RSBPCNN^*^  Res CNN^#^  UNet-1D^#^  RSBPCNN^#^ | 4.04  2.47  8.01  6.87  7.05  10.03  8.34  9.55  **12.59** | 0.51  0.19  0.74  0.75  0.63  1.02  0.88  0.6  1.21 | 210k  210k  210k  210k  210k  210k  210k  210k  210k | 1.0  0.84  0.80  0.95  0.75  0.66  0.78  0.68  **0.62** | 0.0  0.10  0.18  0.32  0.13  0.11  0.21  0.14  0.17 | 42  42  42  42  42  42  42  42  42 | 1.0  1.28  1.38  0.83  1.52  1.73  1.12  1.58  **1.91** | 0.0  0.18  0.21  0.56  0.20  0.27  0.23  0.40  0.36 | 42  42  42  42  42  42  42  42  42 |
| SNR_spec_: the signal-to-noise ratio of spectra.  Normalized STDB: normalized standard deviation of background.  Normalized CNR: normalized contrast-to-noise ratio.  ^*^: models trained using mathematical simulation datasets.  ^#^: models trained using RSGAN-generated datasets. | | | | | | | | | |

**Table S7.** SNR_spec_ of spectra with different integration times.

| Preprocessing algorithm | Evaluation index | | | | | | | | | | | | | | |
| --- | --- | --- | --- | --- | --- | --- | --- | --- | --- | --- | --- | --- | --- | --- | --- |
|  | SNR_spec_ (0.01 s) | | | SNR_spec_ (0.05 s) | | | SNR_spec_ (0.1 s) | | | SNR_spec_ (0.4 s) | | | | |  |
|  | Mean | SD | n | Mean | SD | n | Mean | SD | n | | Mean | SD | n |  |  |
| Raw data  Polynomial fitting  Wavelet transform  Res CNN^*^  UNet-1D^*^  RSBPCNN^*^  Res CNN^#^  UNet-1D^#^  RSBPCNN^#^ | 2.88  2.14  6.26  5.10  5.63  8.27  6.28  7.55  **9.68** | 0.62  0.39  1.18  1.84  1.92  1.78  1.66  1.56  2.82 | 70k  70k  70k  70k  70k  70k  70k  70k  70k | 4.21  2.56  8.29  7.18  7.31  10.3  8.57  9.81  **13.04** | 0.63  0.21  1.05  1.46  1.72  1.29  1.35  1.01  2.42 | 70k  70k  70k  70k  70k  70k  70k  70k  70k | 5.04  2.73  9.50  8.31  8.22  11.54  10.16  11.28  **15.06** | 0.76  0.23  1.23  1.57  1.80  1.50  1.59  1.27  2.64 | 70k  70k  70k  70k  70k  70k  70k  70k  70k | | 6.29  4.12  11.07  10.75  10.31  13.93  12.04  13.81  **18.72** | 0.87  0.31  1.35  1.72  1.89  1.75  1.63  1.43  2.8 | 20k  20k  20k  20k  20k  20k  20k  20k  20k |  |  |
| SNR_spec_: the signal-to-noise ratio of spectra. n = number of spectra.  Normalized STDB: normalized standard deviation of background.  Normalized CNR: normalized contrast-to-noise ratio.  ^*^: models trained using mathematical simulation datasets.  ^#^: models trained using RSGAN-generated datasets. | | | | | | | | | | | | | | | |

**Table S8.** STDB and CNR values of biomedical hyperspectral images (Raman shifts of 2850 cm^-1^ and 2928 cm^-1^) preprocessed by different spectral preprocessing methods.

| Preprocessing algorithm | Evaluation index | | | | | | | | | | | | | | | | |
| --- | --- | --- | --- | --- | --- | --- | --- | --- | --- | --- | --- | --- | --- | --- | --- | --- | --- |
|  | STDB (lipid) | | | CNR (lipid) | | | STDB ([protein](javascript:;)) | | | | CNR ([protein](javascript:;)) | | | | | | |
|  | Mean | SD | n | Mean | SD | n | Mean | SD | | n | Mean | | SD | | n | | |
| Raw data  Polynomial fitting  Wavelet transform  Res CNN^*^  UNet-1D^*^  RSBPCNN^*^  Res CNN^#^  UNet-1D^#^  RSBPCNN^#^ | 0.0236  0.0200  0.0195  0.0175  0.0171  0.0151  0.0158  0.0156  **0.0145** | 0.007  0.007  0.009  0.006  0.006  0.005  0.006  0.006  0.006 | 21  21  21  21  21  21  21  21  21 | 15.18  18.83  19.76  20.47  21.42  21.36  17.57  24.36  **28.58** | 9.51  12.66  11.77  13.42  14.85  16.84  13.98  15.4  19.42 | 21  21  21  21  21  21  21  21  21 | 0.0172  0.0143  0.0140  0.0187  0.0132  0.0118  0.0152  0.0122  **0.0109** | | 0.006  0.005  0.007  0.005  0.005  0.004  0.006  0.005  0.006 | 21  21  21  21  21  21  21  21  21 | | 33.69  45.62  48.71  10.67  50.56  55.83  38.71  51.11  **66.35** | | 12.30  16.29  18.91  4.89  21.27  21.29  13.70  20.7  28.4 | | 21  21  21  21  21  21  21  21  21 | |
| STDB: standard deviation of back-ground. CNR: contrast-to-noise ratio. n = number of images  ^*^: models trained using mathematical simulation datasets.  ^#^: models trained using RSGAN-generated datasets. | | | | | | | | | | | | | | | | |  |

**Table S9.** Normalized STDB and normalized CNR values of biomedical hyperspectral images (Raman shifts of 2850 cm^-1^ and 2928 cm^-1^) preprocessed by different spectral preprocessing methods.

| Preprocessing algorithm | Evaluation index | | | | | | | | | | | | |
| --- | --- | --- | --- | --- | --- | --- | --- | --- | --- | --- | --- | --- | --- |
|  | Normalized  STDB (lipid) | | | Normalized  STDB ([protein](javascript:;)) | | | Normalized  CNR (lipid) | | | Normalized  CNR ([protein](javascript:;)) | | | |
|  | Mean | SD | n | Mean | SD | n | Mean | SD | n | Mean | SD | n | |
| Raw data  Polynomial fitting  Wavelet transform  Res CNN^*^  UNet-1D^*^  RSBPCNN^*^  Res CNN^#^  UNet-1D^#^  RSBPCNN^#^ | 1.0  0.84  0.81  0.74  0.73  0.64  0.66  0.65  **0.61** | 0.0  0.10  0.19  0.13  0.10  0.09  0.10  0.12  0.14 | 21  21  21  21  21  21  21  21  21 | 1.0  0.83  0.80  1.15  0.77  0.69  0.90  0.71  **0.63** | 0.0  0.10  0.18  0.33  0.14  0.11  0.23  0.16  0.19 | 21  21  21  21  21  21  21  21  21 | 1.0  1.21  1.32  1.33  1.56  1.80  1.08  1.63  **1.86** | 0.0  0.17  0.18  0.33  0.20  0.26  0.27  0.42  0.24 | 21  21  21  21  21  21  21  21  21 | 1.0  1.36  1.44  0.34  1.48  1.66  1.16  1.52  **1.95** | 0.0  0.16  0.22  0.14  0.19  0.25  0.18  0.36  0.37 | 21  21  21  21  21  21  21  21  21 | |
| ^*^: models trained using mathematical simulation datasets. ^#^: models trained using RSGAN-generated datasets. n = number of images | | | | | | | | | | | | |  |

**Table S10.** Performance and applicability comparisons of different preprocessing algorithms.

|  | Raw data | Raw data (PCA^d^) | RSBPCNN^#^ | RSBPCNN^#^ (PCA^d^) |
| --- | --- | --- | --- | --- |
| Single spectrum processing  Hyperspectral image processing  SNR_spec_ (dB)  Normalized CNR  Normalized STDB  Chemical resolution | -  -  4.04  1  1  **🗴** | **🗴**  **🗸**  5.95  1.47  0.83  **🗴** | **🗸**  **🗸**  12.59  1.91  0.62  **🗸** | **🗴**  **🗸**  12.97  2.08  0.61  **🗸-** |
| SNR_spec_: the signal-to-noise ratio of spectra.  Normalized STDB: normalized standard deviation of background.  Normalized CNR: normalized contrast-to-noise.  PCA^d^: PCA denoising. | | | | |

**Table S11.** Assignment and mechanism of serum SERS Raman shifts.

| Raman shift  (cm^-1^) | Assignment | Cancerous  vs  Healthy | Reference |
| --- | --- | --- | --- |
| 482 | DNA | + | Biosens. Bioelectron., 2021, 189, 113315  Anal. Bioanal. Chem., 2021, 19, 4475-4784 |
| 496 | Ring vibration /  Tryptophan | - | Biosens. Bioelectron., 2010, 25, 2414-2419  J. Raman Spectrosc., 2022, 53, 1371-1379 |
| 633 | L-tyrosine /  C-S stretch | - | Biomed. Opt. Express, 2015, 6, 33494-502  J. Raman Spectrosc., 2016, 47, 917-925 |
| 739 | DNA /  Tryptophan | + | Biosens. Bioelectron., 2010, 25, 2414-2419  SPECTROCHIM ACTA A, 2021, 246, 119034 |
| 813 | Tyrosine | - | Lasers Med. Sci., 2016, 31, 1317-1324 |
| 884 | δ(C-O-H) / glutathione /  d-(C)-galactosamine | - | Nanomed-Nanotechnol. Biol. Med., 2011, 7, 655-663  Biosens. Bioelectron., 2010, 25, 2414-2419 |
| 963 | Stretching C-O ribose | + | SPECTROCHIM ACTA A, 2021, 246, 119034 |
| 1031 | δ(C-H) Phenylalanine | - | Anal. Bioanal. Chem., 2021, 19, 4475-4784 |
| 1093 | ν(C–N) d-Mannos | + | Biosens. Bioelectron., 2010, 25, 2414-2419 |
| 1131 | C–N stretch /  D-mannose | - | Laser Phys. Lett., 2014, 11, 065603 |
| 1204 | Tryptophan / phenylalanine | - | Lasers Med Sci. 2016, 31, 1317-24  A Review. Molecules, 2023, 28, 2502 |
| 1240 | A, C, or T, ring stretching | + | Appl Spectrosc. 2005, 59, 418-23  Nano Res. 2017, 10, 3662-2670 |
| 1327 | DNA and phospholipids Collagen | + | SPECTROCHIM ACTA A, 2021, 246, 119034 |
| 1446 | δ(CH2) lipids/protein | + | Laser Phys. Lett., 2014, 11, 065603,  J. Raman Spectrosc., 2016, 47, 917-925 |
| 1692 | Citric acid | - | J. Raman Spectrosc., 2007, 38, 1133-11147 |

**Table S12.** Assignment and mechanism of Raman shifts of biomedical hyperspectral images.

| Raman shift  (cm^-1^) | Assignment | Reference |
| --- | --- | --- |
| 733 | Phosphatidylserine | Spectroc. Acta Pt. A-Molec. Biomolec. Spectr. 2005, 61, 1529-1535 |
| 776 | Phosphatidylinositol | Spectroc. Acta Pt. A-Molec. Biomolec. Spectr. 2005, 61, 1529-1535 |
| 1001 | Phenylalanine | Faraday Discuss, 2004, 126, 141-157 |
| 1090 | Symmetric phosphate stretching vibrations | Anal. Methods 2014, 6, 3901-3917 |
| 1129 | *v* (C-C) skeletal of acyl backbone | Microsc. Res. Tech. 2005, 68, 75-79 |
| 1200 | Nucleotides/C-O/C-N | Vib. Spectrosc. 2007, 43, 13-25  J. Forensic Sci. 2007, 52, 88-92 |
| 1224 | Amide III (β-sheet) | Proc Natl Acad Sci U S A 2008, 105, 1410-1415 |
| 1320 | DMA/RNA/CH | Proc Natl Acad Sci U S A 2008, 105, 1410-1415 |
| 1362 | Guanine (N7, B, Z-marker) | Bull. Mat. Sci. 2011, 3 |
| 1451 | CH_2_CH_3_ deformation | Analyst, 2011,137, 322-332 |
| 1460 | Collagen/deoxyribose | Microsc. Res. Tech. 2005, 68, 75-79 |
| 1548 | Amide II | Faraday Discuss, 2004, 126, 141-157 |
| 1564 | COO- | J. Mol. Struct. 1999, 480-481, 1-13 |
| 1658 | Amide I/C-O stretching | Radiat. Res. 2002, 157, 175-182 |
| 2409 | Raman-silent window of biomolecules | Appl. Spectrosc. 2007, Rev. 42, 493-54 |
| 2850 | CH_2_ stretching | Appl. Spectrosc. 2007, Rev. 42, 493-54 |
| 2928 | CH_3_ stretching | Appl. Spectrosc. 2007, Rev. 42, 493-54 |

**Table S13**. Parameters of deep learning-based preprocessing models

| Name | Batch size | #Params | FLOPs | Speed (spectra/s) |
| --- | --- | --- | --- | --- |
| Residual CNN  UNet-1D  RSBPCNN | 32  32  32 | 13.1 M  21.0 M  33.7 M | 13.5 G  23.6 G  33.7 G | ~3 800  ~2 500  ~1 900 |

**Table S14**. Comparison of the cancer diagnostic results based on traditional imaging modalities or Raman spectroscopy.

| **Pathological categories** | **Diagnostic results** | **References** |
| --- | --- | --- |
| HCC/ICC | misdiagnosis rate of HCC for ICC patients with no risk factors for HCC was 24.3% (36 ICC) by CT and MRI | World J. Surg. 2020, 44, 3862-3867 |
|  | misdiagnosis rate of HCC for ICC patients with any risk factors for HCC was between 20.0 and 33.3% (54 ICC) by CT and MRI |  |
| HCC/ICC | misdiagnosis rate of HCC was 52% (25 ICC) by CEUS, 4.2% (24 ICC) and 9.1% (11 ICC) by CT and MRI | Liver Int. 2013, 33, 771-779 |
| High-grade and low-grade HCC | accuracies are 60-70% (170 patients) based on non-contrast-enhanced MRI | Eur. Radiol. 2019, 29, 2802-2811 |
|  | an accuracy of 53.33% (297 subjects) based on contrast-enhanced CT | Eur. Radiol. 2020, 30, 6924-6932 |
| HCC | the sensitivities of US were 63-65% | Aliment. Pharmacol. Ther. 2009, 30, 37-47 |
| HCC | the sensitivities of dynamic CT and MRI were 63%-76% and 77%-90%, and the specificities were 87%-98% and 84%-97% | World J. Gastroenterol. 2018, 24, 2348-2362 |
| Per-lesion for nodular HCC | sensitivity of MRI for nodular HCC of all sizes is 77%–100%, while that of CT is 68%–91%  per-lesion sensitivities are 100% for both modalities for nodular HCCs larger than 2 cm, 44%–47% (MRI) and 40%– 44% (CT) for 1–2 cm HCCs  and 29%–43% (MRI) and 10%–33% (CT) for HCCs smaller than 1 cm | Radiology 2014, 273, 30-50, Hepatology 2003, 38, 1034-1042, and Gut 2010, 59, 638-644 |
| Raman spectroscopy for Liver cancer / paracancer tissues | accuracy 92.6%  sensitivity 90.8%  specificity 94.6% | Nat Commun 2023, 14, 48 (n=120 cancer cases vs 120 healthy cases) |
| **Raman spectroscopy for cancer serum** | **AUC 0.91**  **Sensitivity 85.67%**  **Specificity 87.53%** | **This work (few-shot study, n=27 cancer cases vs 28 healthy cases)** |

**Table S15**. Paraquat concentration prediction$E_{Q}$ of spectra preprocessed by different methods.

| Proportion = 0.5: 9.5 | Concentration (M) | 10^-4^ | 10^-5^ | 10^-6^ | 10^-7^ | Average |
| --- | --- | --- | --- | --- | --- | --- |
|  | Raw spectra  Polynomial fitting  Wavelet transform  Residual CNN*  UNet-1D^*^  RSBPCNN^*^  Residual CNN^#^  UNet-1D^#^  RSBPCNN^#^ | 0.3371  0.3276  0.2908  0.2164  0.1862  0.1649  0.1616  0.1425  **0.1067** | 0.3258  0.3030  0.2457  0.2012  0.1822  0.1805  0.1737  0.1422  **0.1135** | 0.2863  0.3205  0.2728  0.1981  0.1778  0.1592  0.1476  0.1348  **0.1202** | 0.3048  0.3581  0.2511  0.2139  0.1850  0.2021  0.1670  0.1728  **0.1220** | 0.3135  0.3273  0.2651  0.2074  0.1828  0.1767  0.1625  0.1481  **0.1156** |
| Proportion = 1:9 | Raw spectra  Polynomial fitting  Wavelet transform  Residual CNN*  UNet-1D^*^  RSBPCNN^*^  Residual CNN^#^  UNet-1D^#^  RSBPCNN^#^ | 0.1944  0.2344  0.2595  0.1574  0.1644  0.1299  0.1467  0.1197  **0.0950** | 0.1967  0.2675  0.2481  0.1773  0.1626  0.1432  0.1230  0.1277  **0.1019** | 0.2144  0.2450  0.2801  0.1852  0.1494  0.1499  0.1295  0.1159  **0.0945** | 0.2496  0.2635  0.2727  0.1585  0.1487  0.1338  0.1444  0.1483  **0.1106** | 0.2138  0.2565  0.2017  0.1696  0.1563  0.1392  0.1359  0.1279  **0.1005** |
| Proportion = 2:8 | Raw spectra  Polynomial fitting  Wavelet transform  Residual CNN*  UNet-1D^*^  RSBPCNN^*^  Residual CNN^#^  UNet-1D^#^  RSBPCNN^#^ | 0.1989  0.1894  0.1432  0.1299  0.1322  0.1164  0.1033  0.1077  **0.0930** | 0.1970  0.1841  0.1565  0.1353  0.1141  0.1200  0.1063  **0.1029**  0.1039 | 0.2002  0.1734  0.1437  0.1387  0.1356  0.1050  0.1084  **0.1009**  0.1055 | 0.1495  0.1847  0.1414  0.1005  0.1200  0.1107  0.1093  0.1114  **0.0924** | 0.1864  0.1829  0.1426  0.1261  0.1255  0.1130  0.1068  0.1057  **0.0987** |
| Proportion = 4:6 | Raw spectra  Polynomial fitting  Wavelet transform  Residual CNN*  UNet-1D^*^  RSBPCNN^*^  Residual CNN^#^  UNet-1D^#^  RSBPCNN^#^ | 0.0997  0.1498  0.1121  0.1111  0.1176  0.1045  0.0990  **0.0966**  0.1038 | 0.1042  0.1419  0.1181  0.1087  0.1168  0.0965  **0.0898**  0.0967  0.1049 | 0.1059  0.1329  0.1127  0.1070  0.1013  **0.0922**  0.1037  0.1078  0.0991 | 0.1189  0.1234  0.1123  0.1028  0.0967  0.1137  0.1058  0.1133  **0.0794** | 0.1072  0.1370  0.1138  0.1074  0.1081  0.1017  0.0996  0.1036  **0.0968** |
| Proportion = 5:5 | Raw spectra  Polynomial fitting  Wavelet transform  Residual CNN*  UNet-1D^*^  RSBPCNN^*^  Residual CNN^#^  UNet-1D^#^  RSBPCNN^#^ | 0.0942  0.1225  0.1235  0.1139  **0.0989**  0.1058  0.0990  0.1060  0.1006 | 0.0970  0.1381  0.1304  0.0966  0.1136  0.1026  0.0941  0.0966  0.0927 | 0.0896  0.1221  0.1312  0.1049  0.1019  0.0966  0.0947  0.0980  **0.0890** | 0.1112  0.1273  0.1234  0.1094  0.1168  0.0985  **0.0886**  0.0890  0.1012 | 0.0980  0.1275  0.1271  0.1062  0.1078  0.1009  **0.0941**  0.0974  0.0959 |
| Proportion = 7:3 | Raw spectra  Polynomial fitting  Wavelet transform  Residual CNN*  UNet-1D^*^  RSBPCNN^*^  Residual CNN^#^  UNet-1D^#^  RSBPCNN^#^ | 0.1076  0.0920  0.1224  0.1104  0.1091  0.0974  0.1022  **0.1002**  0.1022 | 0.1056  0.0929  0.1041  0.0972  0.1000  0.1080  0.0871  0.0982  **0.0958** | 0.0965  0.0932  0.1064  0.1106  0.0937  0.1046  **0.0856**  0.0905  0.0899 | 0.0919  0.1103  0.1167  0.0919  0.1088  0.0887  0.1019  **0.0879**  0.0885 | 0.1004  0.0971  0.1124  0.1025  0.1029  0.0997  0.0942  0.0942  **0.0941** |
| Proportion = Training set: Validation set (n = 100 spectra per concentration).  ^*^: models trained using mathematical simulation datasets.  ^#^: models trained using RSGAN-generated datasets.  The smallest average $E_{Q}$values are shown in bold. | | | | | | |

1 Goodfellow, I. *et al.* Generative adversarial networks. *Commun. ACM* **63**, 139-144 (2020).
